# Lung Deposition of Surfactant Delivered via a Dedicated Laryngeal Mask Airway in Piglets

**DOI:** 10.3390/pharmaceutics13111858

**Published:** 2021-11-04

**Authors:** Anders Nord, Doris Cunha-Goncalves, Rikard Linnér, Federico Bianco, Fabrizio Salomone, Francesca Ricci, Marta Lombardini, Massimo Micaglio, Daniele Trevisanuto, Valeria Perez-de-Sa

**Affiliations:** 1 Department of Clinical Sciences, Lund University, SE-221 84 Lund, Sweden; anders.nord@med.lu.se (A.N.); doris.cunha_goncalves@med.lu.se (D.C.-G.); rikard.linner@med.lu.se (R.L.); 2 Corporate R&D, Chiesi Farmaceutici S.p.A., 43122 Parma, Italy; f.bianco@chiesi.com (F.B.); f.salomone@chiesi.com (F.S.); f.ricci@chiesi.com (F.R.); m.lombardini@chiesi.com (M.L.); 3 Department of Anesthesia and Intensive Care, Careggi University Hospital, 50134 Florence, Italy; m.micaglio@gmail.com; 4 Child’s Health, University of Padova, 35128 Padova, Italy; daniele.trevisanuto@gmail.com

**Keywords:** surfactant, lung deposition, laryngeal mask airway, newborn, scintigraphy

## Abstract

It is unknown if the lung deposition of surfactant administered via a catheter placed through a laryngeal mask airway (LMA) is equivalent to that obtained by bolus instillation through an endotracheal tube. We compare the lung deposition of surfactant delivered via two types of LMA with the standard technique of endotracheal instillation. 25 newborn piglets on continuous positive airway pressure support (CPAP) were randomized into three groups: 1—LMA-camera (integrated camera and catheter channel; catheter tip below vocal cords), 2—LMA-standard (no camera, no channel; catheter tip above the glottis), 3—InSurE (Intubation, Surfactant administration, Extubation; catheter tip below end of endotracheal tube). All animals received 100 mg·kg^−1^ of poractant alfa mixed with ^99m^Technetium-nanocolloid. Surfactant deposition was measured by gamma scintigraphy as a percentage of the administered dose. The median (range) total lung surfactant deposition was 68% (10–85), 41% (5–88), and 88% (67–92) in LMA-camera, LMA-standard, and InSurE, respectively, which was higher (*p* < 0.05) in the latter. The deposition in the stomach and nasopharynx was higher with the LMA-standard. The surfactant deposition via an LMA was lower than that obtained with InSurE. Although not statistically significant, introducing the catheter below the vocal cords under visual control with an integrated camera improved surfactant LMA delivery by 65%.

## 1. Introduction

Respiratory distress syndrome (RDS) [1] is the most common cause of respiratory insufficiency in preterm infants. Even though mortality rates and the frequency of pneumothorax decreased with the introduction of surfactant treatment, the incidence of bronchopulmonary dysplasia (BPD) remains unchanged [2]. Invasive mechanical ventilation is recognized as one of the modifiable risk factors for developing BPD [3]. To avoid or shorten the exposure to mechanical ventilation, less invasive modes of respiratory support and surfactant administration have been successfully introduced in recent decades [4]. In 1992, Verder et al. [5] described the InSurE procedure, which consisted of tracheal INtubation, SURfactant delivery through the endotracheal tube, and Extubation after a brief period of mechanical ventilation. Currently, particularly in Europe [6], surfactant is increasingly administered to neonates on noninvasive respiratory support through a feeding tube or a small catheter placed into the trachea with the aid of laryngoscopy (LISA) [7,8]. Compared to InSurE, a less invasive surfactant administration (LISA) decreases mortality, the need for mechanical ventilation, and BPD rates [9,10]. Unfortunately, LISA techniques require laryngoscopy, a painful procedure that demands special skills [11] and is potentially associated with hemodynamic instability, hypoxia, and increased intracranial pressure [12]. Besides nebulization [13,14], another proposed mode for surfactant delivery in preterm infants is the use of a laryngeal mask airway (LMA) [15,16], a supraglottic airway device. This technique’s advantage is reducing invasiveness by precluding laryngoscopy during catheter positioning and requiring less training for proper placement than laryngoscopy and endotracheal intubation. The LMA has been endorsed in neonatal resuscitation guidelines since 2000 [17,18], and is currently routinely used during the sedation and anesthesia of newborns [19]. In a recent systematic review and meta-analysis [20], the administration of surfactant to premature babies via an LMA decreased the rate of intubation and invasive mechanical ventilation.

In our experimental model in newborn piglets, we hypothesized that, compared to the bolus administration of surfactant in the central lumen of a standard LMA, the amount of drug (phospholipids) delivered to the lungs would be higher when surfactant was dispensed below the vocal cords with a catheter introduced under visual control through a customized LMA bearing an integrated video-capable camera. The standard instillation method via an endotracheal tube was used as a control.

## 2. Materials and Methods

After approval by Lund University (M69-14, 150414), 25 newborn term piglets (weight 1.2–2.3 kg) received care following the European guidelines for care and use of laboratory animals (Directive 2010/63/EU).

The animals were premedicated with 3 mg of ketamine, 0.4 mg of midazolam, and 0.1 mg of atropine i.m. An ear vein was cannulated, and the animals were placed supine on a vacuum mattress, in an open neonatal incubator (Babytherm, Dräger, Lubeck, Germany), with the head slightly elevated (30 degrees). Analgosedation was obtained by infusions of dexmedetomidine (1–3 μg·kg^−1^·h^−1^) and ketamine (1–3 mg·kg^−1^·h^−1^) throughout the experiment. Under local anesthesia (lidocaine) and ultrasound guidance, an artery in the lower limb was cannulated. Bolus doses of propofol (0.5–1 mg·kg^−1^) and remifentanil (0.5–1 μg·kg^−1^) were given whenever necessary to keep the animal comfortable and without pain.

Mean arterial blood pressure (MAP) and heart rate (HR) were continuously monitored and recorded. Near-infrared regional cerebral oximetry (crSO_2_) (INVOS 5100C, Somanetics Corporation, Troy, MI, USA), peripheral oxygen saturation, and rectal temperature were continuously monitored and recorded (Philip IntelliVue M70, Philips Medizin Systeme, Boeblingen, Germany). After 5 min of stabilization in the supine position, while spontaneously breathing room air, a baseline blood gas was obtained (BG1). The subject was then connected to a humidified (MR850, Fisher & Paykel Healthcare Ltd., Auckland, Australia) dual limb circuit (EVAQUA™, Fisher & Paykel Healthcare Ltd., Auckland, Australia) of the Servo-*i* ventilator (Getinge Sverige AB, Getinge, Sweden) using a customized nasal mask placed over the animal’s snout. The ventilator settings were infant mode, noninvasive ventilation, nasal continuous positive airway pressure (CPAP) 3–4 cm H_2_O, and inspired oxygen (FiO_2_) 0.4. Ventilator parameters tidal volume (VT), minute volume (MV), air leak, and respiratory rate (RR) were collected from the user interface on the Servo-*i* ventilator.

In a pilot study, we tested different prototypes of a newly designed laryngeal mask equipped with an endoscopic camera and an independent channel to facilitate the introduction of a surfactant delivery catheter. The prototype chosen was the one that best fitted the anatomy of the piglet.

**Figure 1:**
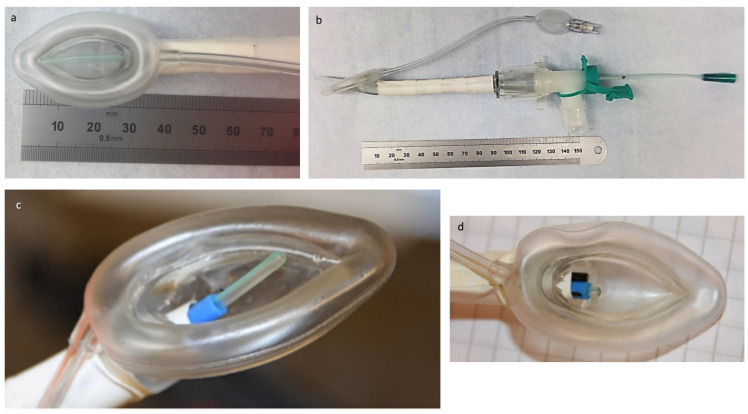
Laryngeal mask prototypes used in the study. (**a**,**b**) Panels: modified laryngeal mask airway of type Unique, size 1, with swivel and catheter; (**c**,**d**) panels, modified laryngeal mask of type Unique, size 1, with integrated catheter channel (blue) and camera (black).

The Size 1 LMA Unique (Teleflex Medical, Dublin, Ireland) was modified to simulate the proposed features of the LMA for surfactant administration and provide an LMA suited for the anatomy of the porcine model. The core changes were: 1—A stiffening element applied to the outside surface of the LMA, which allowed us to adjust the curvature of the LMA during the procedure. In practice, it was predominantly used to fully straighten and increase the stiffness of the LMA type Unique to make it appropriate for the anatomy of the porcine model and aid insertion; 2—An internal catheter lumen (blue tubing on Figure 1c,d) was added for the catheter to pass through so that it was directed towards the laryngeal inlet and vocal cords; 3—A camera mounted side-by-side with the catheter lumen exit point to provide a clear view of the tracheal inlet and vocal cords and to observe the catheter advancement towards the target; 4—A modified elbow connector, a swivel, at the proximal 15 mm interface to allow for connection to the breathing circuit while also allowing the catheter lumen and camera to be inserted into the LMA device without substantial levels of leakage.

**Figure 2:**
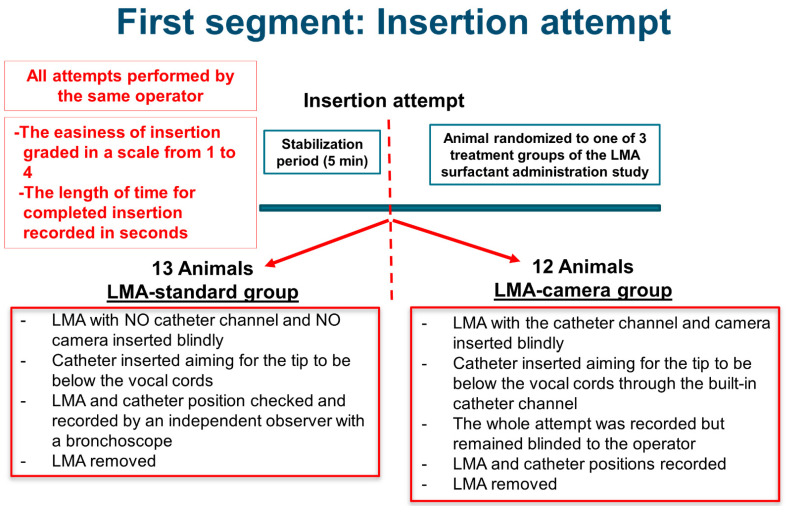
Summary of the first segment (Insertion attempt) of the experimental study protocol. LMA, laryngeal mask airway.

The study protocol was divided into two separate, independent segments denominated “Insertion attempt” (first segment) and “LMA surfactant administration,” respectively. The first segment was designed to assess the feasibility of blindly passing a delivery catheter past the vocal cords using an LMA as a steering conduit. We tested two different LMAs (“Insertion Attempt,” Figure 2). After the stabilization period, all studied animals were randomized into two groups using one of two types of LMA (Figure 1): LMA-camera group (with a camera and a dedicated catheter channel; Figure 1c,d) and LMA-standard group (no camera, no catheter channel; Figure 1a,b). The same operator performed all insertion attempts in both groups, but had no access to the video recordings taken during and after the procedure. We lubricated the tip of the LMAs with lidocaine gel to facilitate the introduction and give more comfort to the animal. In the LMA-camera group (*n* = 12 animals), when the LMA was in place, we inserted the delivery catheter through the dedicated built-in channel to a predetermined length so that the tip was expected to be inside the trachea. Although the LMAs with the catheter channel also had a built-in camera, the continuous video recordings of the procedure were concealed from the operator. In the LMA-standard group (*n* = 13 animals), we positioned the LMA and then inserted the catheter through the central lumen to a predetermined length so that the tip was expected to pass the vocal cords. In this group, when the delivery catheter was already in place through the central lumen, the position of the LMA and catheter was documented and recorded by an independent observer with a 3.8 mm disposable fiber bronchoscope (Ambu^®^aScope™ 4 Broncho Slim, Ambu A/S, Ballerup, Denmark).

We recorded the time from the moment the operator started the LMA insertion process until the catheter placement was judged satisfactory by the blinded operator. We studied the easiness of insertion by asking the operator to grade the insertion process with a scale from 1 to 4 (1 = very easy and 4 = very difficult). The success rate of the correct placement of the catheter tip below the vocal cords was studied by a posterior analysis of the video recordings obtained by the independent observer.

**Figure 3:**
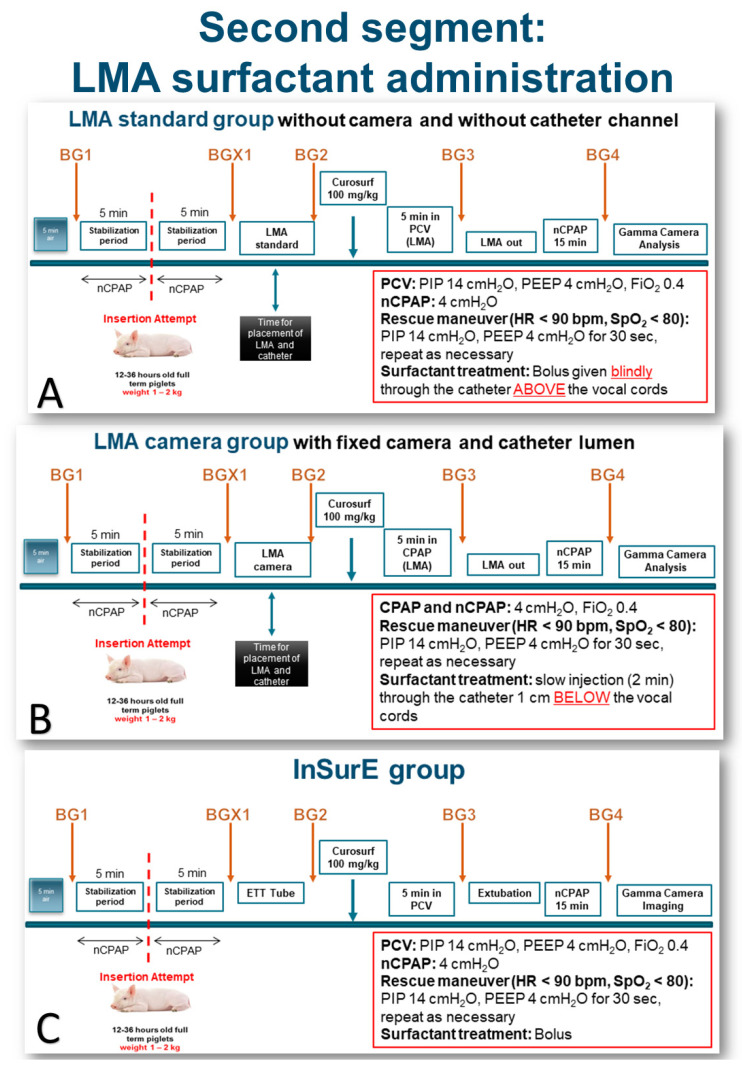
Summary of the second segment of the experimental protocol “LMA surfactant administration”. Panel (**A**), LMA standard group; panel (**B**), LMA camera group; panel (**C**), InSurE group. LMA, laryngeal mask airway; BG, blood gas; CPAP, continuous positive airway pressure; nCPAP, nasal CPAP; FiO_2_, inspired fraction of oxygen; HR, heart rate; SpO_2_, peripheral oxygen saturation; PIP, peak inspiratory pressure; PEEP, positive end-expiratory pressure; PCV, pressure-controlled ventilation; ETT, endotracheal tube.

After the “First Attempt” segment of the study, the LMA was withdrawn, the animal was kept on nCPAP for 5 min, and then randomized to one of three groups in the second segment of the study, “LMA surfactant administration” (Figure 3A–C): 1—LMA-camera (Figure 1c,d), 2—LMA-standard (Figure 1a,b), and 3—InSurE. A second blood gas (BGX1) was obtained (Figure 3A–C).

**Figure 4:**
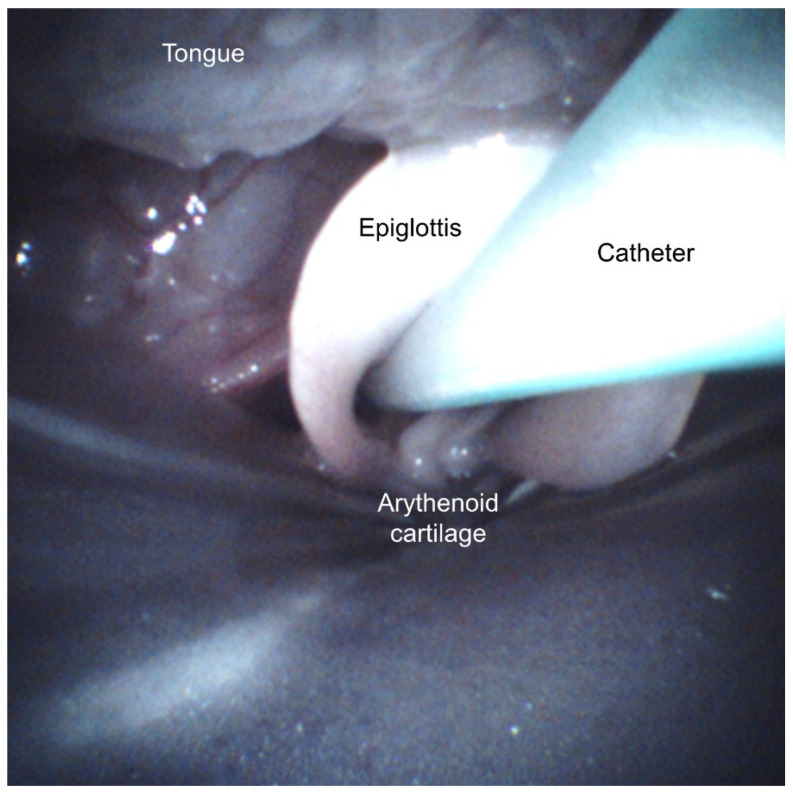
Picture of the correct position of the laryngeal mask airway. The picture was obtained from the video recording during insertion.

**Figure 5:**
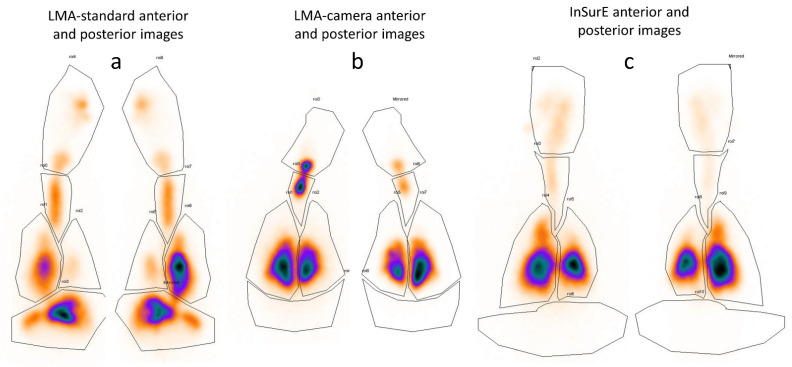
Typical images obtained from the gamma camera for one animal in each group. Panel (**a**), anterior and posterior images of a subject in the group with the laryngeal mask airway standard; panel (**b**), anterior and posterior images of a subject in the group with the laryngeal mask airway with a camera; panel (**c**), anterior and posterior images of a subject in the group with the intubation-surfactant-extubation (InSurE). The median time between surfactant administration and imaging was 52 min (41–91), median (range).

The LMA-camera group included nine spontaneously breathing animals on nasal CPAP (Figure 3B). The laryngeal mask was inserted under the guidance of the integrated camera to ascertain the correct position of the device at the laryngeal entrance, i.e., with the epiglottis and the glottis free at the center of the LMA opening (Figure 4). The Y-piece of the ventilatory circuit was connected to the LMA. The delivery catheter was advanced in its channel until the tip passed 1 cm below the vocal cords. Just before the surfactant administration, a new blood gas was obtained BG2 (Figure 3B). 100 mg·kg^−1^ of poractant alfa (Curosurf^®^, Chiesi Farmaceutici, Parma, Italy), 1.25 mL·kg^−1^ (80 mg·mL^−1^), previously mixed with Technetium-labelled nanocolloid particles (100 MBq) was slowly injected through the catheter (about 1–2 min) while the camera was recording. The catheter was removed, the LMA was kept in place for five minutes, and the piglets were supported with CPAP. Blood gas number 3 (BG3) was obtained, and the LMA was removed. The animal was kept spontaneously breathing on nasal CPAP for another 15 min. Blood gas number 4 (BG4) was taken, and the animal moved to the gamma camera for the acquisition of planar anterior-posterior and posterior-anterior pictures (Figure 5).

In the LMA-standard group (*n* = 8) (Figure 3A), the insertion was blind, and the delivery catheter was introduced through a swivel connector in the device’s central lumen, also blindly. The length of the catheter was adjusted so that the tip was just on the edge of the LMA opening, that is, above the vocal cords. Another observer recorded the final position of the LMA and catheter with a fiber bronchoscope without revealing the result to the main operator. Before treatment, BG2 was taken. An equal amount of surfactant mixed with Technetium-labeled nanocolloid was delivered as a bolus above the vocal cords, and the animal was immediately ventilated with pressure-controlled ventilation for 5 min. The ventilator settings were an inspiratory pressure of 10 cm H_2_O above 4 cm H_2_O PEEP, with a RR of 30 breaths·min^−1^ and FiO_2_ 0.4. After 5 min, the LMA was removed, and nasal CPAP resumed. Blood gas 3 was obtained, and the animal was supported with nasal CPAP for another 15 min. Blood gas 4 was taken just before the animal was transferred to the gamma camera.

In the InSurE group (*n* = 8) (Figure 3C), after randomization, hemodynamic measurements, together with a blood gas (BGX1), were obtained after keeping the animals on nasal CPAP for 5 min as with the other two groups. Under continuous sedation and spontaneous breathing, laryngoscopy was performed, and local anesthesia with lidocaine spray was applied to the larynx before endotracheal intubation. One operator managed all intubations. We checked the position of the tube by auscultation and, briefly, by visual control with a fiber bronchoscope before instillation. The surfactant was delivered as a bolus through a catheter with its tip 1 cm below the end of the endotracheal tube. Without bag ventilation, the endotracheal tube was immediately connected to the ventilator circuit. The settings were adjusted for pressure-controlled ventilation with an inspiratory pressure of 10 cm H_2_O above 4 cm H_2_O PEEP, FiO_2_ 0.4, and RR 30 breaths·min^−1^ for 5 min when BG3 was obtained. Upon regaining stable breathing, the animal was extubated to nasal CPAP. After 15 min on nasal CPAP support, the last blood gas was obtained (BG4), just before transport to the gamma camera.

For all groups, a rescue maneuver was defined a priori, per protocol, in case of inadequate breathing or bradycardia (<90 bpm) and/or desaturation below 80% during or after surfactant administration: pressure-controlled ventilation via the LMA, endotracheal tube, or nasal mask, with an inspiratory pressure of 10 cm H_2_O above 4 cm H_2_O PEEP (positive end-expiratory pressure), with a respiratory rate of 30 breaths per minutes for 30 s.

After image acquisition in the gamma camera, the piglets were killed with an overdose of thiopental, fentanyl, and potassium chloride.

### Measurement of Surfactant Deposition and Distribution

The deposition, as a percentage of the administered dose, was calculated as described previously [21]. Briefly, the piglets were placed supine in a gamma camera (Philips Skylight, Philips AB, Stockholm, Sweden) with dual heads, simultaneously acquiring one anterior and one posterior image. These were analyzed as 128 × 128 matrices with pixel size 3.2 mm. After a 3-min exposure, approximately 25 MBq of ^99m^Tc-macro aggregated albumin (Tc-MAA, TechneScan LyoMAA, Covidien Sverige AB, Solna, Sweden) was injected intravenously, and a second exposure was made. The Tc-MAA is trapped in the lung capillaries and is used to outline the lungs, as well as to do an internal calibration. There is a linear relationship between the amount of radioactivity used in the study and the counts detected by the gamma camera. Using a linear equation with an offset of 0, we calculated the slope of the line from the change in counts when introducing a known amount of radioactivity into the image field. The slope could then be used to calculate the amount of radioactivity in the different regions of interest (ROI) using the following equation:

Deposition (ROI) = 100 × {Counts (ROI)/[Counts (2) − Counts (1)]} × [Tc-MAA/TcNanocolloid]

where Counts (n) are the counts in the whole gamma camera image field before (1) and after (2) the Tc-MAA was administered to the piglet. The change in radioactivity, before (1) and after (2), is equal to the amount of TcMAA administered intravenously (known amount). Using the equation above, it is possible to calculate the deposition in an ROI as a percentage of the amount of ^99m^technetium-labeled nanocolloid (TcNanocolloid) and surfactant administered where Counts (ROI) are the number of counts in the ROI before Tc-MAA has been administered. The exact amount of radioactivity was measured with a Geiger counter for doses and residuals. All counts and doses were corrected for the 6-h half-life of ^99m^Tc. The net amount of TcNanocolloid and Tc-MAA were calculated after residual radioactivity in syringes, mixing vials, and catheters have been subtracted. The deposition was calculated at the various sites from the mean of the anterior and posterior images and presented as a percentage of the administered surfactant dose (Supplementary Material: all acquired images and delineated ROIs in all 3 groups before and after Tc-MAA).

Statistical methods: The Sigmaplot 14 software (Systat Software Inc., San Jose, CA, USA) was used for the statistical analysis. Assuming a 10% difference in the mean values for the deposition in the lungs, an SD of 5% with an alfa value of 0.05, and a power of 0.8, the minimum sample size would be six in each of the three treatment groups. For an analysis of the data obtained in the first segment of the study (*Insertion Attempt*), the *t*-test or the Mann–Whitney rank-sum test were used when appropriate. For the second segment (*LMA surfactant administration*) of the study, significant differences among the three groups were investigated using One-way ANOVA or ANOVA on Ranks when normality testing failed, followed by a post hoc test when indicated. A *p*-value < 0.05 was considered significant. Data are reported as median (range) when not otherwise stated [22].

## 3. Results

### 3.1. Insertion Attempt Study

The time for the blind insertion of an LMA and a delivery catheter was 83 s (49–250) in the LMA-camera group and 72 s (54–398) in the LMA-standard group, respectively (not statistically significant). The ease-of-insertion rating was not significantly different between the groups: 2 (1–3) and 1 (1–4) in the LMA-camera and LMA-standard groups, respectively. There were no significant within- or between-group changes in hemodynamics and oxygenation during the attempts (data not shown). After reviewing all 25 video recordings, the glottis entrance was confirmed to be appropriately enclosed by the LMA in less than 50% of all blind attempts (5/12 LMA-camera group, 7/13 LMA-standard group). None of the catheters in the LMA-camera group passed the vocal cords, compared to three in the LMA-standard group (Figure 4).

### 3.2. LMA Surfactant Administration Study 

The median weight of the animals was comparable between groups: 1.7 kg (1.5–2.3), 1.8 kg (1.2–2.1), and 1.8 kg (1.2–2.0) in the LMA-camera, LMA-standard, and InSurE groups, respectively. There were no significant differences between groups in the times for device insertion or intubation, 141 s (58–420) in the LMA-camera group, 73 s (39–528) in the LMA-standard group, and 71 s (30–151) in the InSurE group. There were no significant differences between the groups in the easiness grading of LMA insertion: 1.5 (1–4) and 2 (1–4) in the LMA-standard and LMA-camera groups, respectively. Using the integrated camera to guide the LMA and catheter insertion, 89% of the catheters were correctly positioned in the LMA-camera group. The posterior analysis of the video recordings in this group clearly showed that, in one animal, we misinterpreted the catheter position. The catheter tip was trapped in the vestibular fold, resulting in the administered surfactant’s immediate reflux and, consequently, the lowest lung deposition in the group (10%). In the other subject with a low deposition (23%) in the LMA-camera group, the reflux of the injected surfactant could be observed during a brief period of apnea.

We did not see any noticeable damage to the soft tissues around the glottis during treatment.

There were no significant differences between the groups in blood gases 1 (baseline) or 4 (before transport to the gamma camera) (Table 1).

Two animals in each LMA group, and three in the InSurE group, needed a rescue ventilation maneuver as per the protocol (Figure 3) due to apnea, bradycardia, or desaturation.

**Figure 6:**
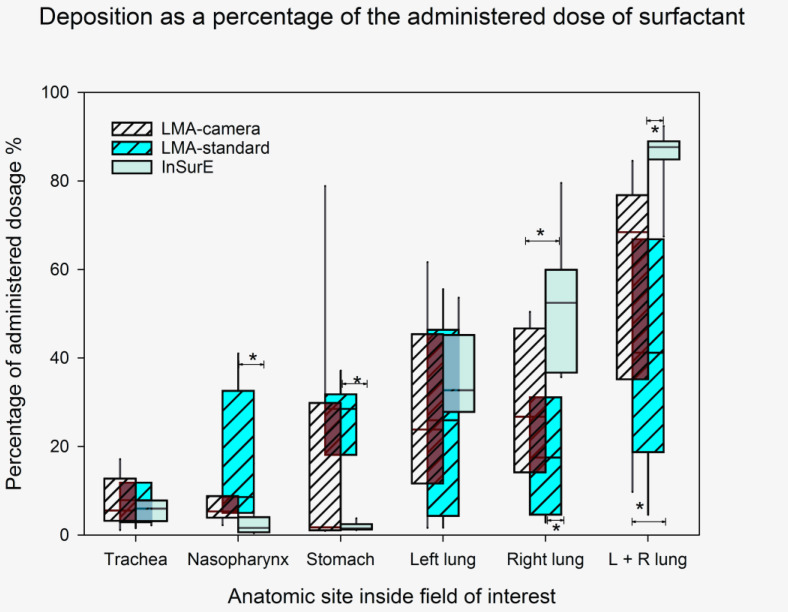
Deposition as a percentage of the administered dose. Box plot (median, 5th, 95th percentiles) for the deposition calculated as a percentage of the administered dose for the different anatomical sites inside the region of interest. * is *p* < 0.05 (analysis of variance with Dunn’s post hoc test).

Figure 5 shows typical scintigraphy deposition images from one animal in each group. The median of the total lung deposition (Figure 6 and Table 2) was higher in the InSurE group. The deposition in the stomach and nasopharynx was higher in the LMA-standard group.

## 4. Discussion

In spontaneously breathing piglets on CPAP support, the presence of an integrated channel for the surfactant delivery catheter in an LMA, by itself, did not increase the success rate of positioning the catheter tip below the vocal cords when the procedure was done blindly (no visual assistance). However, the ability to properly place the delivery catheter improved from 12 to 89% when employing the visualization of the laryngeal structures in the LMA-camera group. The camera images allow the precise alignment of the LMA with the glottic opening, and enable the introduction of the catheter through the vocal cords under continuous visual control. Nevertheless, our main hypothesis, that an LMA equipped with an integrated camera and a catheter channel would result in a larger lung surfactant deposition compared to a bolus administration via a standard LMA, could not be statistically confirmed in this study. Moreover, the total lung deposition in the LMA groups was significantly lower than in the InSurE group. However, in contrast to the LMA-standard group, the median amount of surfactant lost to the stomach and nasopharynx in the animals treated with the LMA-camera was small and no different from that observed in the intubated piglets.

Although advantageous compared to the InSurE method, less invasive surfactant administration techniques still require learned skills for laryngoscopy and visualization of the glottis entrance for its success. The current neonatal care clinical guidelines [23] advising the use of noninvasive ventilatory support instead of intubation and mechanical ventilation has decreased the opportunities for neonatologists to secure the necessary proficiency in airway management and intubation [24]. Herrick et al. [25], using data from an international registry study, showed a first-attempt success rate for endotracheal intubation of 49% in the neonatal intensive care unit and 46% in the delivery room. Comparatively, in the case series by Smee et al. [26], the first-attempt success rate for the correct placement of an LMA in neonates was 78% and 98% on the first and second attempts, respectively. The laryngeal mask airway is a supraglottic airway device which has been used in anesthetic practice since the late 1980s [27], either in spontaneously breathing patients or during positive-pressure ventilation, where it is considered a minimally invasive, well-established alternative to the endotracheal tube [11]. The European Resuscitation Council Guidelines 2021 [28], based on the International Liaison Committee on Resuscitation guidelines from 2020 [29], proposes the LMA as an option in newborns with a birth weight >2000 g when face-mask ventilation is unsuccessful, or when intubation is not viable.

In our study, all animals tolerated the insertion of the LMA well, with no observed changes in hemodynamics and oxygenation (MAP, HR, SaO_2_, crSO_2_) during the insertion procedure (data are not shown). The first anecdotal reports on using an LMA in premature babies for surfactant replacement therapy date from 1992 [30]. To the present date, 154 infants have received surfactant replacement therapy through an LMA in the context of randomized controlled trials. There was a positive physiological effect in all five studies, with a reduction of oxygen requirement upon treatment [31,32,33,34,35]. In 2017, Vannozzi et al. [36] described a modified minimally invasive surfactant technique (MIST), Catheter and Laryngeal Mask Endotracheal Surfactant Therapy (CALMEST). Using a dummy, they tested a combination of LISA and MIST techniques, blindly inserting a delivery catheter through a particular LMA used as a guide. They then went on to successfully apply the method to four premature babies. The success rates they reported in the mannequin tests could not, however, be reproduced by others [37]. The proper positioning of an LMA is typically monitored by observing good chest expansion, bilateral auscultation of the thorax, and the presence of CO_2_ elimination by capnography. Others have observed that, despite a normal capnography tracing and unobstructed ventilation, the rate of proper alignment is low [38]. In line with Bonadies et al.’s [37] observations, we demonstrated that even when an experienced operator blindly introduced the LMA, a failure to obtain an accurate position of the device occurred in 50% of the attempts using the standard control procedures for verifying proper placement.

Moreover, in only 3 of these 25 blind attempts to insert the catheter into the trachea, would the surfactant administration have been correctly delivered below the vocal cords. In analogy to using a video-laryngoscope, visual guidance using the integrated camera in the customized LMA could overcome this obstacle. Besides confirming and assisting in the optimal alignment of the device in relation to the glottic opening, the camera also allows continuous visual control during catheter insertion. It also enables a better understanding of the sources of treatment failure. By replaying the recorded videos, one could distinctly identify a misplaced catheter in one animal, and a period of apnea in another, both resulting in massive reflux. One can speculate that this could also happen during LISA and MIST attempts in babies with RDS.

We decided that the tip of the catheter would be 1 cm below the vocal cords as per protocol. Based on recent clinical data suggesting that a slow injection (1 to 3 min) would be more effective than a bolus [39], we chose to administer the surfactant mixture in the LMA-camera group as a 1–2 min injection. It is possible that, while still avoiding the insertion into one of the main bronchi, placing the catheter further down in the trachea would have minimized the amount of reflux, thus resulting in a larger lung dose, as suggested in some of the LISA trials [39]. In the LMA-camera group, to be as gentle as possible, and in analogy with what is desirable in the management of critically ill premature babies with RDS, we placed the catheter into the trachea with the animals spontaneously breathing on CPAP support only. One could argue that a brief period of synchronized pressure support ventilation could have assisted the distribution of surfactant deeper into the lungs and lessened the amount of reflux of the surfactant delivered below the vocal cords. Although not statistically significant, the median deposition in the LMA-camera group was 65% higher than in the LMA-standard group.

Niemarkt et al. [40], using samarium-labeled surfactant in preterm lambs, reported that LISA resulted in only 18% of the lung deposition obtained with endotracheal administration. Nonetheless, they still observed a physiological effect after the treatment. Using scintigraphy, the gold standard method for assessing lung deposition, we reproduced the CALMEST study, while properly assuring the catheter was below the vocal cords, and determined the lung dose of surfactant. In the LMA-camera group, lung deposition was approximately 75% of the InSurE group, which contrasts with Niemarkt et al.’s [40] LISA technique. To our knowledge, there are no earlier reports on the amount of surfactant deposited in the lungs using an LMA or MIST. Recently, Ricci et al. [41], using desaturated-phosphatidylcholine quantification in bronchoalveolar lavage samples as a proxy method for surfactant deposition, found no difference between the LISA and InSurE techniques in a rabbit model.

Contrary to our initial conviction, visually monitoring the introduction of the LMA and the catheter did not guarantee an equivalent lung dose to the one measured after the endotracheal instillation. Even after eliminating the two animals with the lowest lung doses, the median lung deposition in the LMA-camera group is still 20% lower than in the InSurE group. However, the lung dose in the LMA groups, even though smaller, is still within the range expected to elicit a physiological response [34,38]. The absence, or very little, reflux observed in our InSurE group is undoubtedly due to the use of cuffed endotracheal tubes, and contrasts to the higher amount of reflux noticed during the endotracheal instillation in premature babies using uncuffed tubes [32,34].

The introduction of an LMA without sedation or analgesia seems to produce less discomfort and hemodynamic changes than laryngoscopy [11] and, in low resource countries or when the attending physician does not possess the necessary competence to perform laryngoscopy, using a standard LMA, which is easier to place than an endotracheal tube, and administering surfactant as a bolus in the central lumen could probably be of benefit. Still, this hypothesis will have to be tested in larger clinical trials.

The present study has some limitations. The LMA prototype used was adapted to the piglets’ anatomy. The animals were 12–36 h-old full-term newborn healthy piglets that were not surfactant depleted, which can have an impact on surfactant distribution in the lungs. Airway manipulation for surfactant treatment is often carried out with little or no sedation or analgesia in premature babies with RDS. Still, there is a need for sedation due to ethical constraints during animal research, which might not agree with the current standard practice in many neonatal units. Finally, we cannot draw any conclusions regarding the treatment’s physiological effect irrespective of the deposition obtained.

## 5. Conclusions

The success rate for the correct placement of a surfactant delivery catheter beyond the vocal cords via an LMA improved from 12% (3 of 25) to 89% (8 of 9) by incorporating a catheter channel and a camera into an LMA. Lung surfactant deposition obtained with the LMA-camera in piglets on CPAP was 65% higher than that obtained after surfactant delivery above the vocal cords through a standard LMA followed by a few minutes of pressure-controlled ventilation, but this difference was not statistically significant. Nevertheless, administering surfactant below the vocal cords without positive pressure ventilation is undoubtedly clinically valuable. The method can also limit the amount of surfactant that ends up in the nasopharynx and stomach. In both LMA groups, the obtained surfactant lung dose was inferior to that observed after endotracheal instillation. Still, the amount of phospholipids found in the lungs in all groups should be enough to elicit a physiological effect. Our findings support the few clinical trials and case reports present in the literature which show that an LMA can be a feasible way to deliver surfactant to the lungs.

## Figures and Tables

**Table 1:** Blood gases.

Blood Gas Parameter	Baseline (BG1)	Before Instillation (BG2)	5 Min after Instillation (BG3)	20 Min after Instillation (BG4)
**InSurE (Intubation-Surfactant-Extubation)**				
SaO_2_ (*%*)	98 (93–100)	100 (98–100)	99 (93–100)	97 (85–100)
PaO_2_ (mm Hg)	65 (56–74)	91 (67–157) *	84 (53–126)	68 (44–107)
PaCO_2_ (kPa)	38 (32–41)	38 (28–50)	31 (26–43) *	45 (32–53) *
pH	7.54 (7.48–7.58)	7.53 (7.43–7.58)	7.56 (7.47–7.69)	7.46 (7.35–7.52) *
**LMA-standard (no camera, no catheter channel)**				
SaO_2_ (%)	98 (95–100)	100 (97–100)	100 (99–100) *	100 (94–100)
PaO_2_ (mm Hg)	71 (60–75)	107 (83–180) *	110 (73–193)	101 (55–171)
PaCO_2_ (mm Hg)	36 (32–47)	39 (35–52)	32 (22–42)	39 (36–59)
pH	7.54 (7.45–7.56)	7.51 (7.38–7.58)	7.61 (7.50–7.75) *	7.48 (7.36–7.58)
**LMA-camera (with built-in camera and catheter channel)**				
SaO_2_ (%)	97 (86–100)	100 (92–100)	96 (76–100)	97 (95–100)
PaO_2_ (mm Hg)	62 (44–71)	92 (62–151) *	65 (45–119) †	71 (59–157)
PaCO_2_ (mm Hg)	39 (37–44)	50 (39–64) *,†	52 (32–75) *,†	42 (36–53)
pH	7.48 (7.45–7.56)	7.41 (7.33–7.51) †	7.38 (7.33–7.51) †	7.45 (7.39–7.55)

Data are presented as median (range). * denotes *p* < 0.05 inside groups, Repeated Measures Analysis of Variance on ranks with Bonferroni’s post hoc test for stage vs. baseline. † *p* < 0.05 between groups with One Way Analysis of Variance and Students–Newman–Keuls post hoc test.

**Table 2:** Surfactant distribution as a percentage of the total administered dose.

Group	Trachea	Nasopharynx	Stomach	Left Lung	Right Lung	Both Lungs
LMA-camera %	5.6 (1.2–17.2)	5.4 (2.3–12.5)	1.8 (1–78.9)	23.9 (1.7–61.7)	26.8 * (5.3–50.5)	68.5 * (9.8–84.6)
LMA-standard %	8 (2.9–12.8)	8.7 * (3.3–41.1)	28.6 * (1.6–37.2)	26 (1.7–55.6)	17.6 * (2.9–42.7)	41.2 * (4.6–88)
InSurE %	6 (2.3–9.1)	1.7 (0.5–16.2)	1.6 (1.2–3.9)	32.8 (8.2–53.7)	52.5 (35.7–79.6)	87.7 (67.5–92.4)

Data are presented as median (range). * denotes *p* < 0.05 between the group and InSurE with One Way Analysis of Variance and all pairwise multiple comparison procedures with Dunn’s post hoc test.

## Data Availability

The original data presented in this article are available on request from the corresponding author.

## Supplementary Materials

The following are available online at https://www.mdpi.com/article/10.3390/pharmaceutics13111858/s1, all scintigraphy images are available as supplementary material.
