# Supplementary material for: Lung Deposition of Surfactant Delivered via a Dedicated Laryngeal Mask Airway in Piglets
Source: Pharmaceutics. 2021 Nov 4;13(11):1858. doi: 10.3390/pharmaceutics13111858 (PMC8621675; doi:10.3390/pharmaceutics13111858)
Supplement: Supplementary file 1 [file pharmaceutics-13-01858-s001.zip › pharmaceutics-1401432-supplementary.pdf]

pharmaceutics

MDPI

Supplementary Materials: Lung Deposition of Surfactant Delivered via a Dedicated Laryngeal Mask Airway in Piglets

Anders Nord, Doris Cunha-Goncalves, Rikard Linnér, Federico Bianco, Fabrizio Salomone, Francesca Ricci, Marta Lombardini, Massimo Micaglio, Daniele Trevisanuto and Valeria Perez-de-Sa

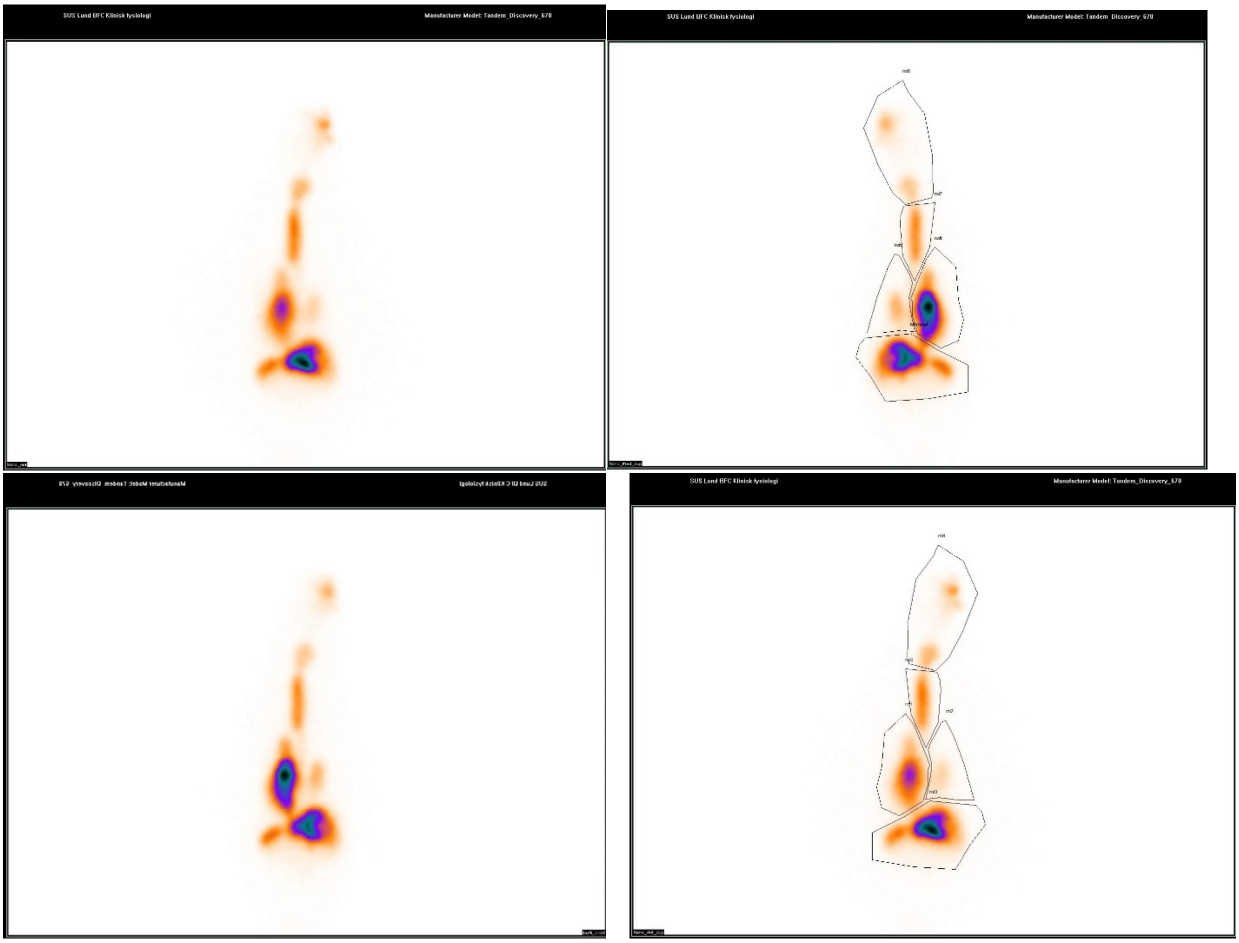

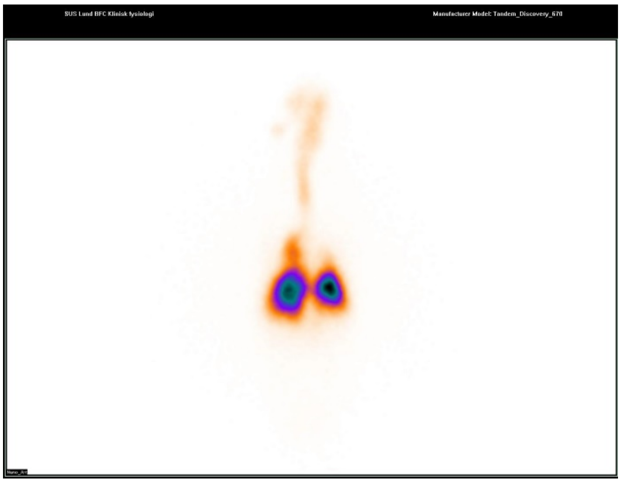
SUS Lund BFC Klinisk fysiologi
Manufacturer Model: Tandem_Discovery_670

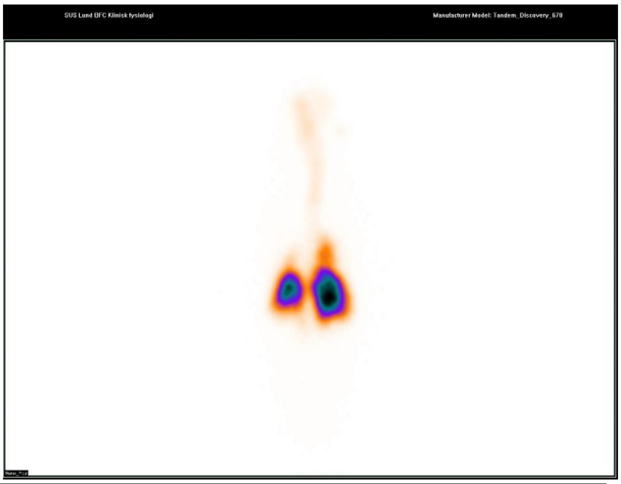
SUS Lund BFC Klinisk fysiologi
Manufacturer Model: Tandem_Discovery_670

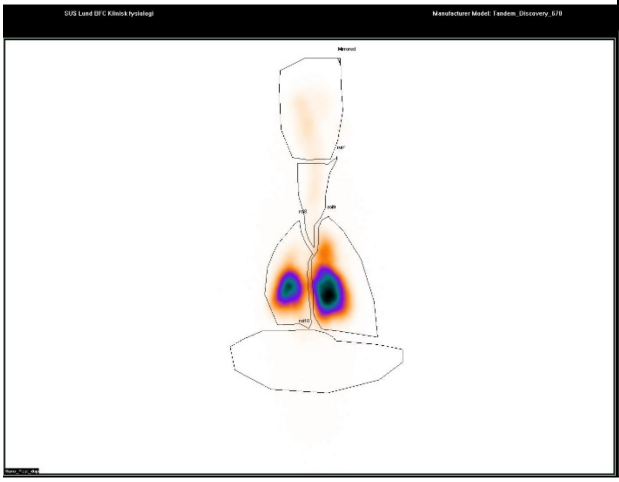
SUS Lund BFC Klinisk fysiologi
Manufacturer Model: Tandem_Discovery_670

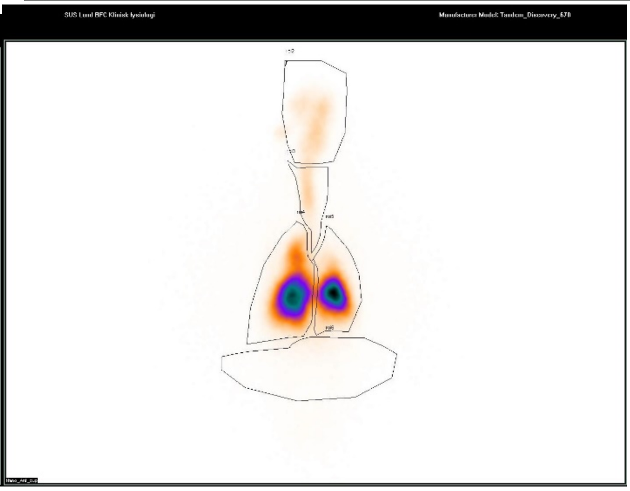
SUS Lund BFC Klinisk fysiologi
Manufacturer Model: Tandem_Discovery_670

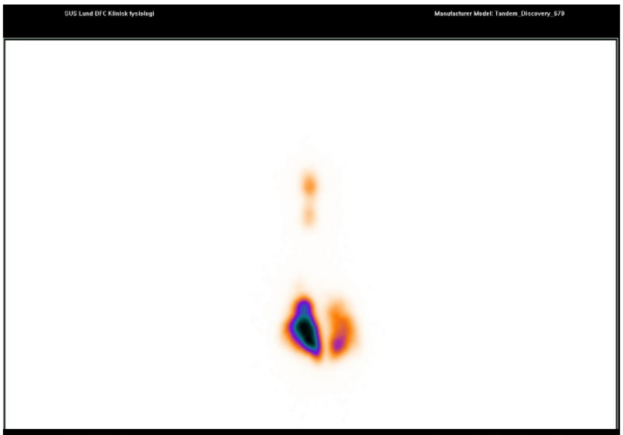
SUS Lund BFC Klinisk fysiologi
Manufacturer Model: Tandem_Discovery_670

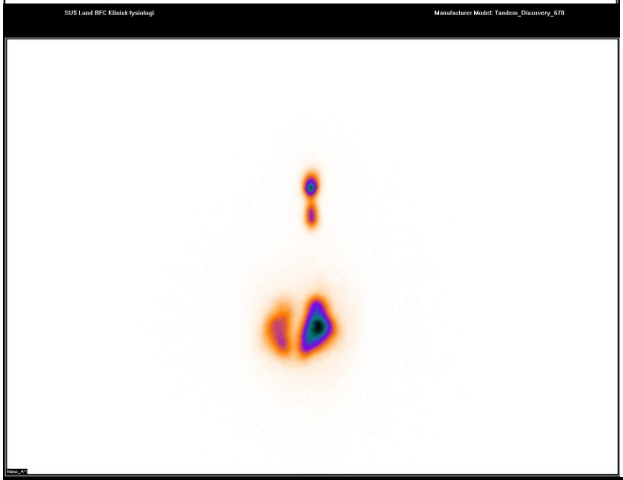
SUS Lund BFC Klinisk fysiologi
Manufacturer Model: Tandem_Discovery_670

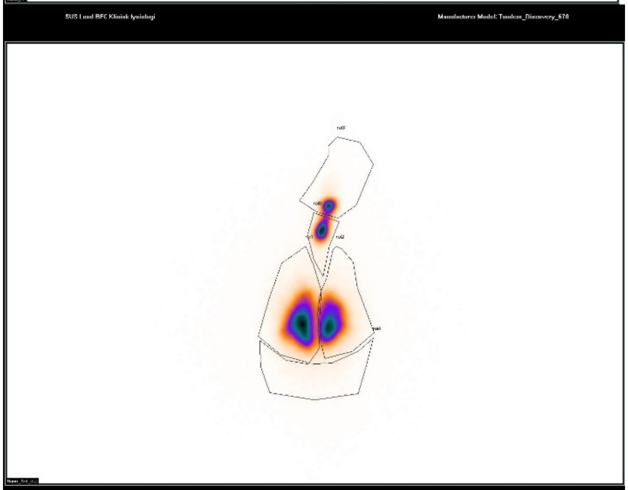
SUS Lund BFC Klinisk fysiologi
Manufacturer Model: Tandem_Discovery_670

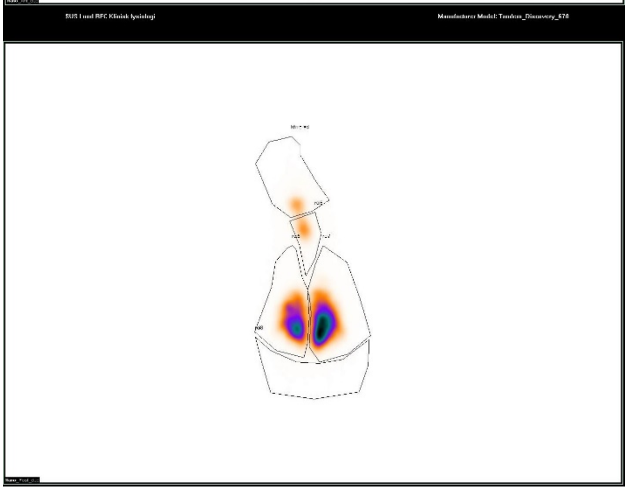
SUS Lund BFC Klinisk fysiologi
Manufacturer Model: Tandem_Discovery_670

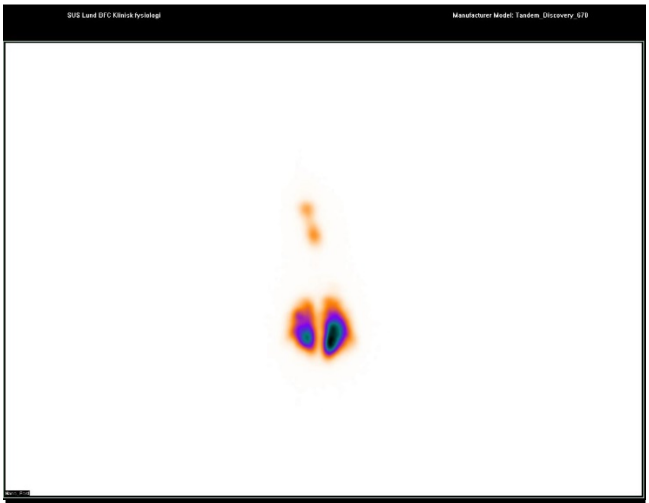
SUS Lund BFC Klinisk fysiologi
Manufacturer Model: Tandem_Discovery_670
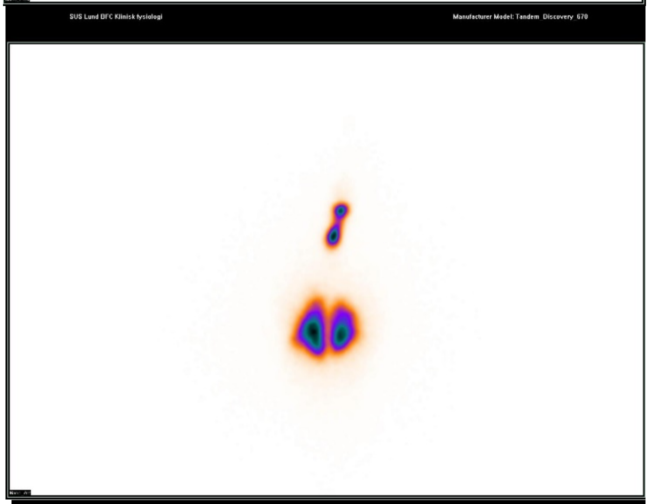
SUS Lund BFC Klinisk fysiologi
Manufacturer Model: Tandem_Discovery_670
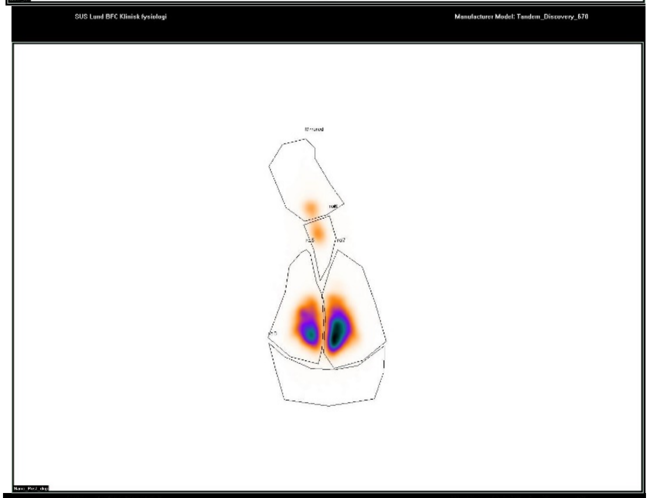
SUS Lund BFC Klinisk fysiologi
Manufacturer Model: Tandem_Discovery_670
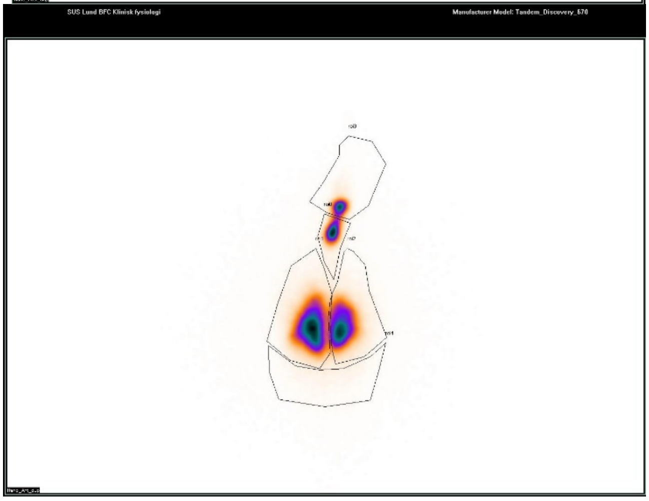
SUS Lund BFC Klinisk fysiologi
Manufacturer Model: Tandem_Discovery_670

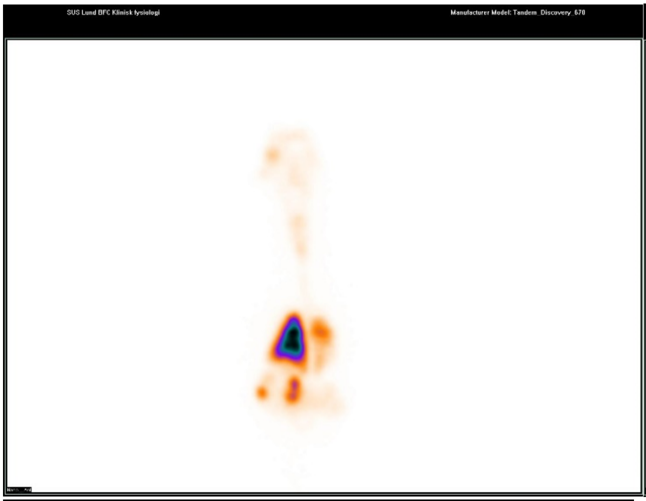
SUS Lund BFC Klinisk fysiologi
Manufacturer Model: Tandem_Discovery_670

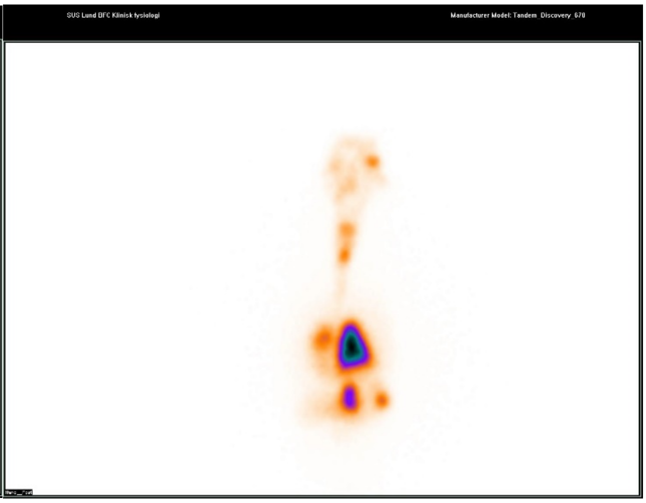
SUS Lund BFC Klinisk fysiologi
Manufacturer Model: Tandem_Discovery_670

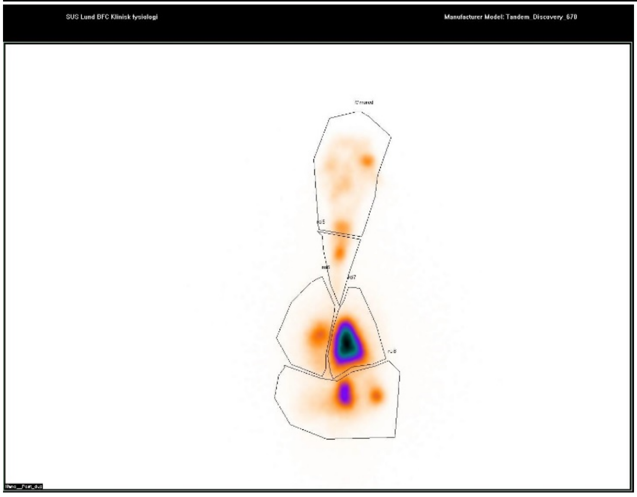
SUS Lund BFC Klinisk fysiologi
Manufacturer Model: Tandem_Discovery_670

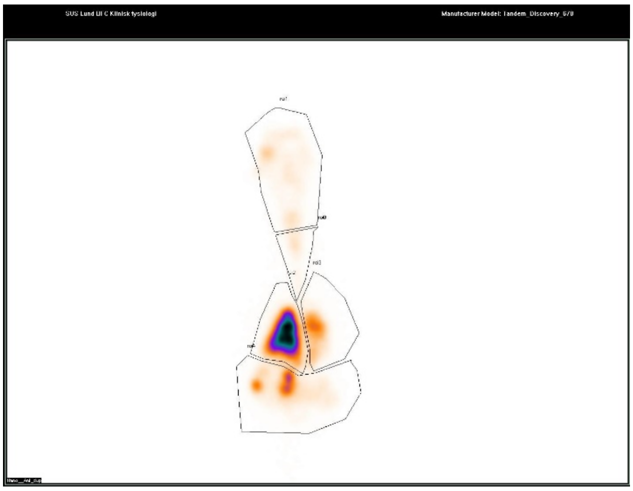
SUS Lund UFC Klinisk fysiologi
Manufacturer Model: Tandem_Discovery_670

Patient Id: 20171006549 Date & Time: 2017-10-06

SUS Lund BFC Klinisk fysiologi Manufacturer Model: Tandem_Discovery_670

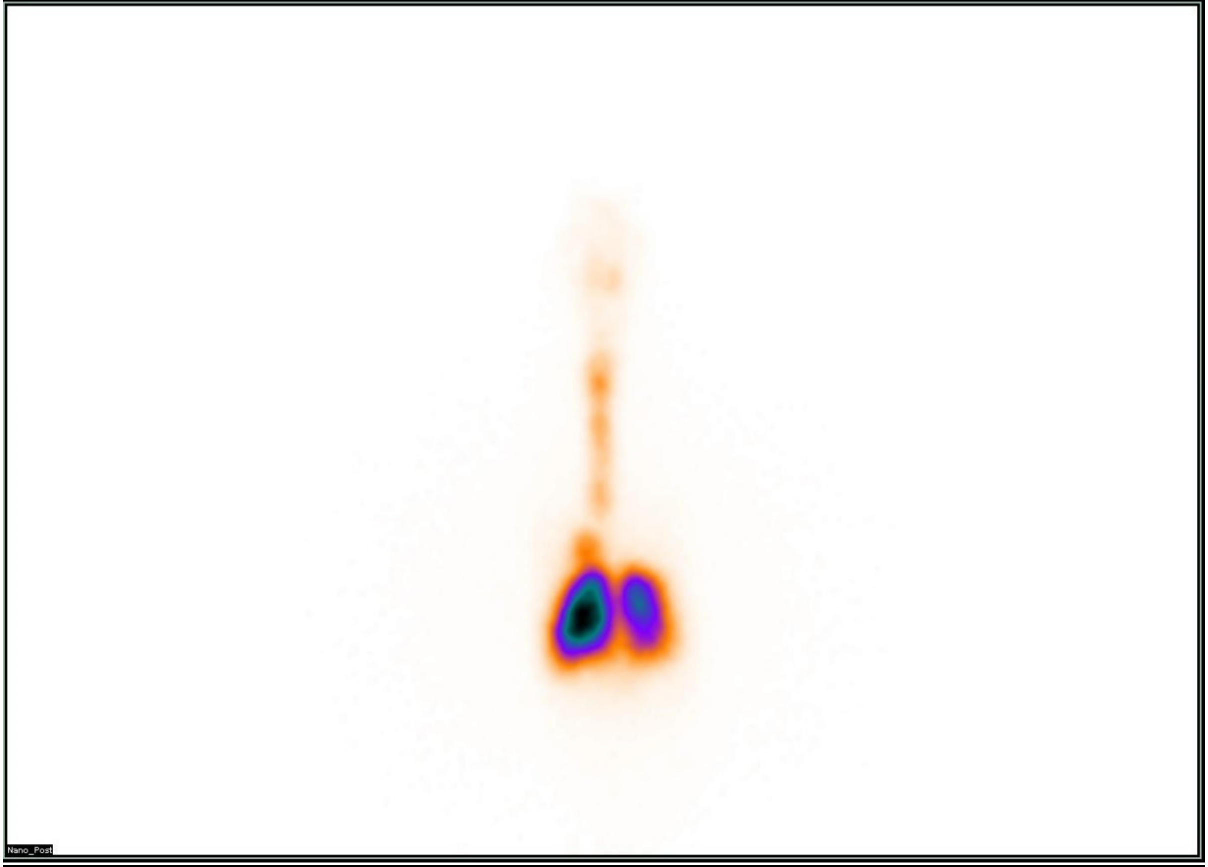

SUS Lund BFC Klinisk fysiologi Manufacturer Model: Tandem_Discovery_670

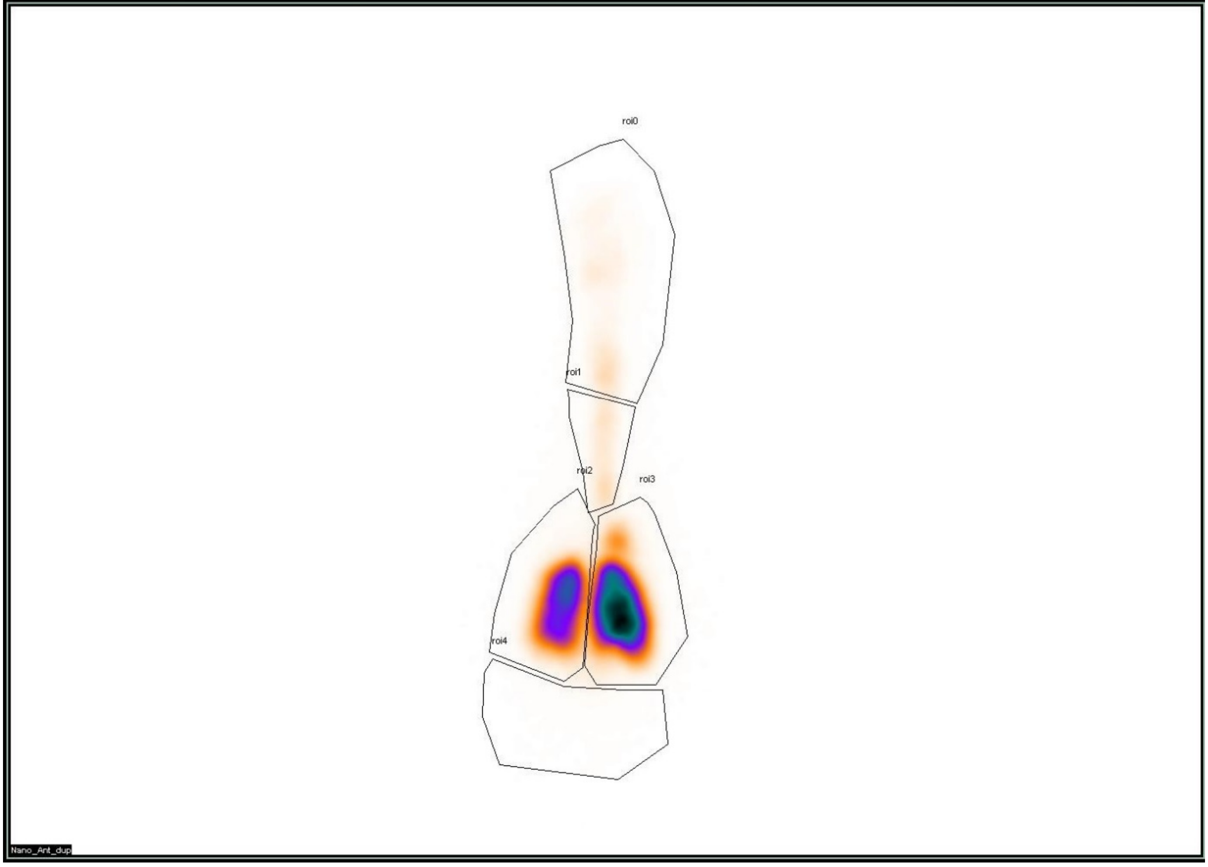

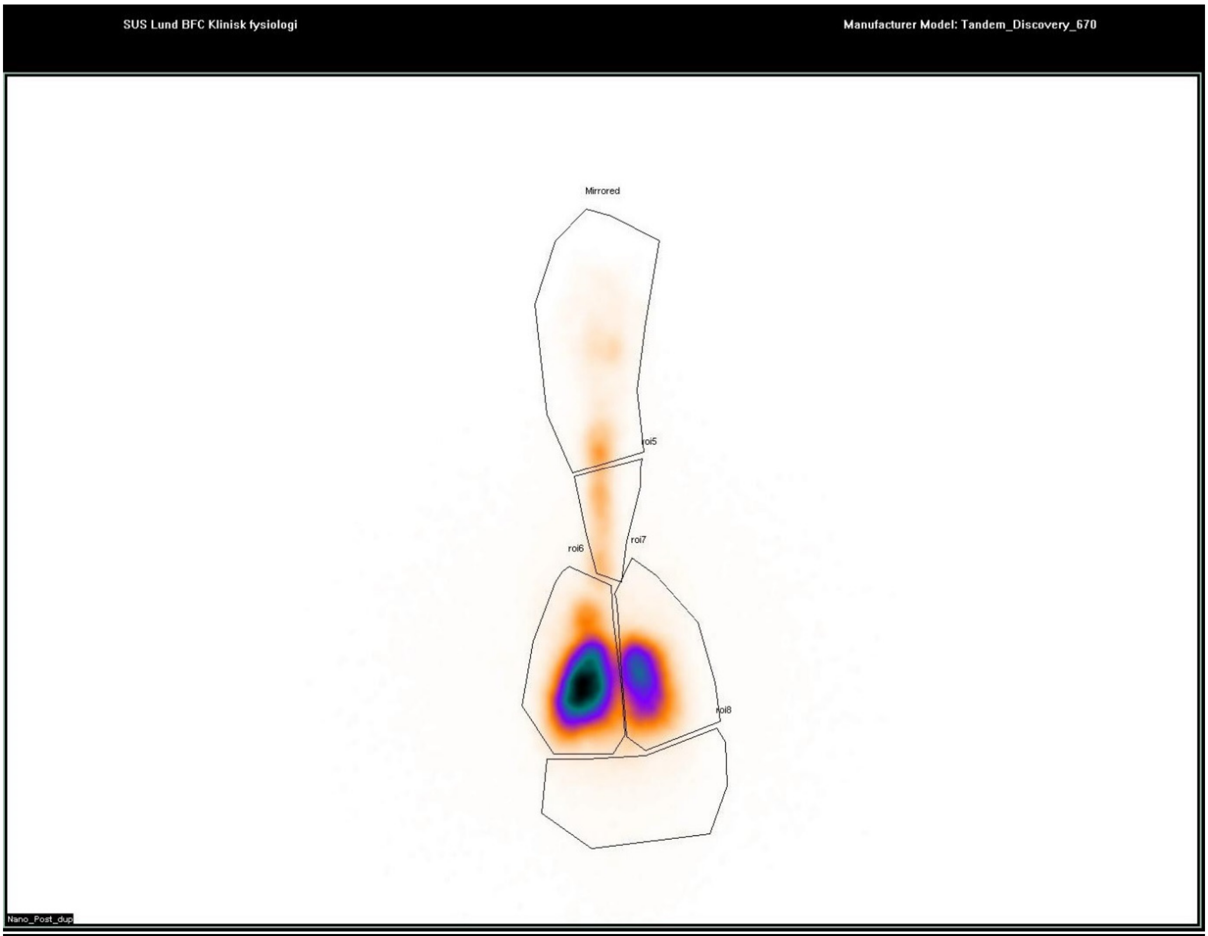
SUS Lund BFC Klinisk fysiologi
Manufacturer Model: Tandem_Discovery_670
Mirrored
roi5
roi6
roi7
roi8
Nano_Post_dup

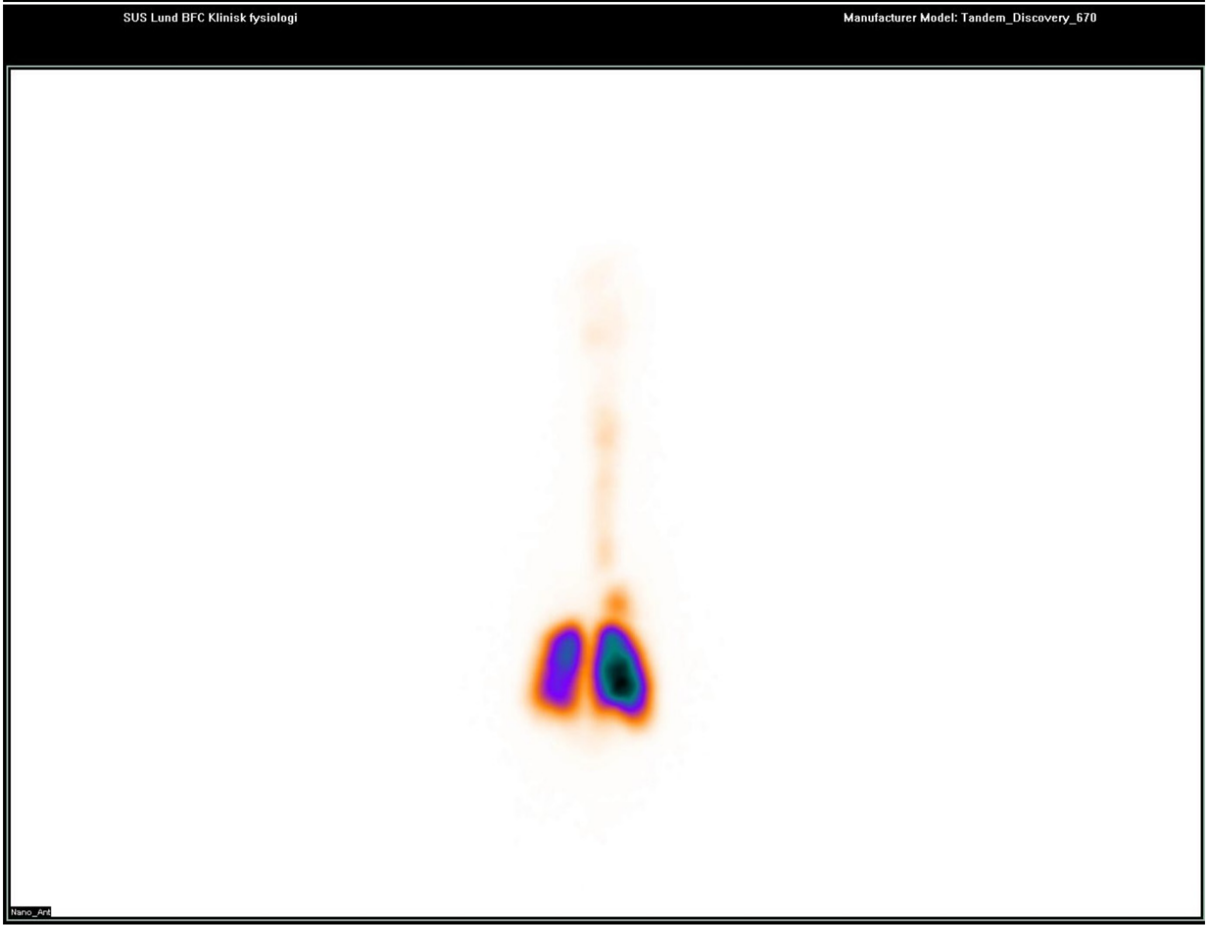
SUS Lund BFC Klinisk fysiologi
Manufacturer Model: Tandem_Discovery_670
Nano_Ant

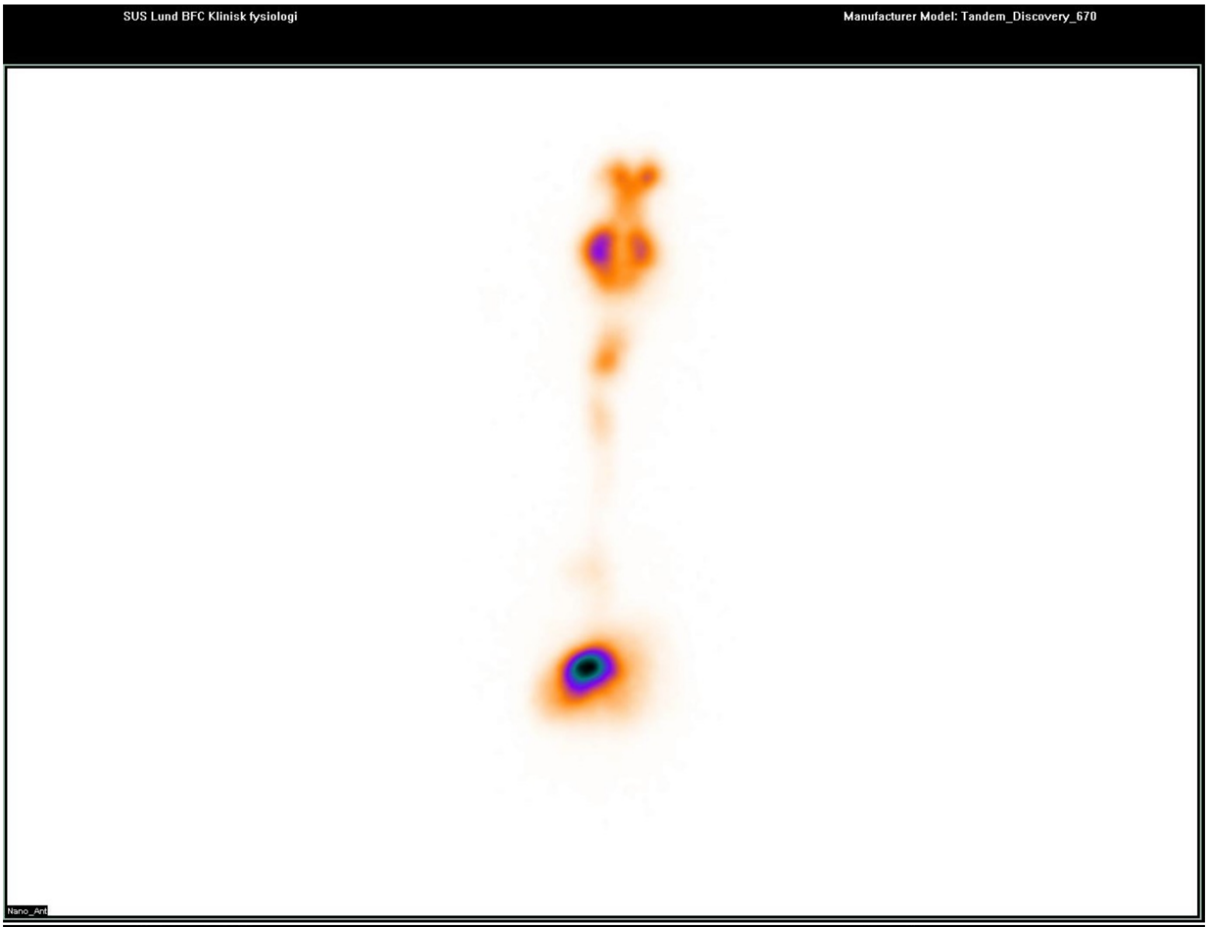
SUS Lund BFC Klinisk fysiologi
Manufacturer Model: Tandem_Discovery_670
Nano_Ant

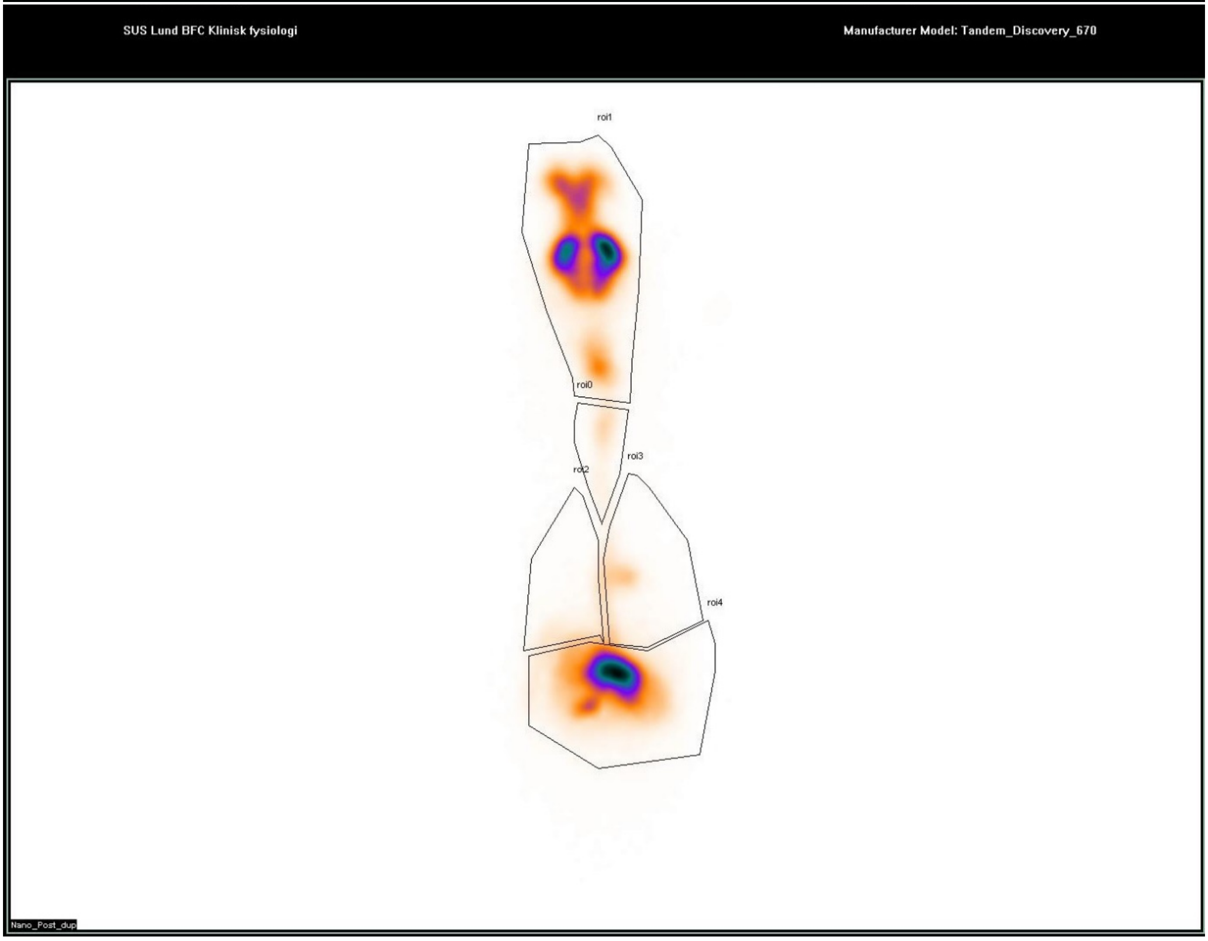
SUS Lund BFC Klinisk fysiologi
Manufacturer Model: Tandem_Discovery_670
roi1
roi0
roi2
roi3
roi4
Nano_Post_dup

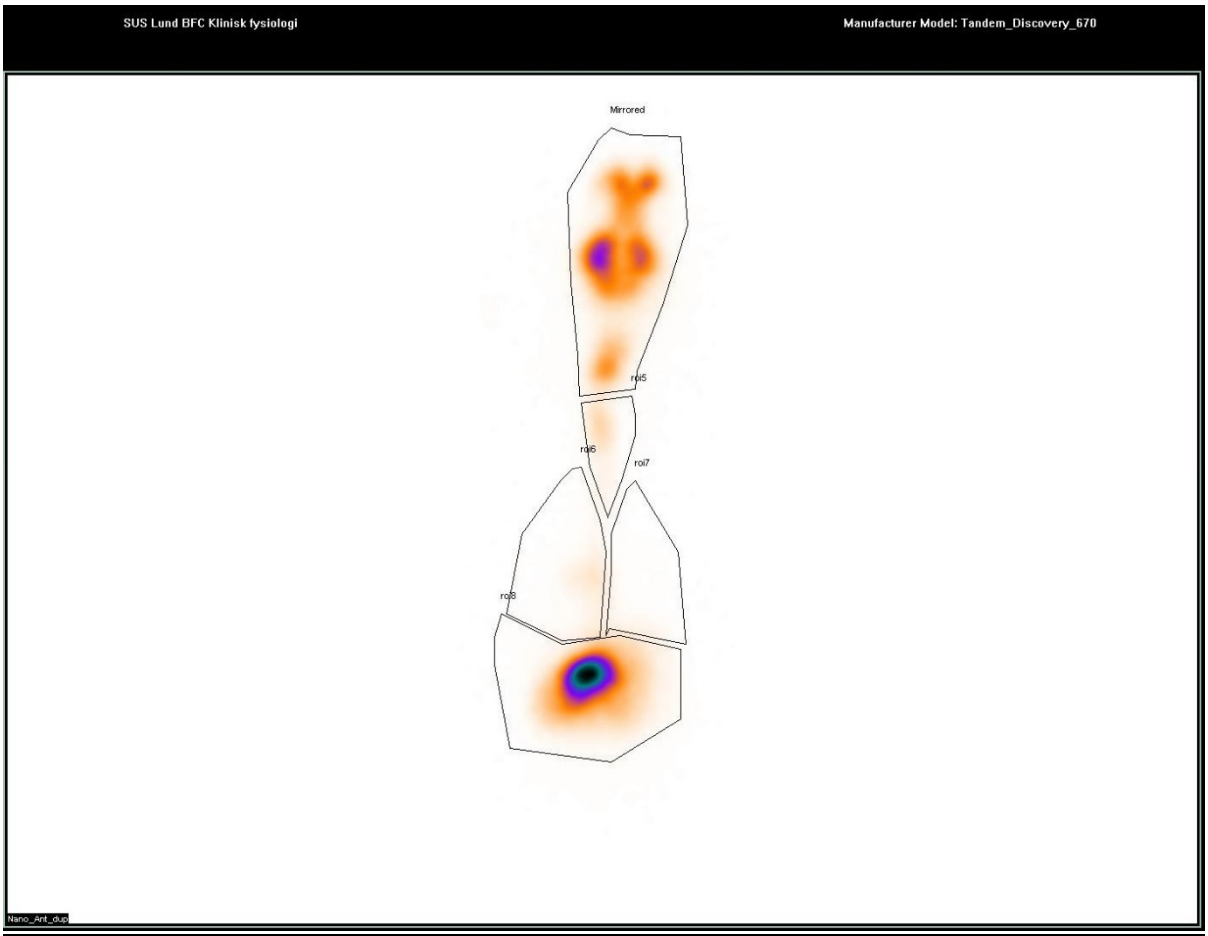
SUS Lund BFC Klinisk fysiologi
Manufacturer Model: Tandem_Discovery_670
Mirrored
roi5
roi6
roi7
roi8
Nano_Ant_dup

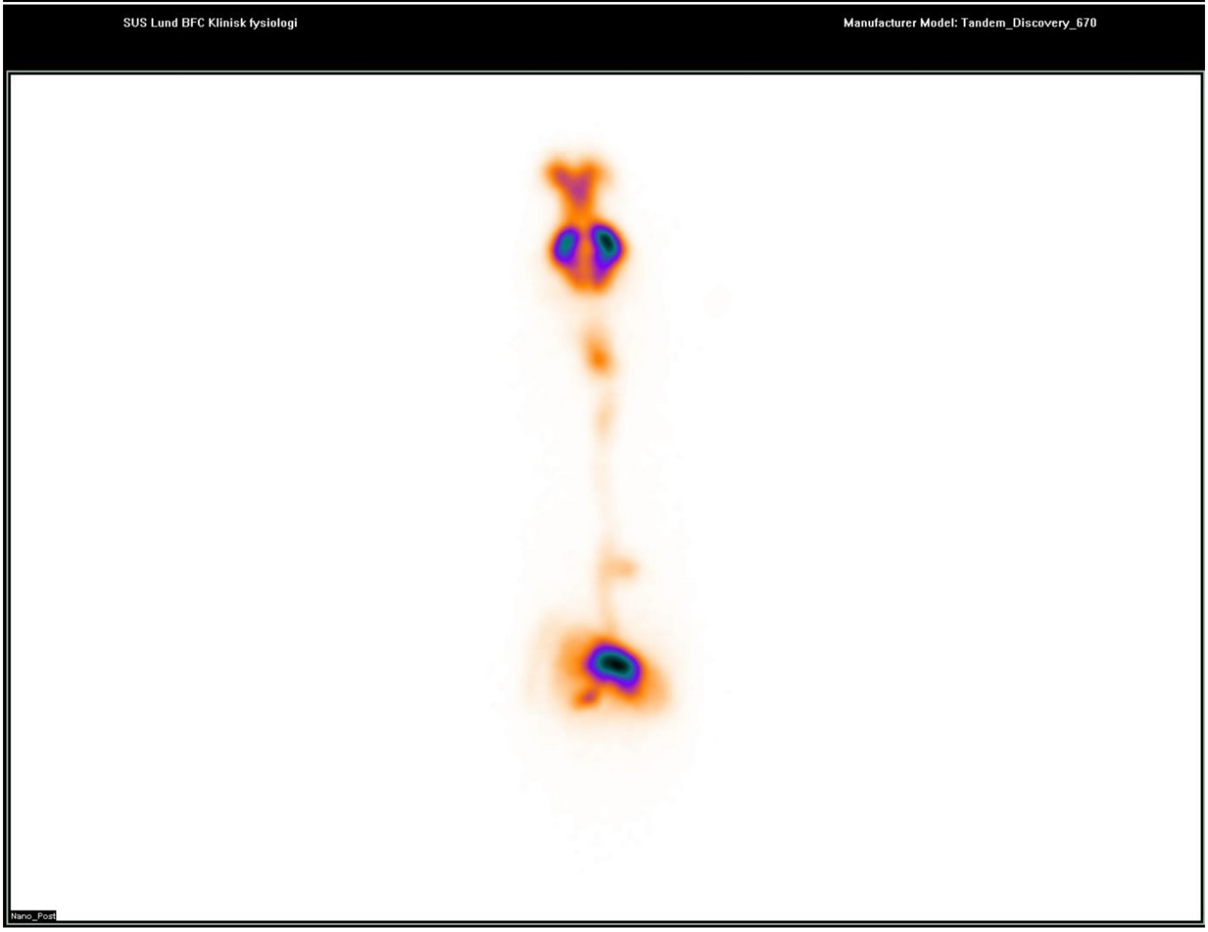
SUS Lund BFC Klinisk fysiologi
Manufacturer Model: Tandem_Discovery_670
Nano_Post

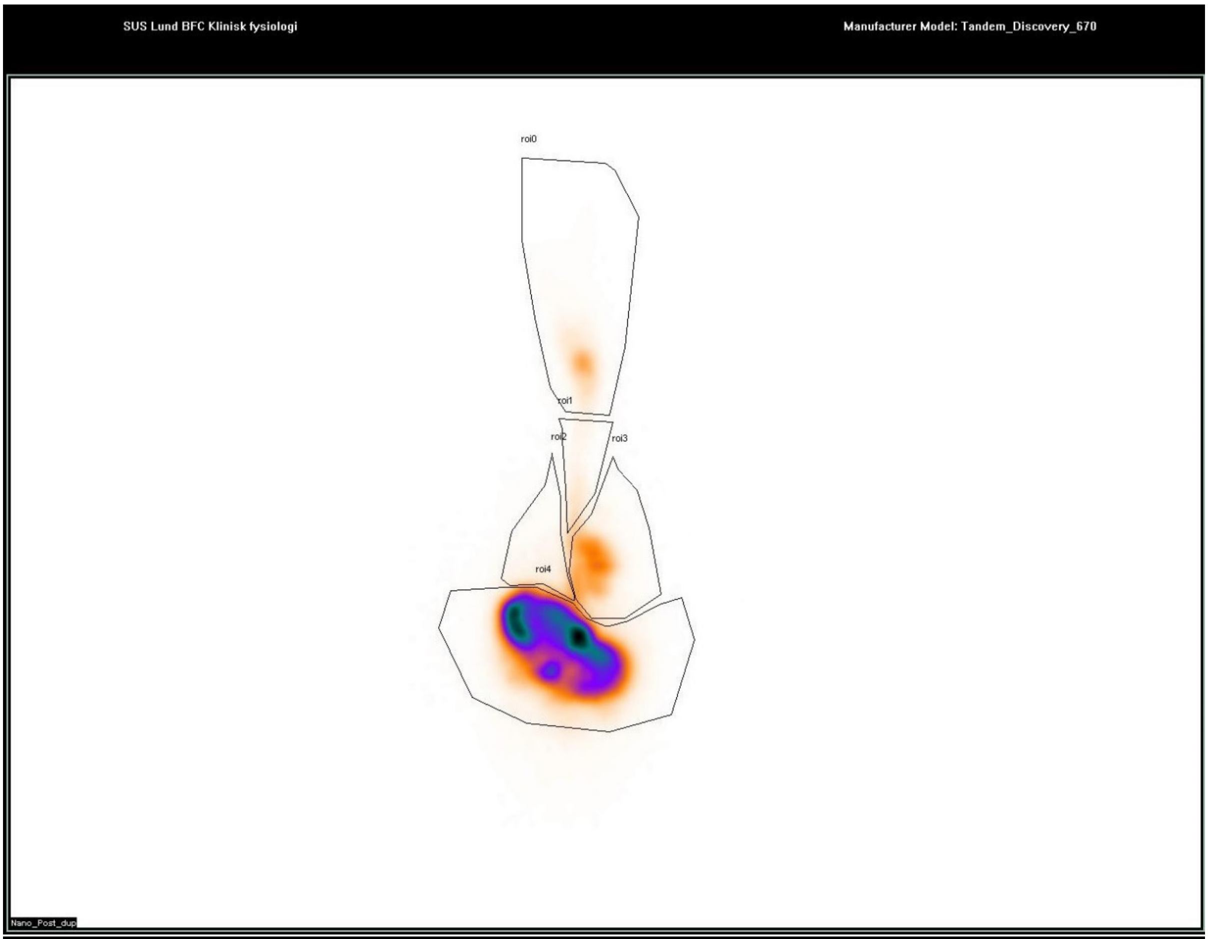
SUS Lund BFC Klinisk fysiologi
Manufacturer Model: Tandem_Discovery_670
roi0
roi1
roi2
roi3
roi4
Nano_Post_dup

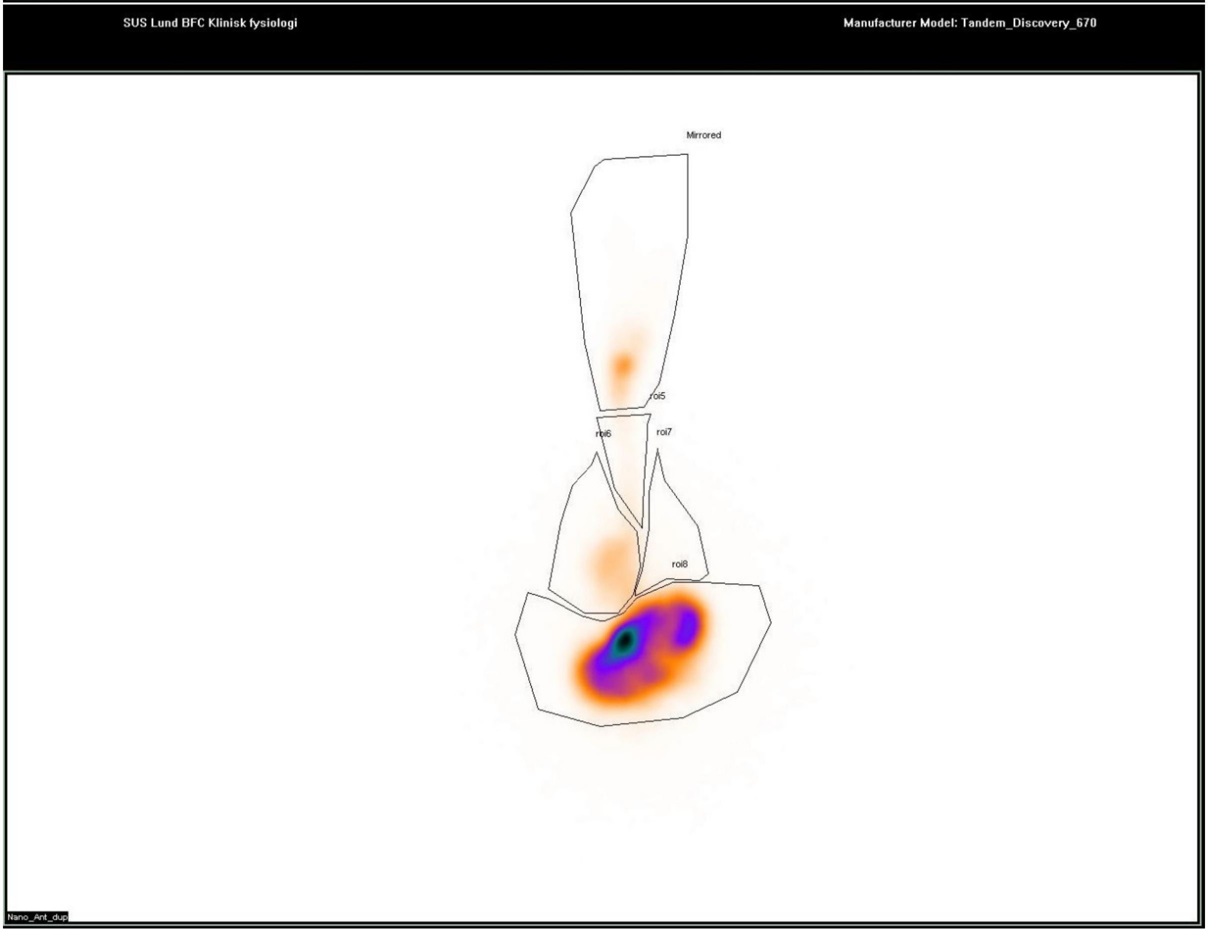
SUS Lund BFC Klinisk fysiologi
Manufacturer Model: Tandem_Discovery_670
Mirrored
roi5
roi6
roi7
roi8
Nano_Ant_dup

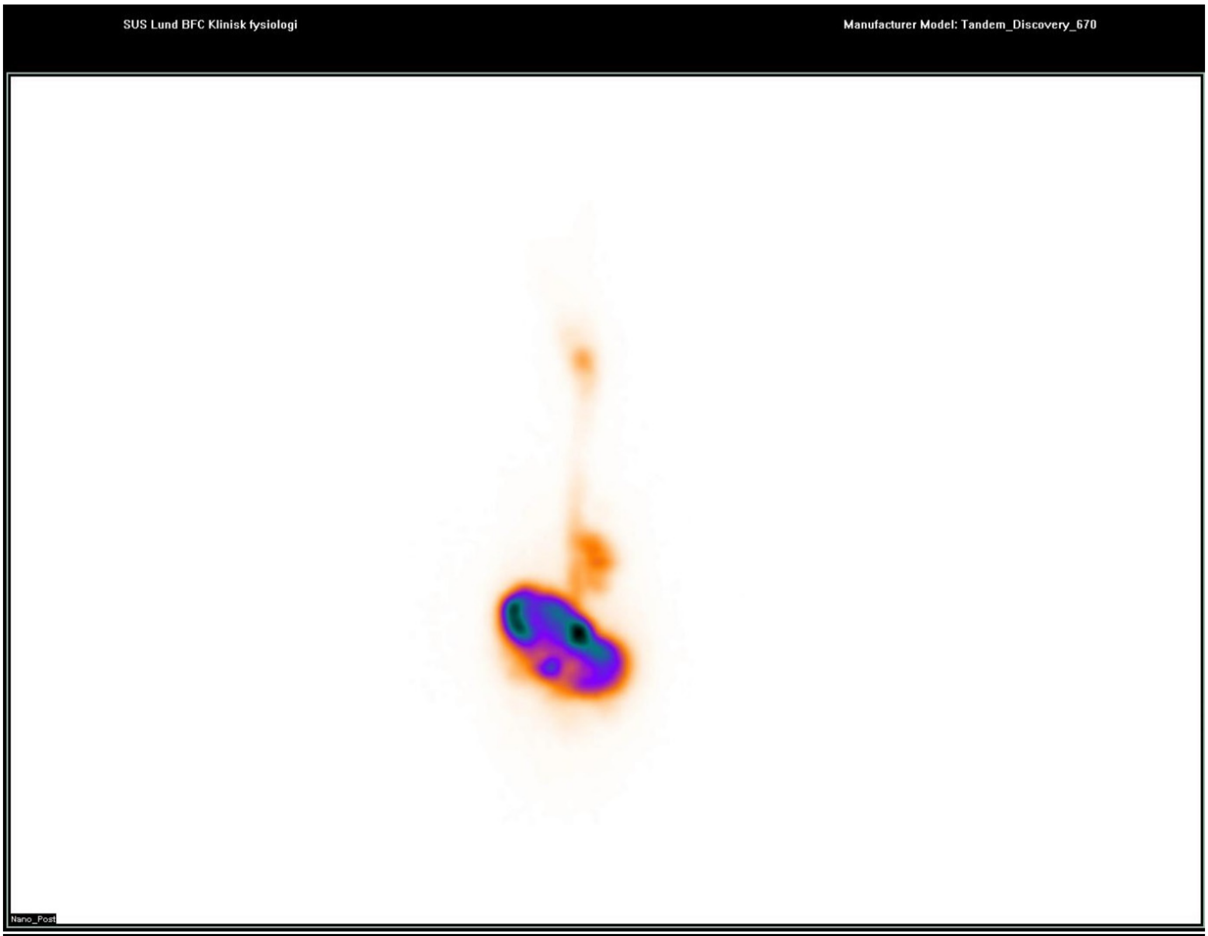
SUS Lund BFC Klinisk fysiologi
Manufacturer Model: Tandem_Discovery_670
Nano_Post

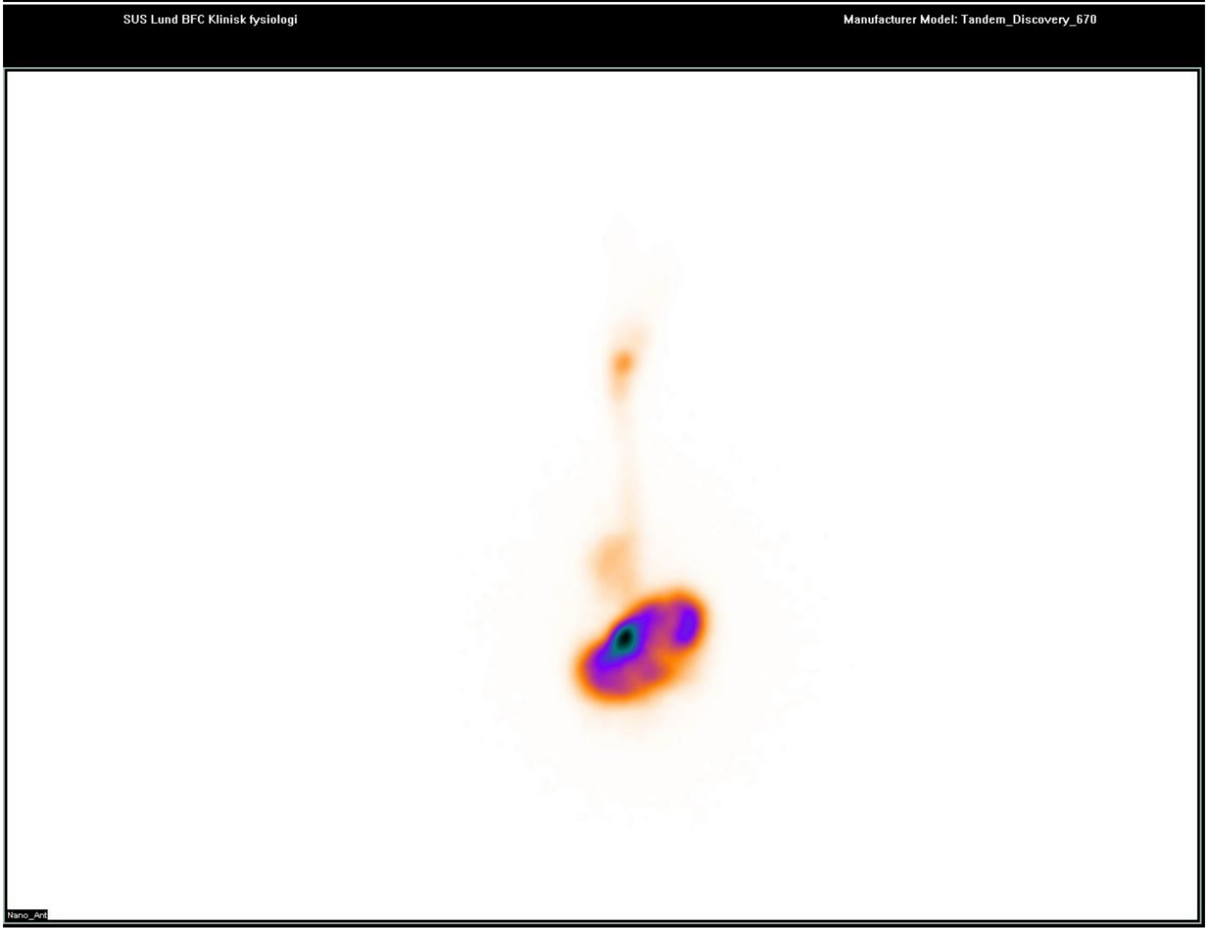
SUS Lund BFC Klinisk fysiologi
Manufacturer Model: Tandem_Discovery_670
Nano_Ant

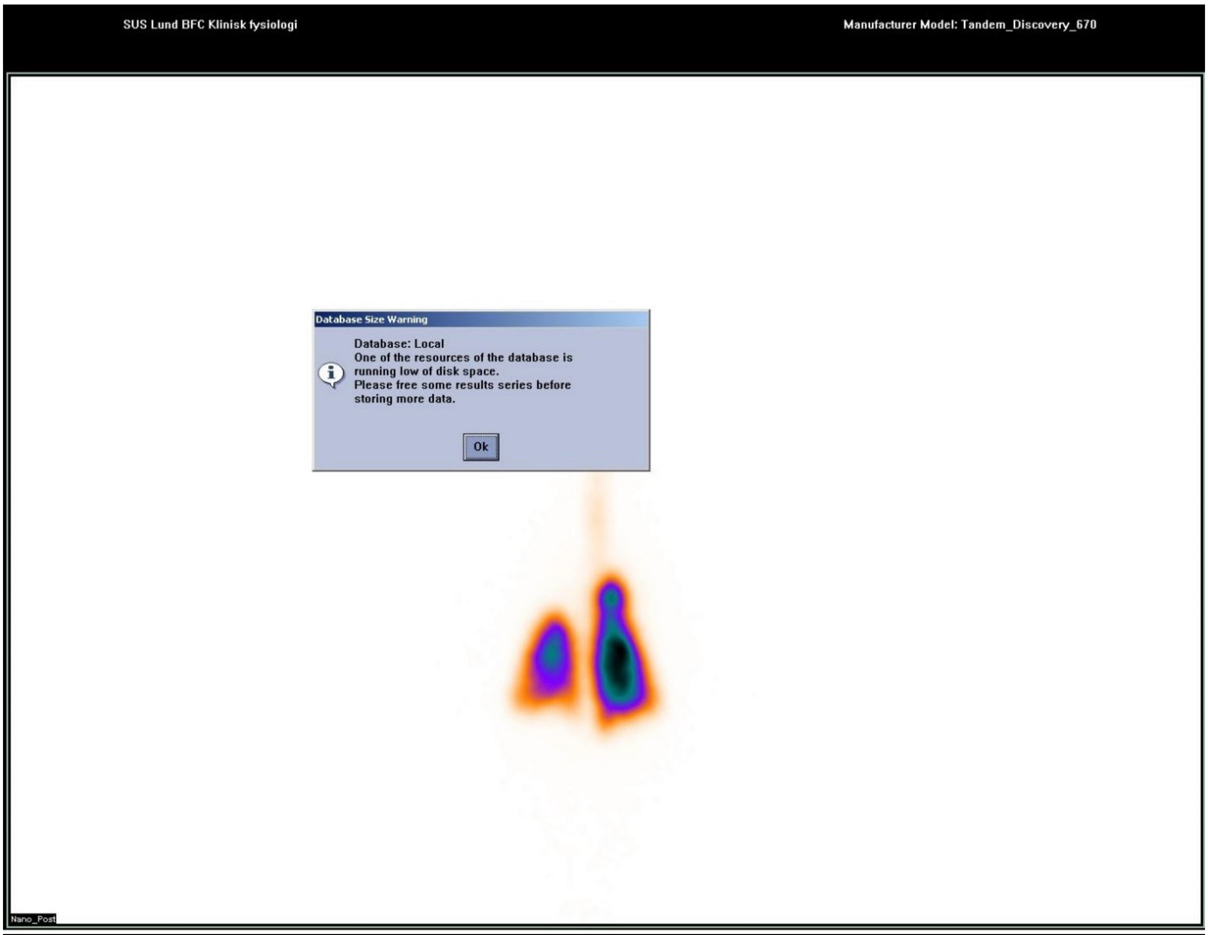
SUS Lund BFC Klinisk fysiologi
Manufacturer Model: Tandem_Discovery_670
Database Size Warning
Database: Local
One of the resources of the database is
running low of disk space.
Please free some results series before
storing more data.
Ok
Nano_Post

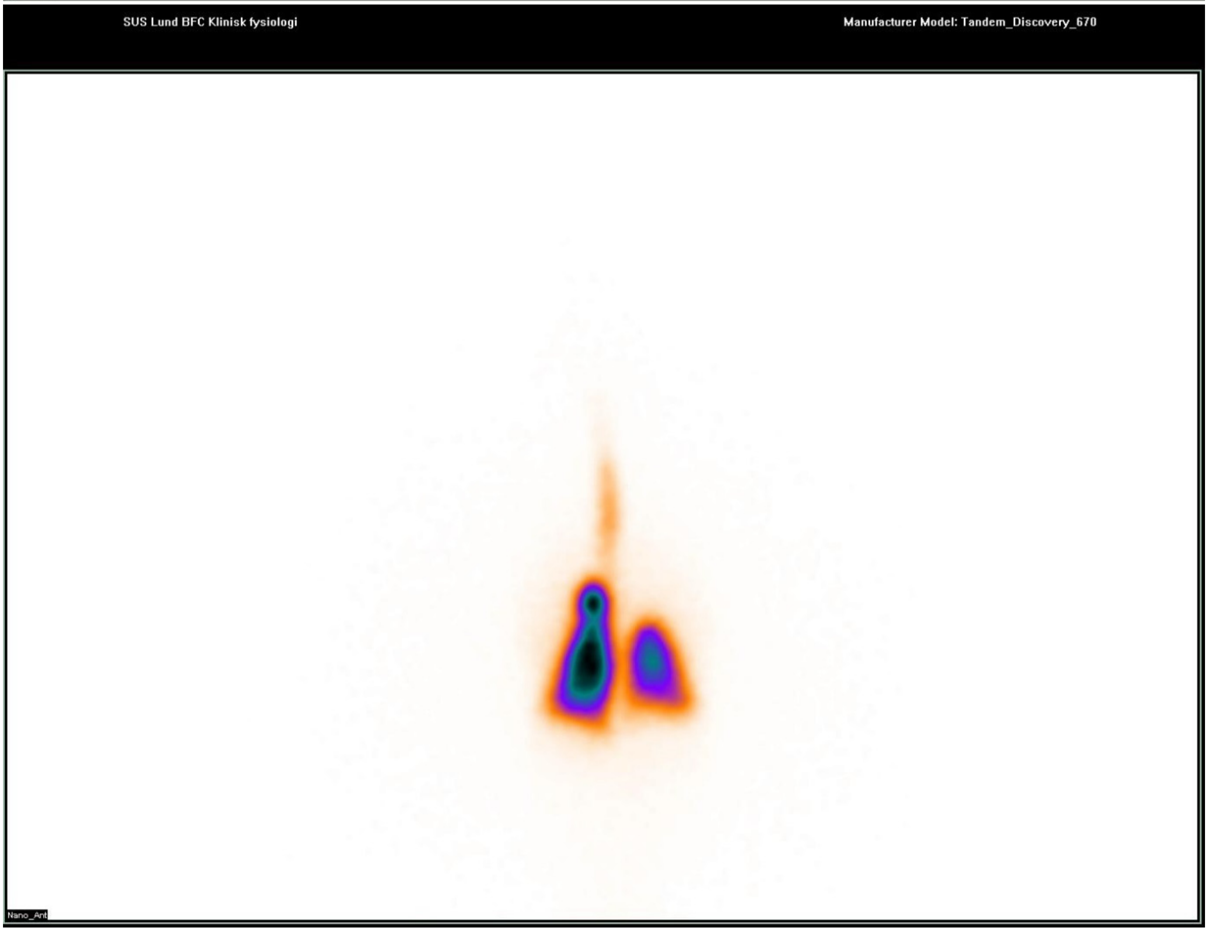
SUS Lund BFC Klinisk fysiologi
Manufacturer Model: Tandem_Discovery_670
Nano_Ant

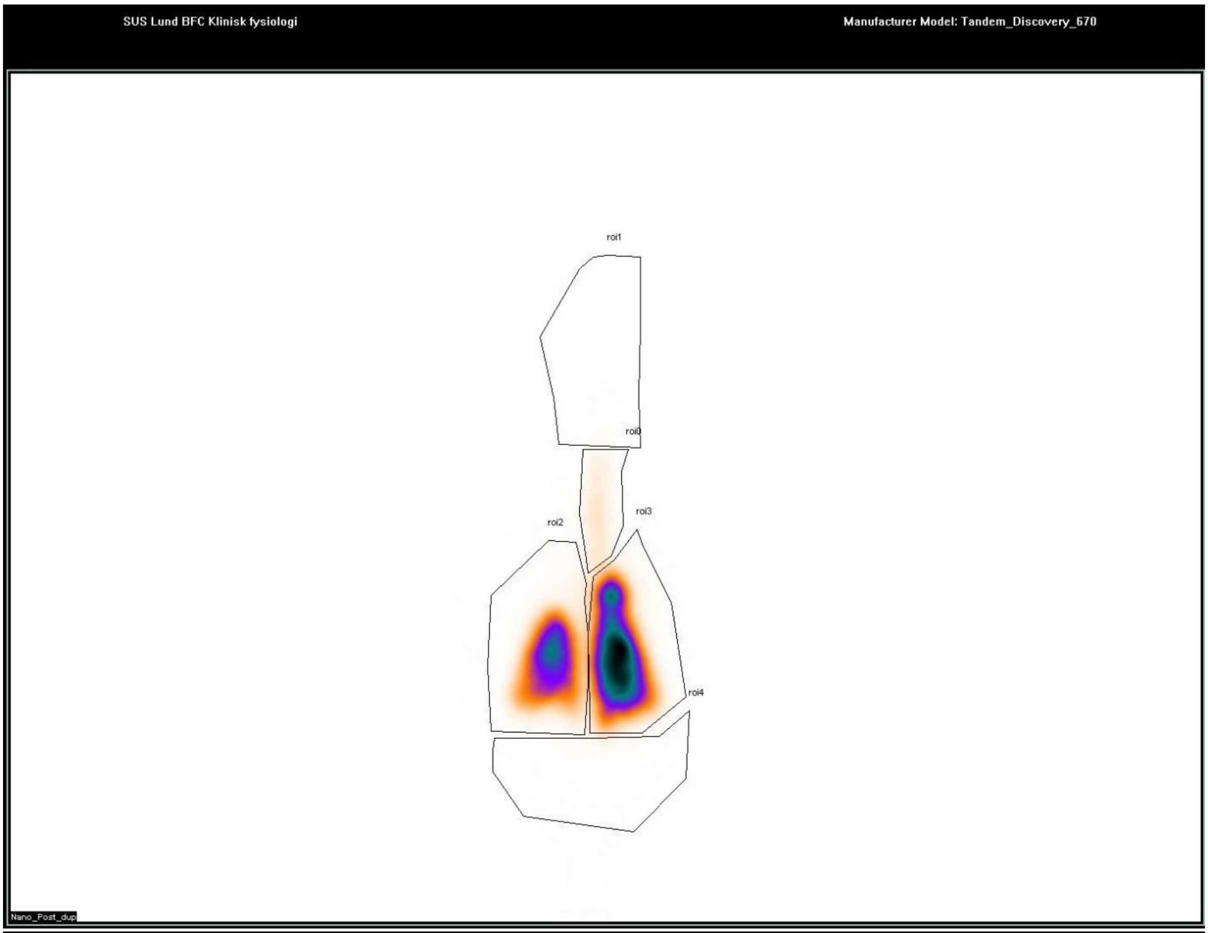
SUS Lund BFC Klinisk fysiologi
Manufacturer Model: Tandem_Discovery_670
roi1
roi0
roi2
roi3
roi4
Nano_Post_dup

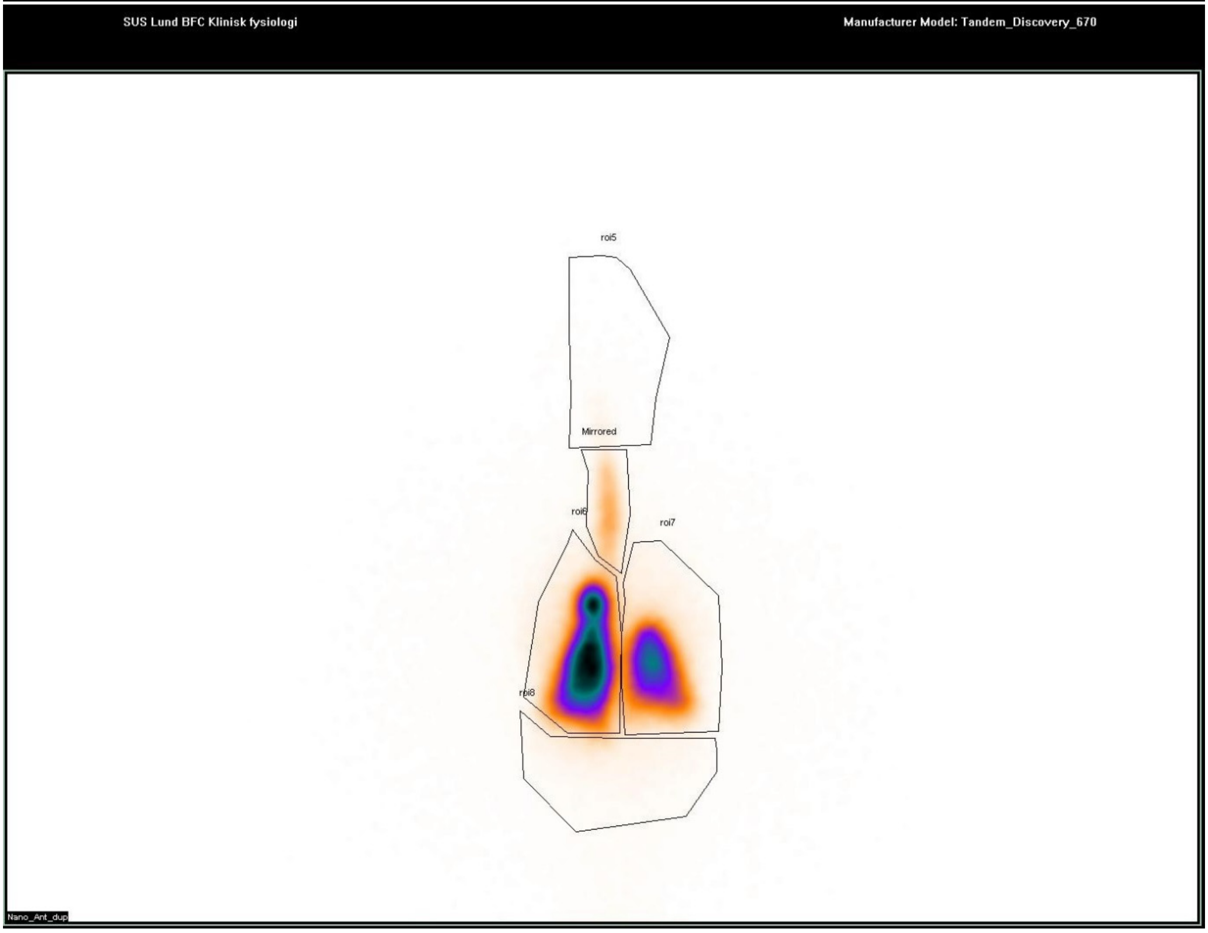
SUS Lund BFC Klinisk fysiologi
Manufacturer Model: Tandem_Discovery_670
roi5
Mirrored
roi6
roi7
roi8
Nano_Ant_dup

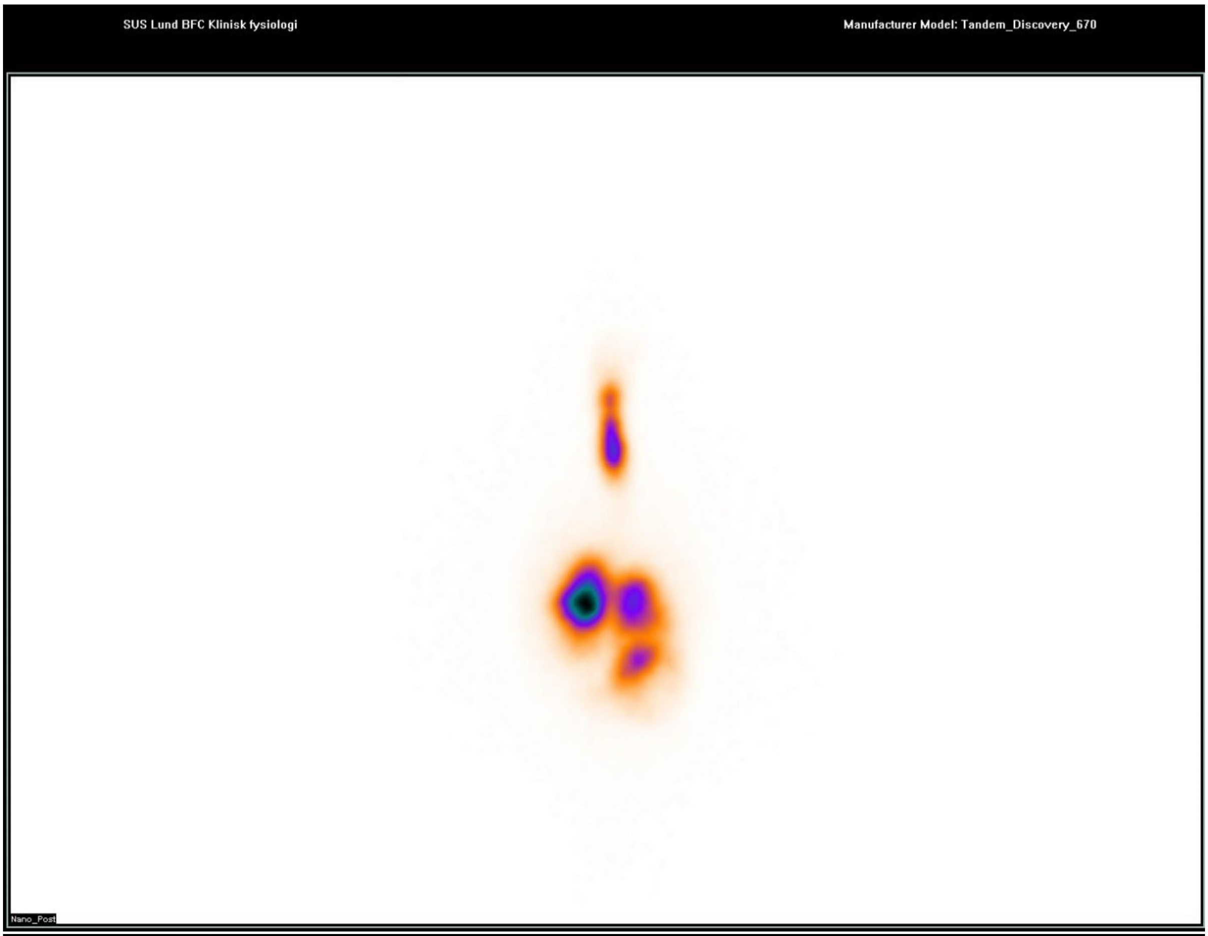
SUS Lund BFC Klinisk fysiologi
Manufacturer Model: Tandem_Discovery_670
Nano_Post

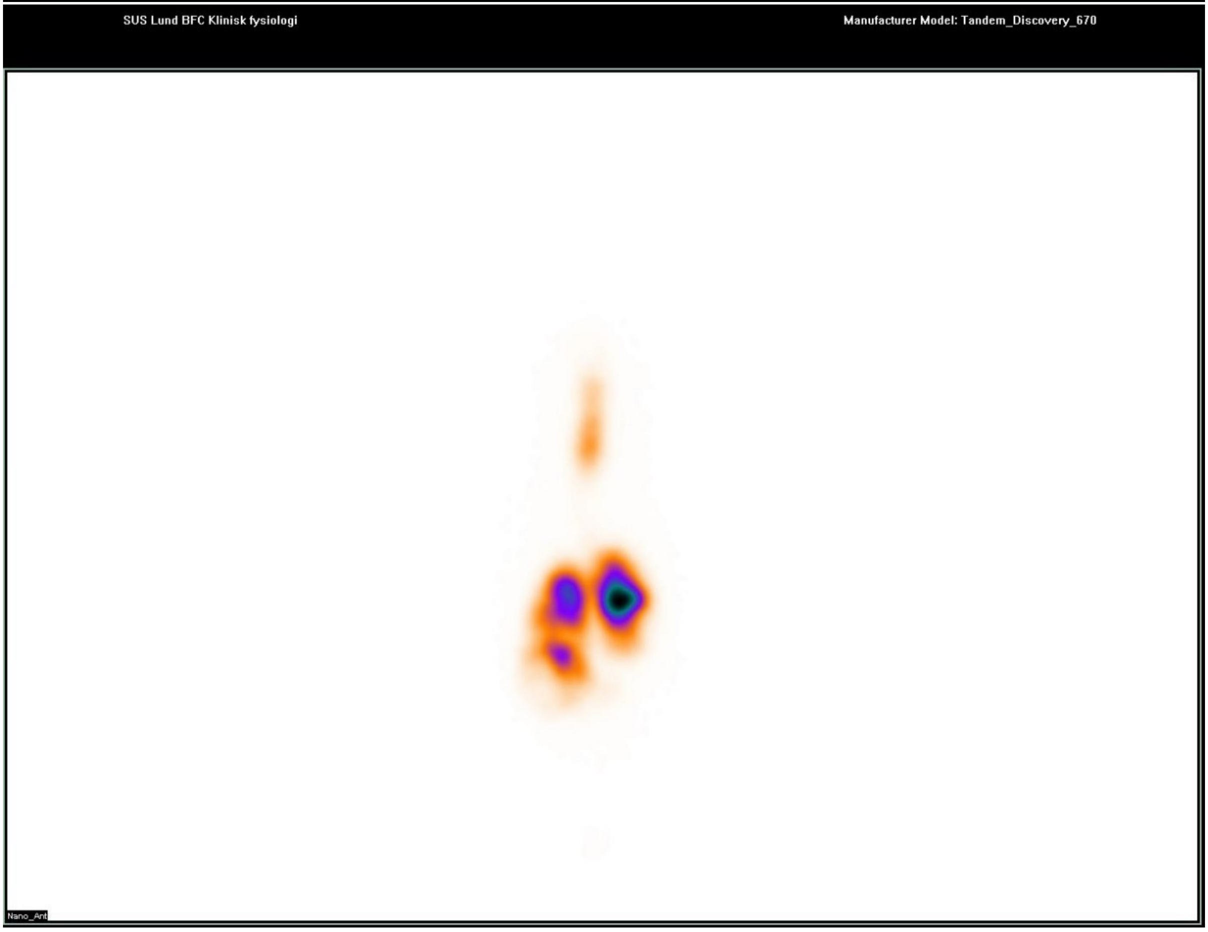
SUS Lund BFC Klinisk fysiologi
Manufacturer Model: Tandem_Discovery_670
Nano_Ant

SUS Lund BFC Klinisk fysiologi

Manufacturer Model: Tandem_Discovery_670

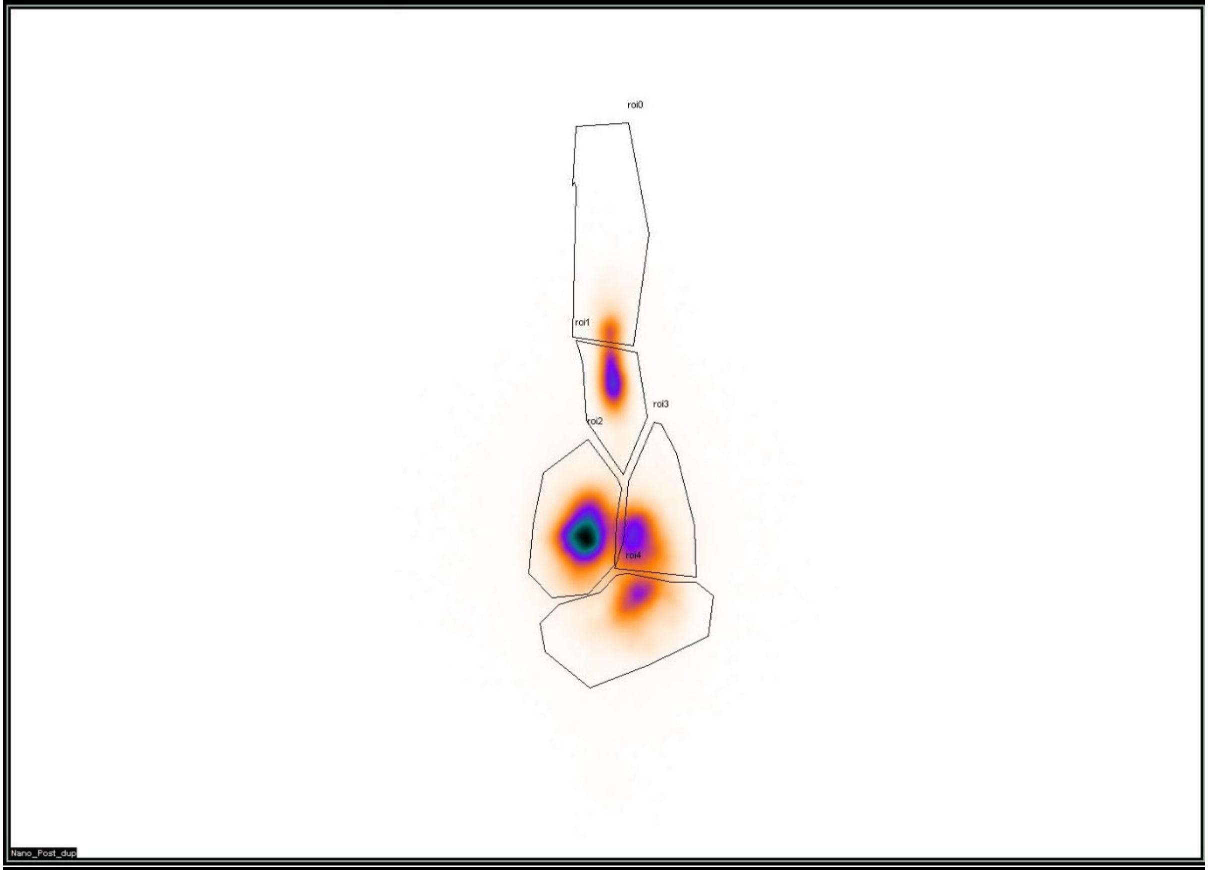

SUS Lund BFC Klinisk fysiologi

Manufacturer Model: Tandem_Discovery_670

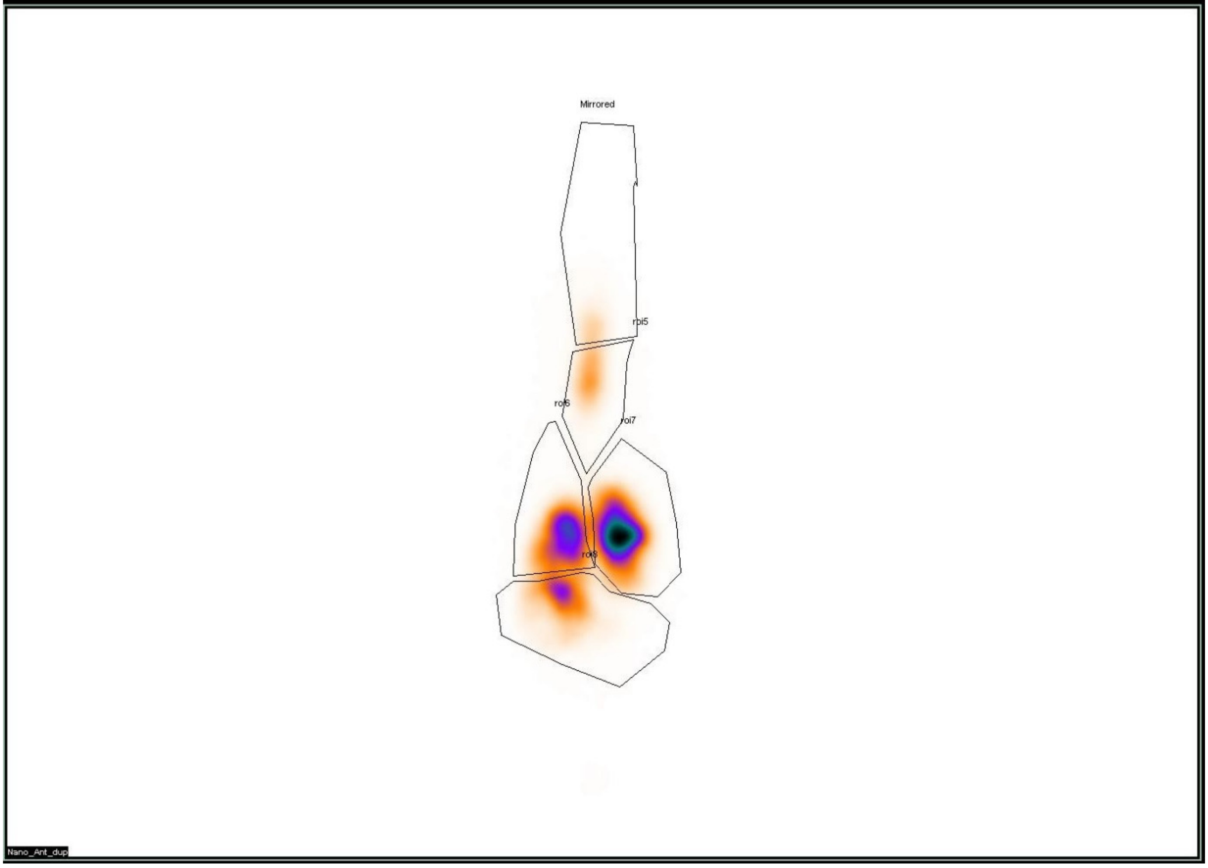

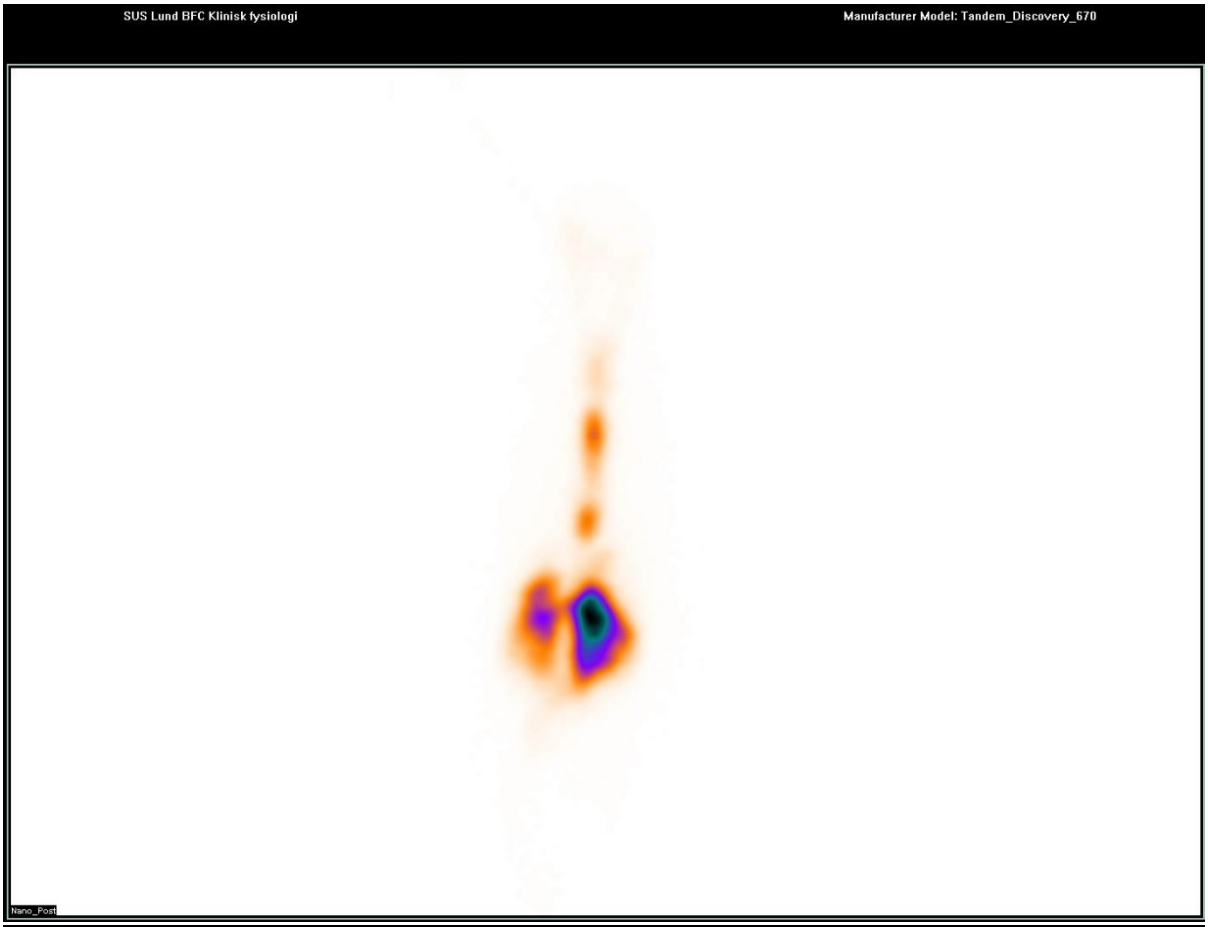
SUS Lund BFC Klinisk fysiologi
Manufacturer Model: Tandem_Discovery_670
Nano_Post

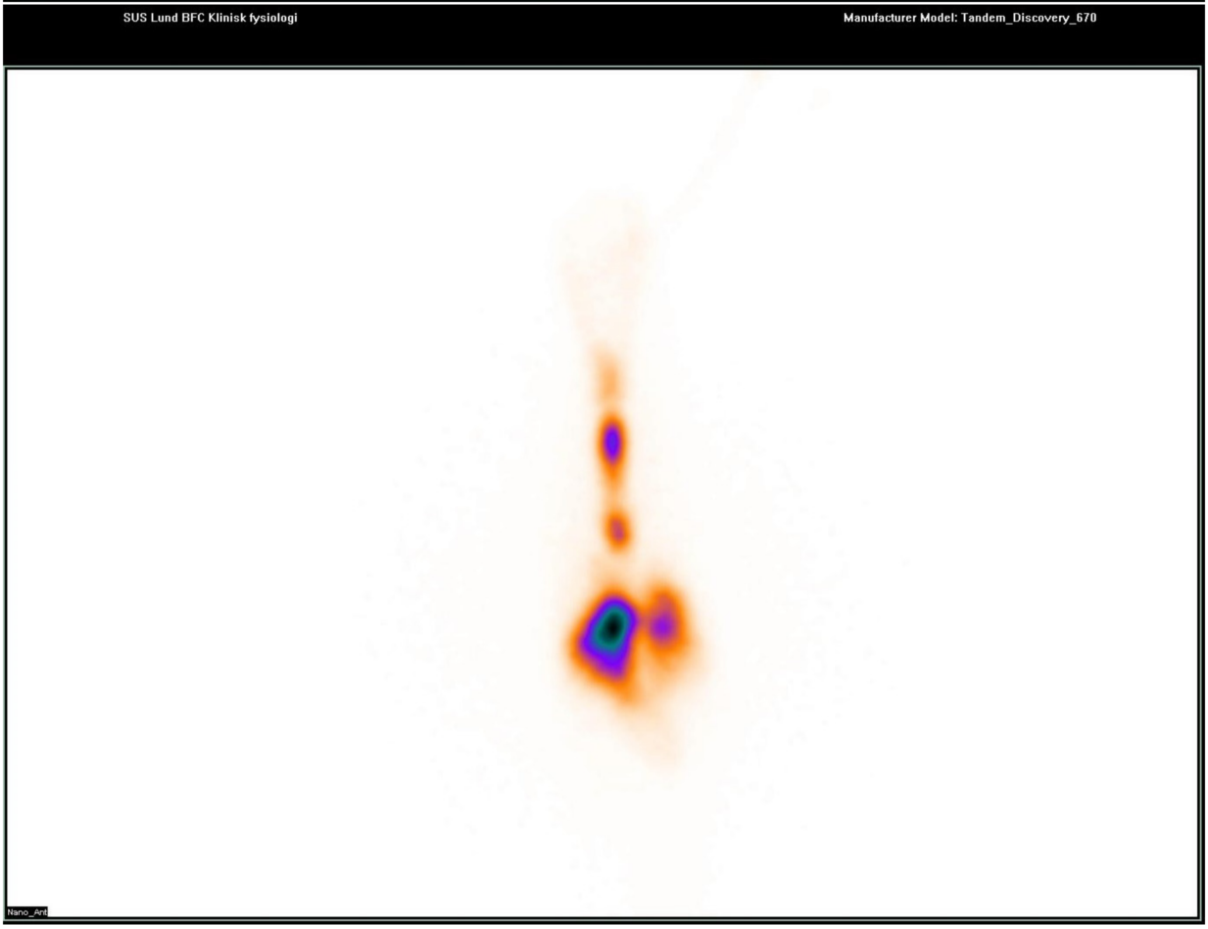
SUS Lund BFC Klinisk fysiologi
Manufacturer Model: Tandem_Discovery_670
Nano_Ant

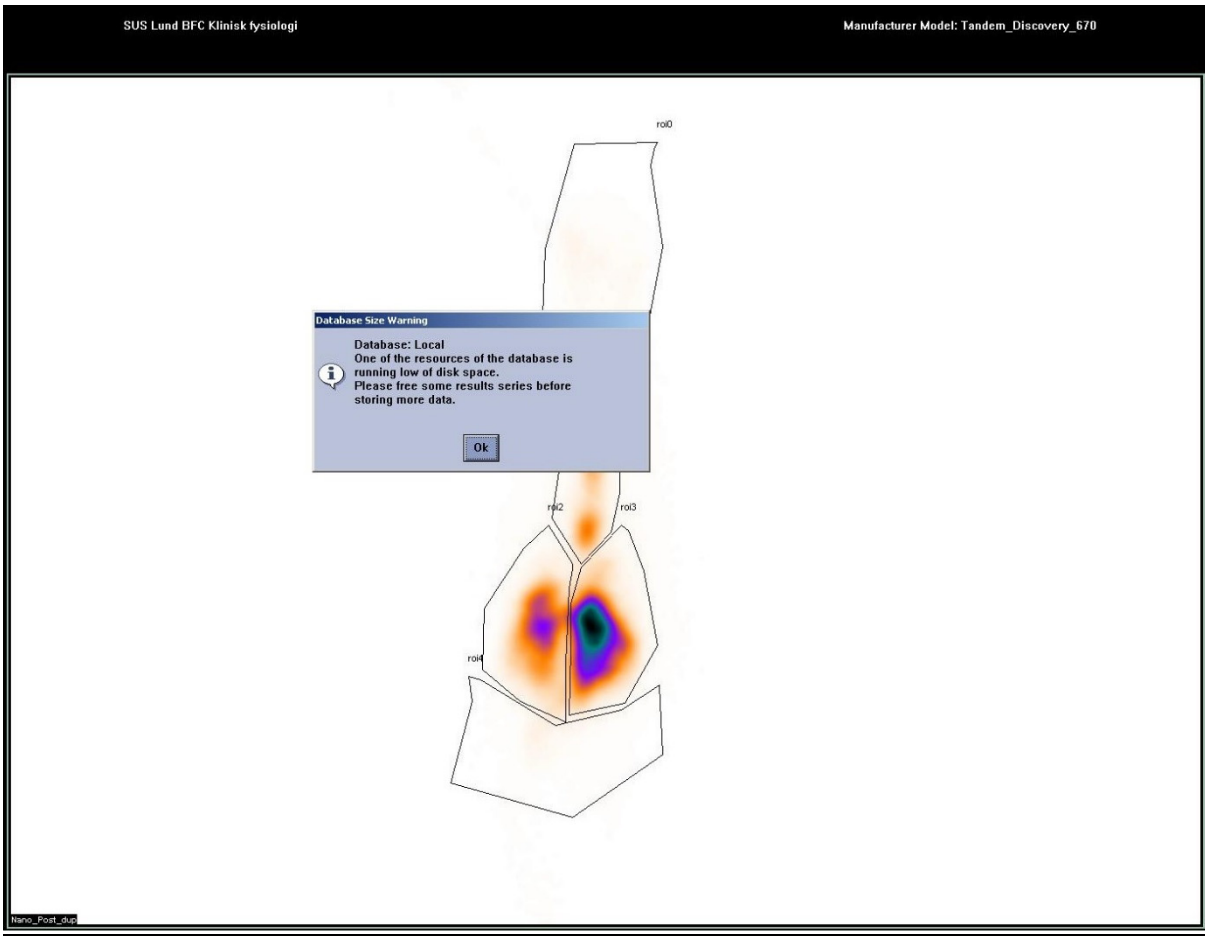
SUS Lund BFC Klinisk fysiologi
Manufacturer Model: Tandem_Discovery_670
roi0
Database Size Warning
Database: Local
One of the resources of the database is running low of disk space.
Please free some results series before storing more data.
Ok
roi2
roi3
roi4
Nano_Post_dup

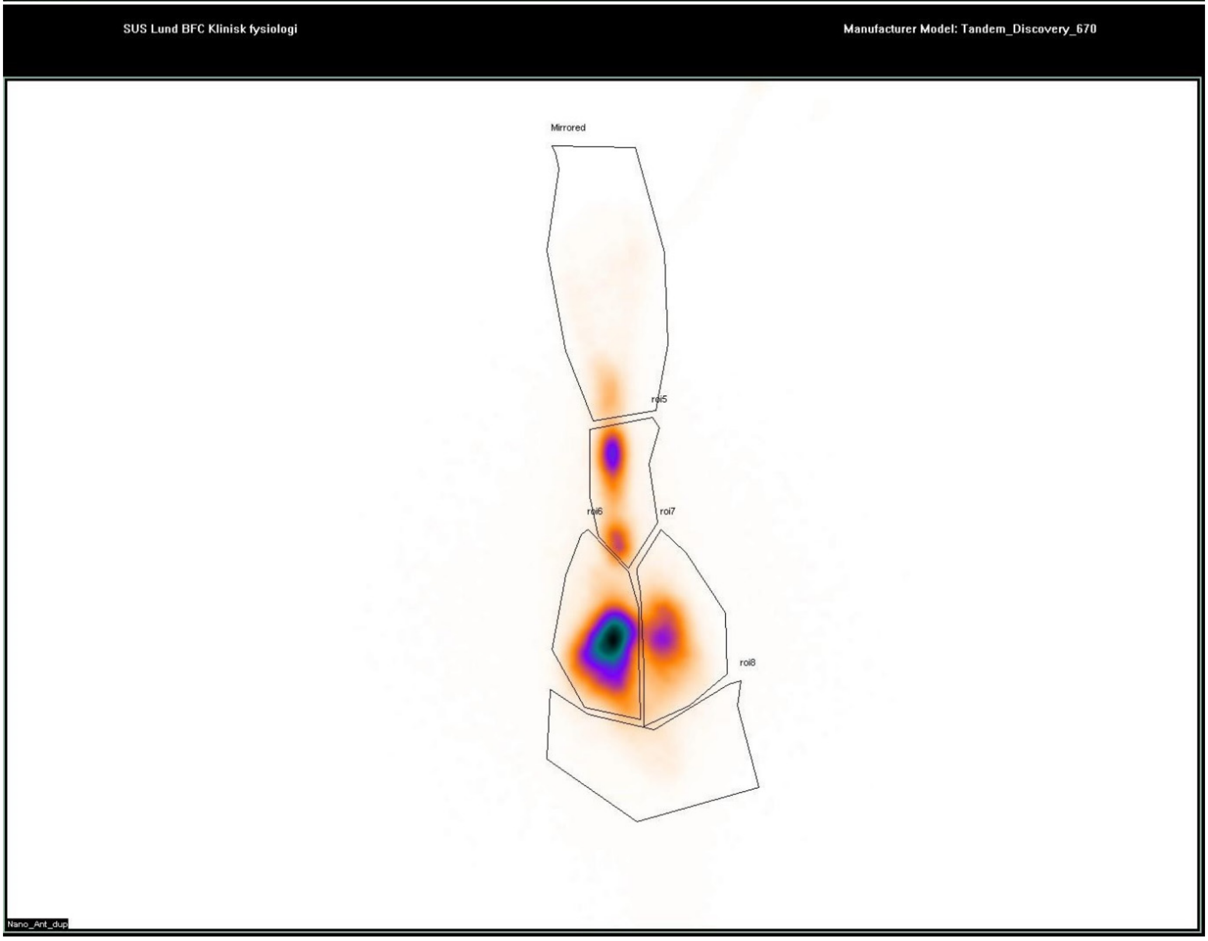
SUS Lund BFC Klinisk fysiologi
Manufacturer Model: Tandem_Discovery_670
Mirrored
roi5
roi6
roi7
roi8
Nano_Ant_dup

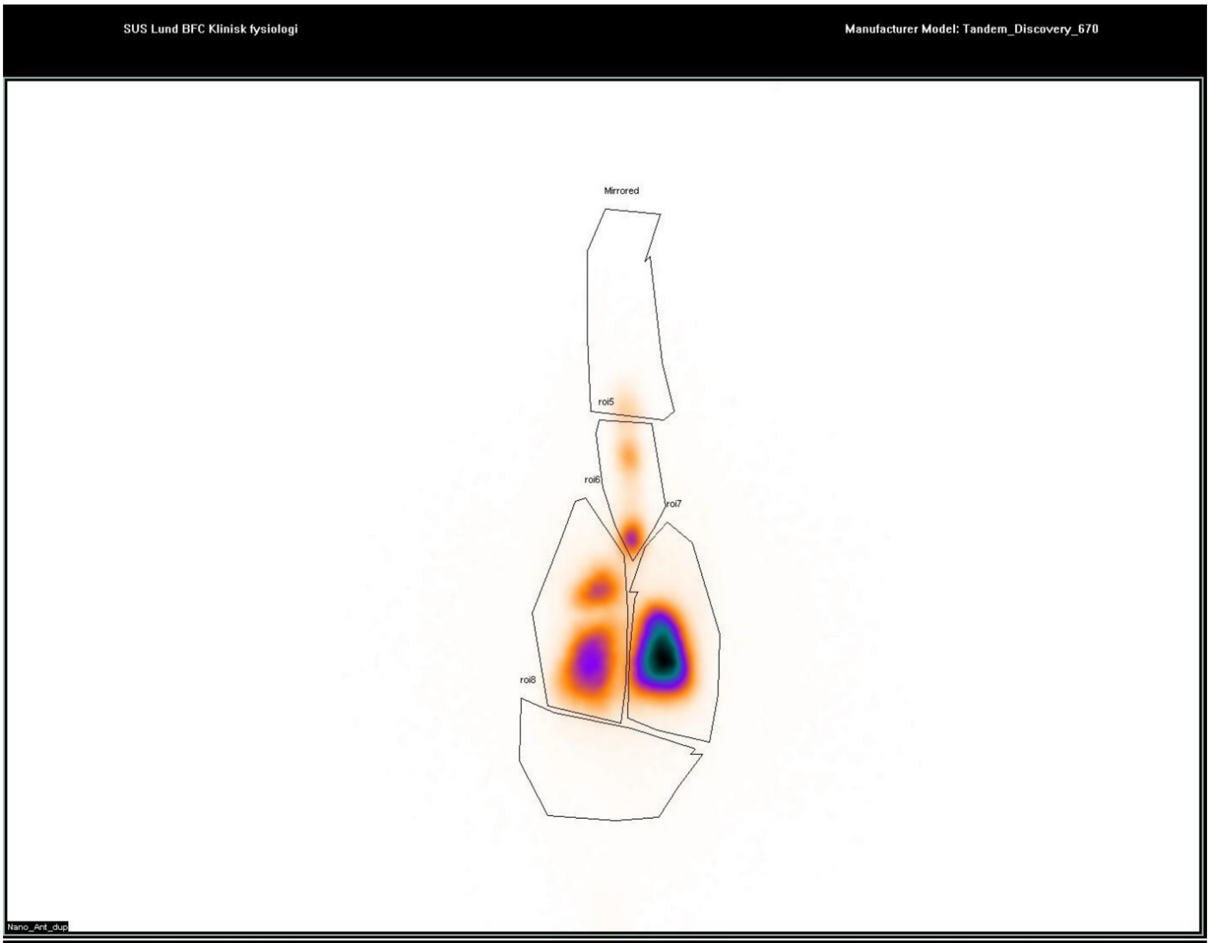
SUS Lund BFC Klinisk fysiologi
Manufacturer Model: Tandem_Discovery_670
Mirrored
roi5
roi6
roi7
roi8
Nano_Ant_dup

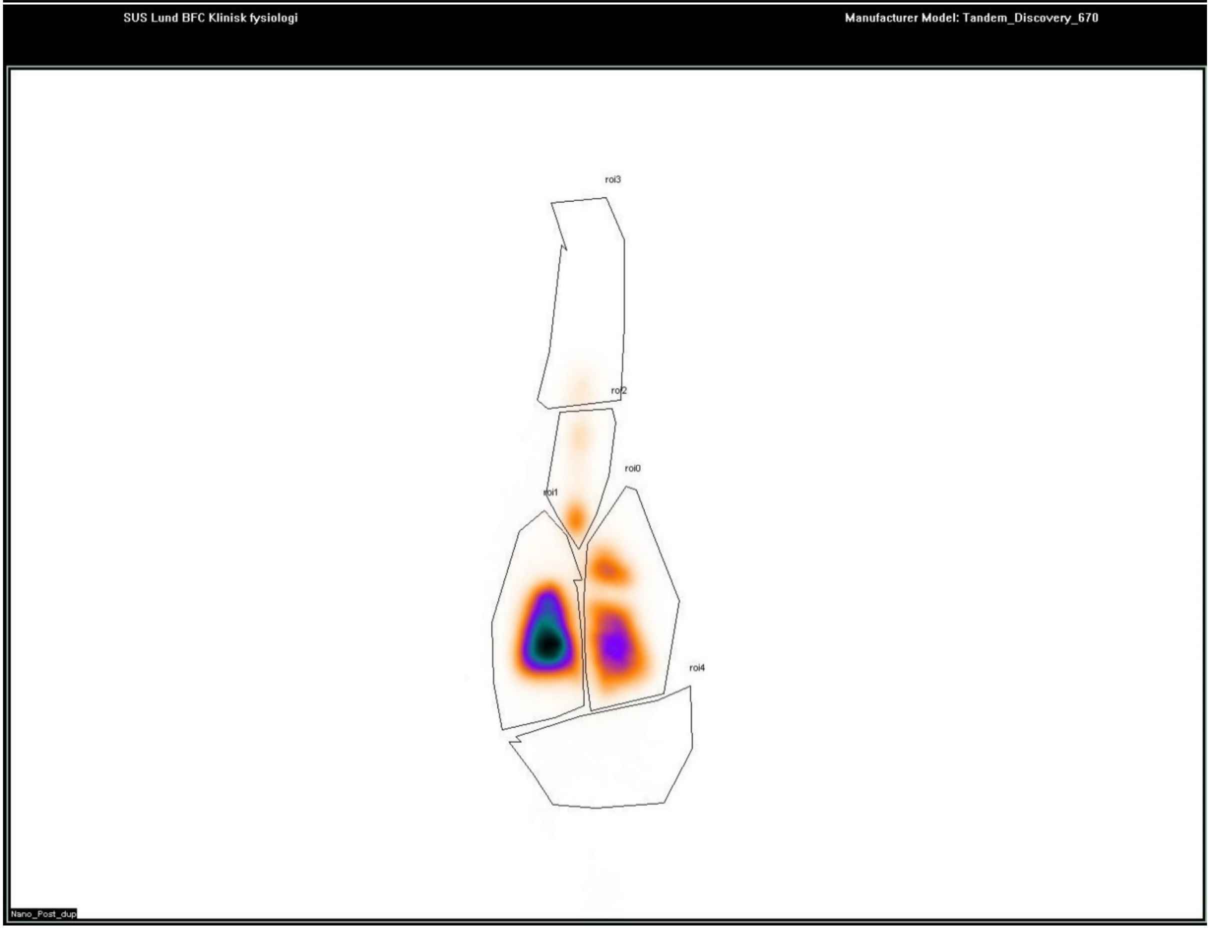
SUS Lund BFC Klinisk fysiologi
Manufacturer Model: Tandem_Discovery_670
roi3
roi2
roi0
roi1
roi4
Nano_Post_dup

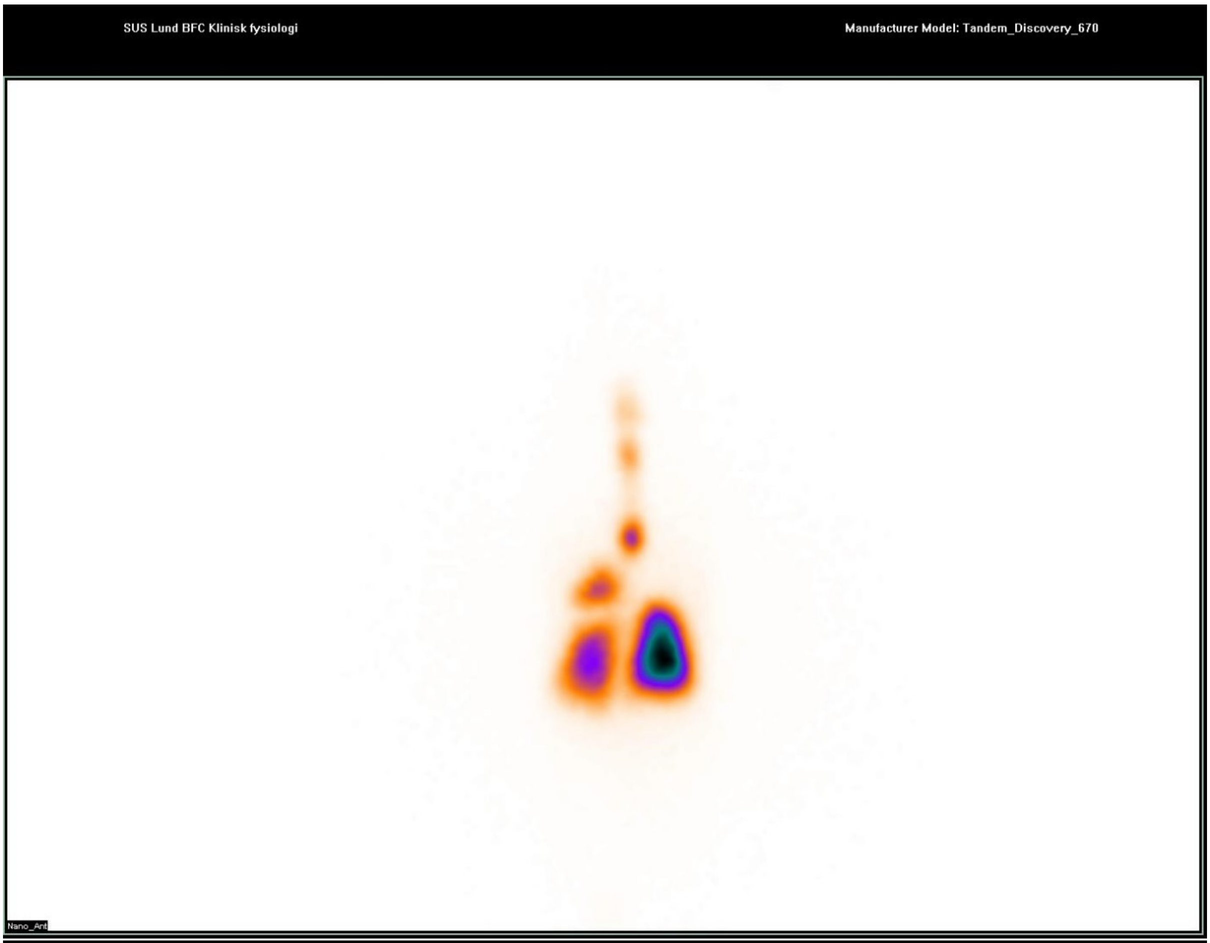
SUS Lund BFC Klinisk fysiologi
Manufacturer Model: Tandem_Discovery_670
Nano_Ant

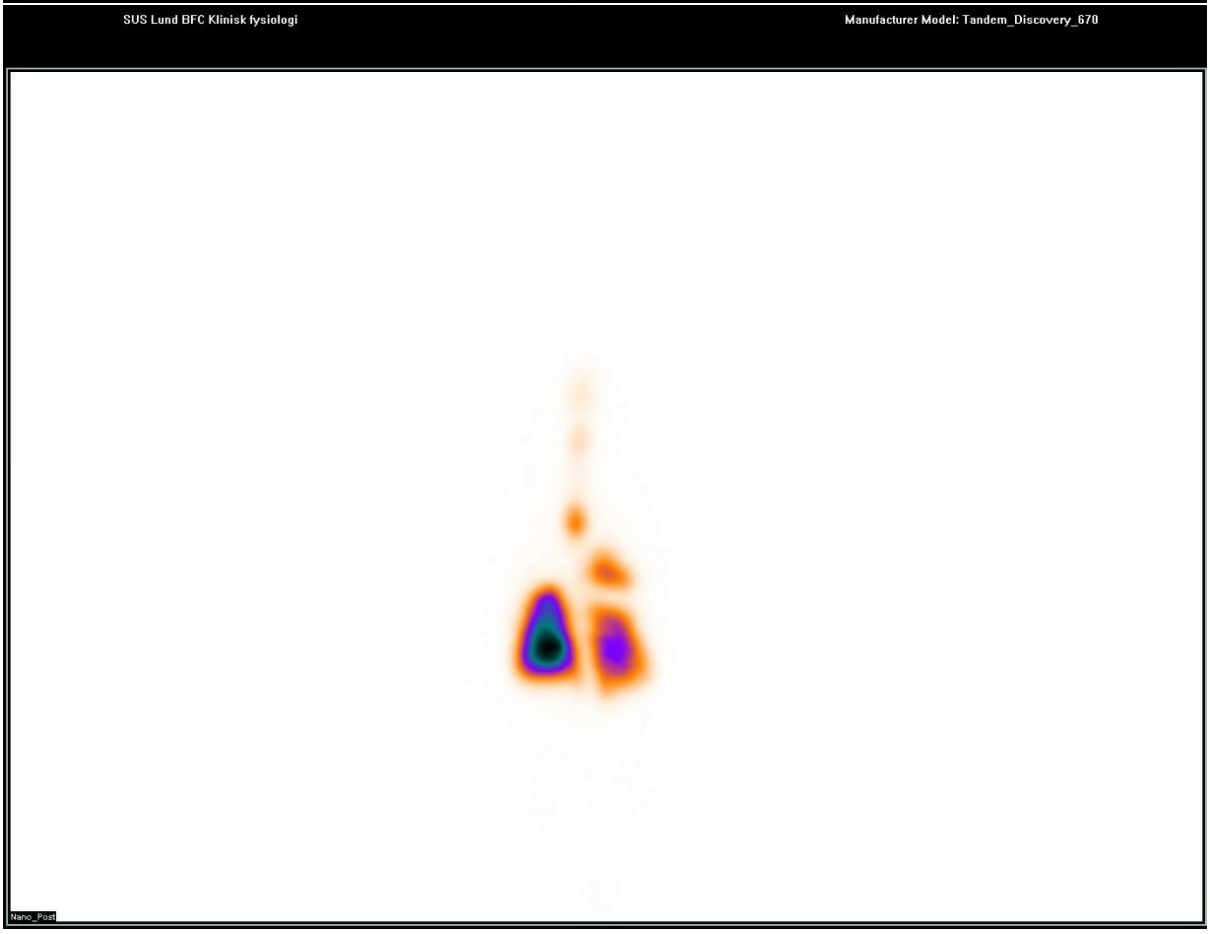
SUS Lund BFC Klinisk fysiologi
Manufacturer Model: Tandem_Discovery_670
Nano_Post

SUS Lund BFC Klinisk fysiologi

Manufacturer Model: Tandem_Discovery_670

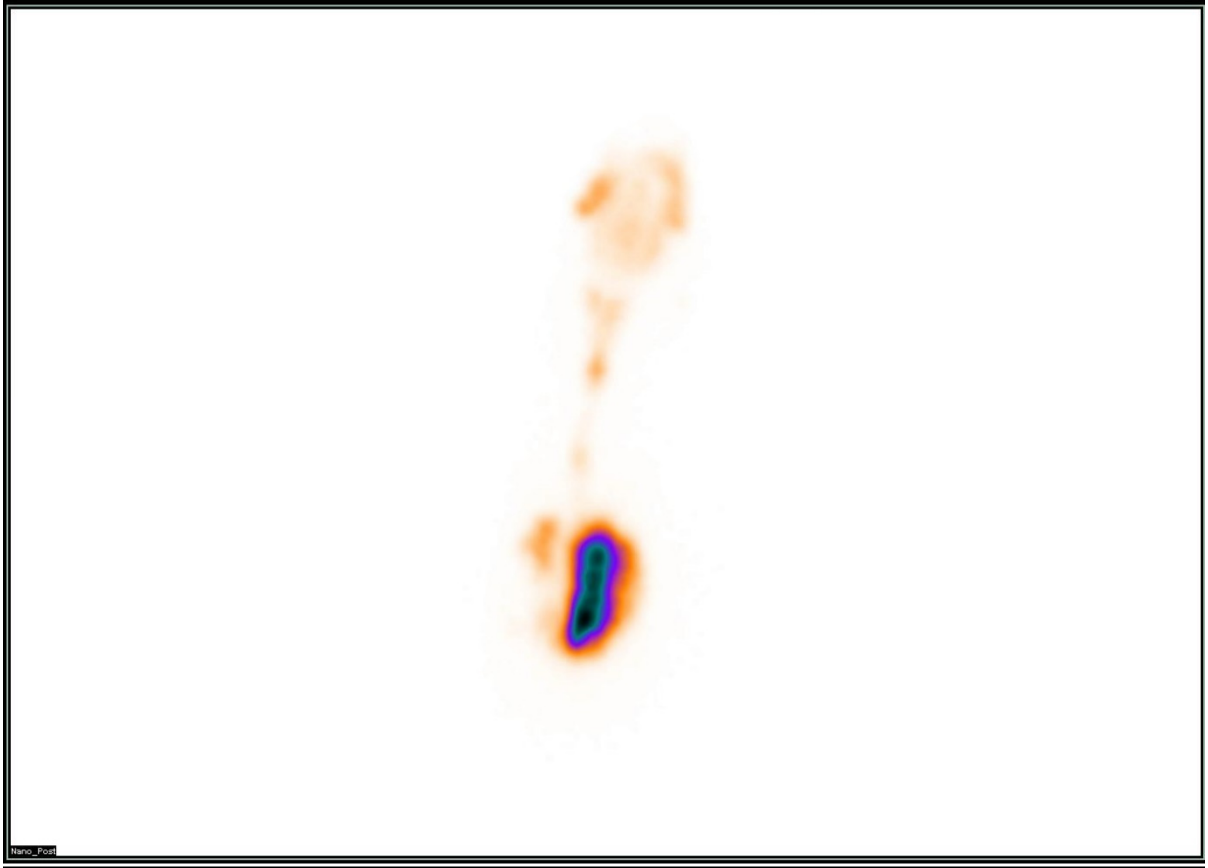

SUS Lund BFC Klinisk fysiologi

Manufacturer Model: Tandem_Discovery_670

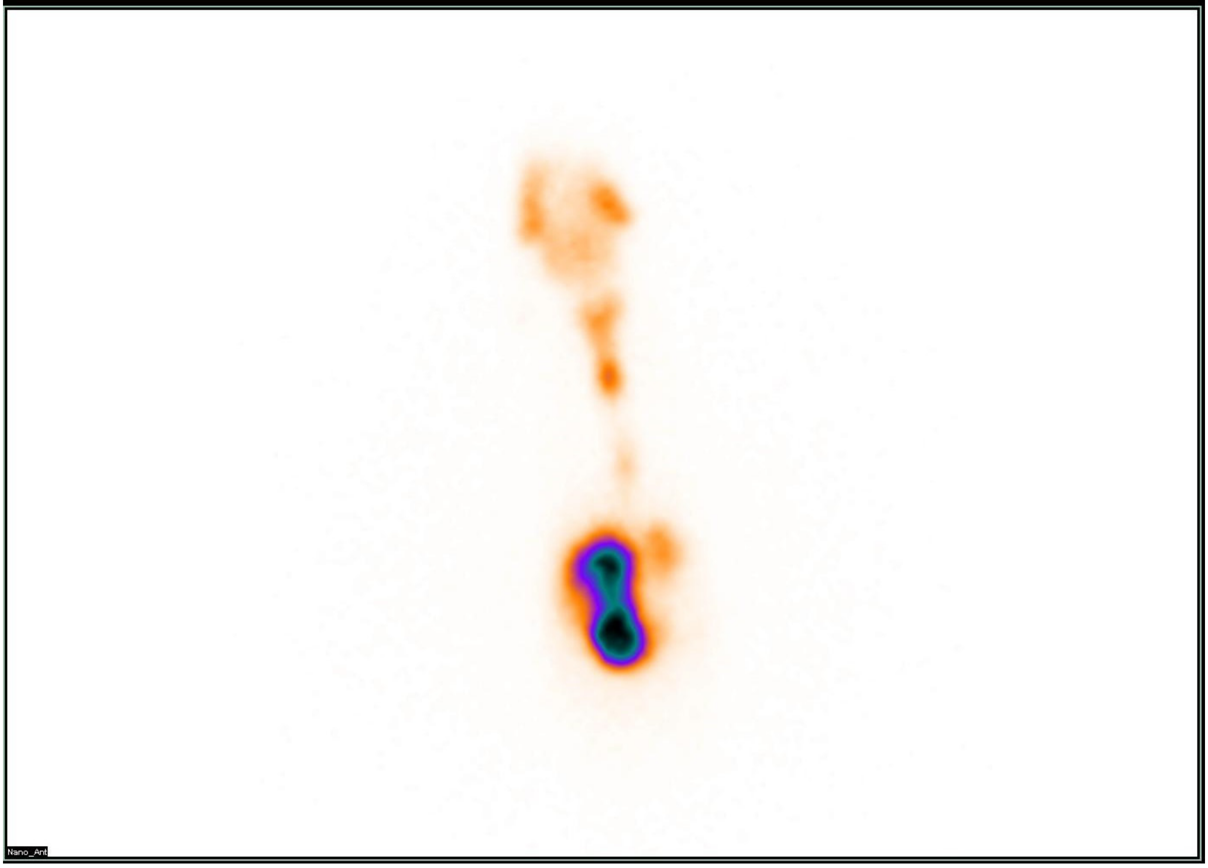

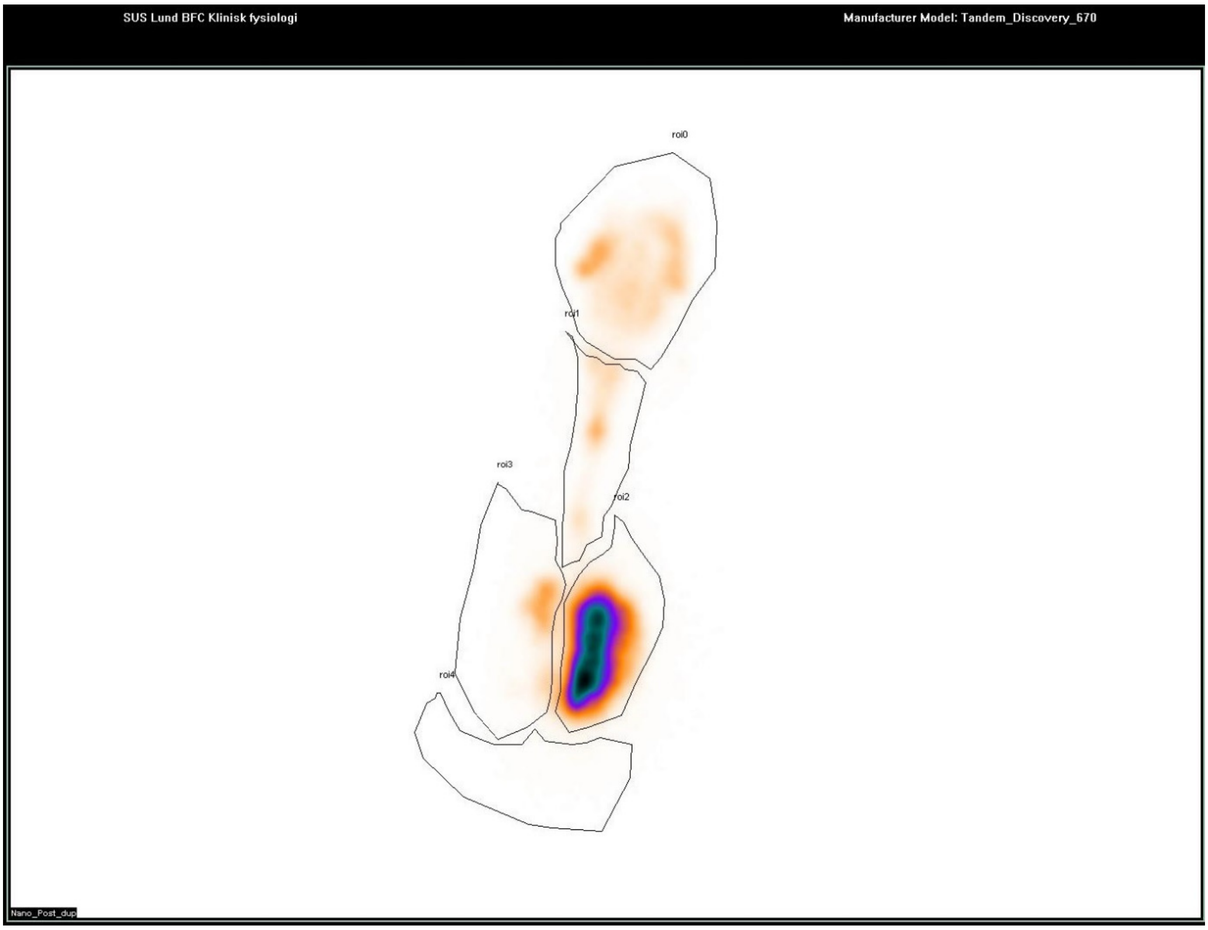
SUS Lund BFC Klinisk fysiologi
Manufacturer Model: Tandem_Discovery_670
roi0
roi1
roi3
roi2
roi4
Nano_Post_dup

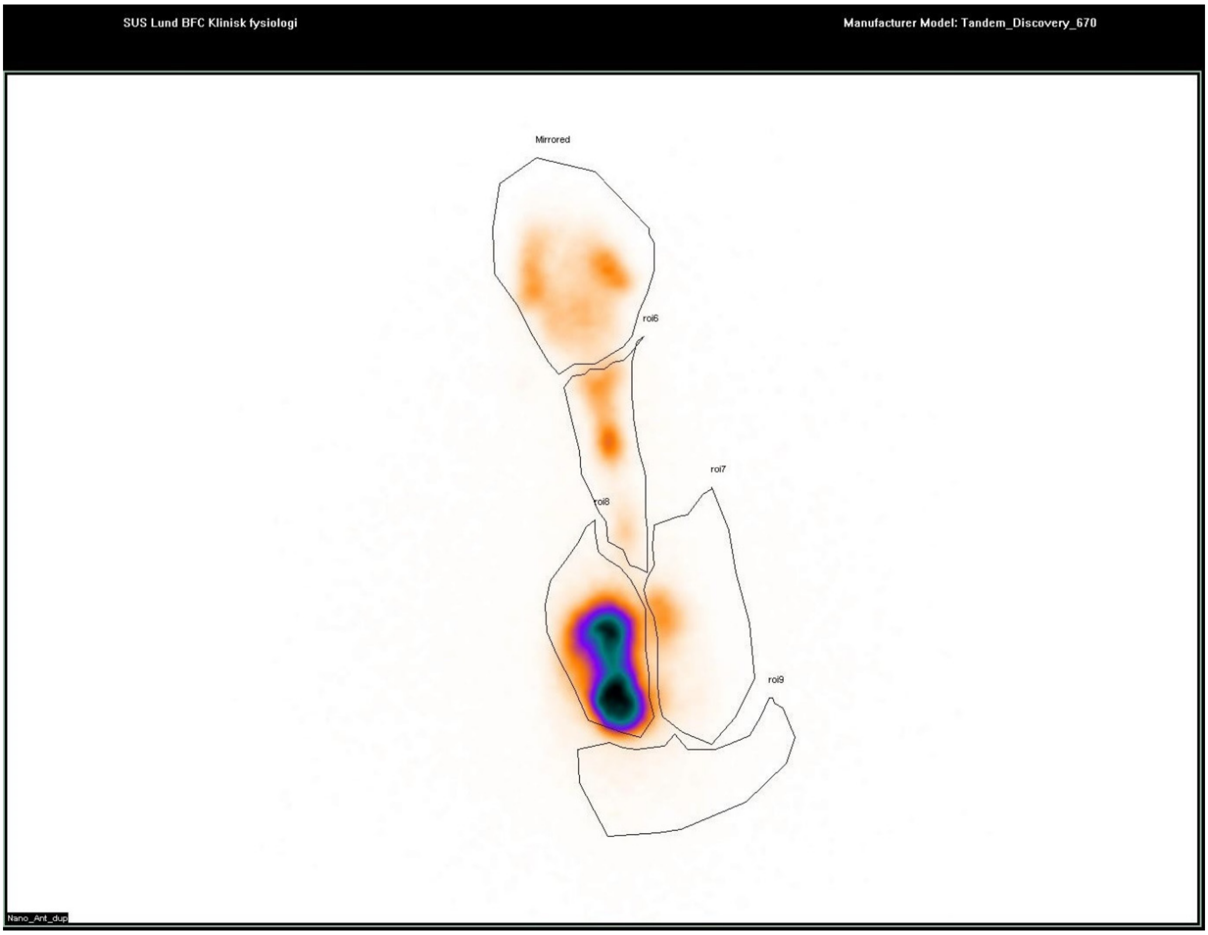
SUS Lund BFC Klinisk fysiologi
Manufacturer Model: Tandem_Discovery_670
Mirrored
roi6
roi7
roi8
roi9
Nano_Ant_dup

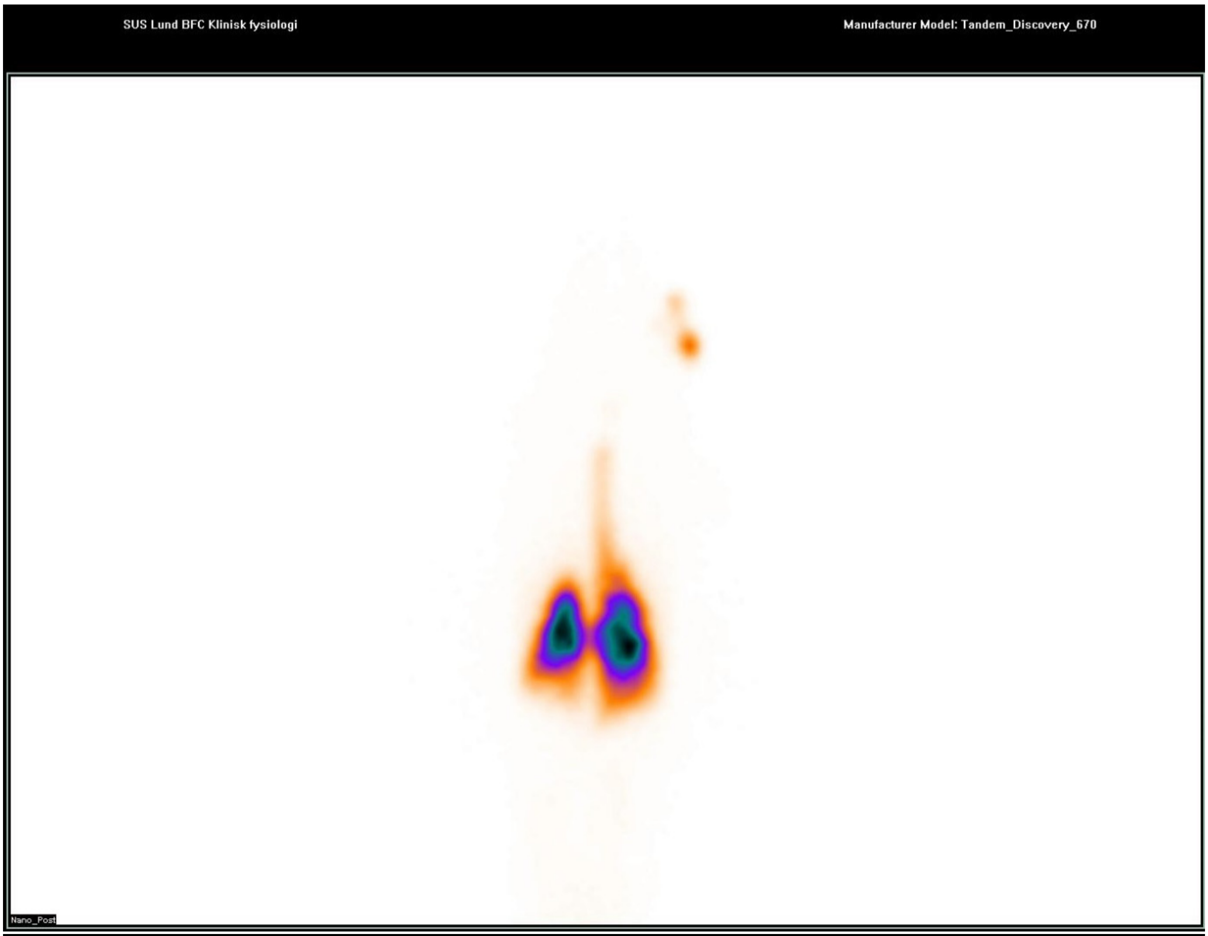
SUS Lund BFC Klinisk fysiologi
Manufacturer Model: Tandem_Discovery_670
Nano_Post

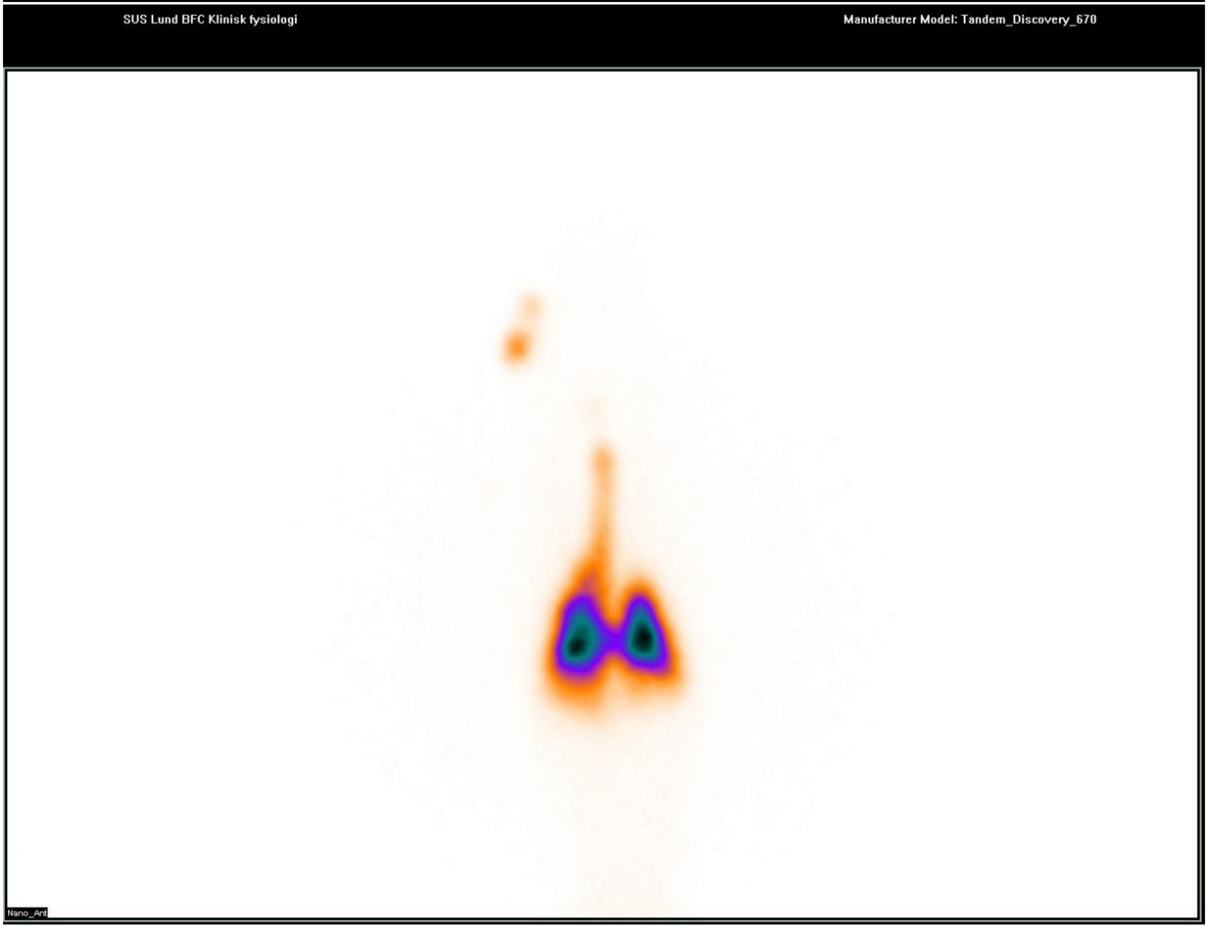
SUS Lund BFC Klinisk fysiologi
Manufacturer Model: Tandem_Discovery_670
Nano_Ant

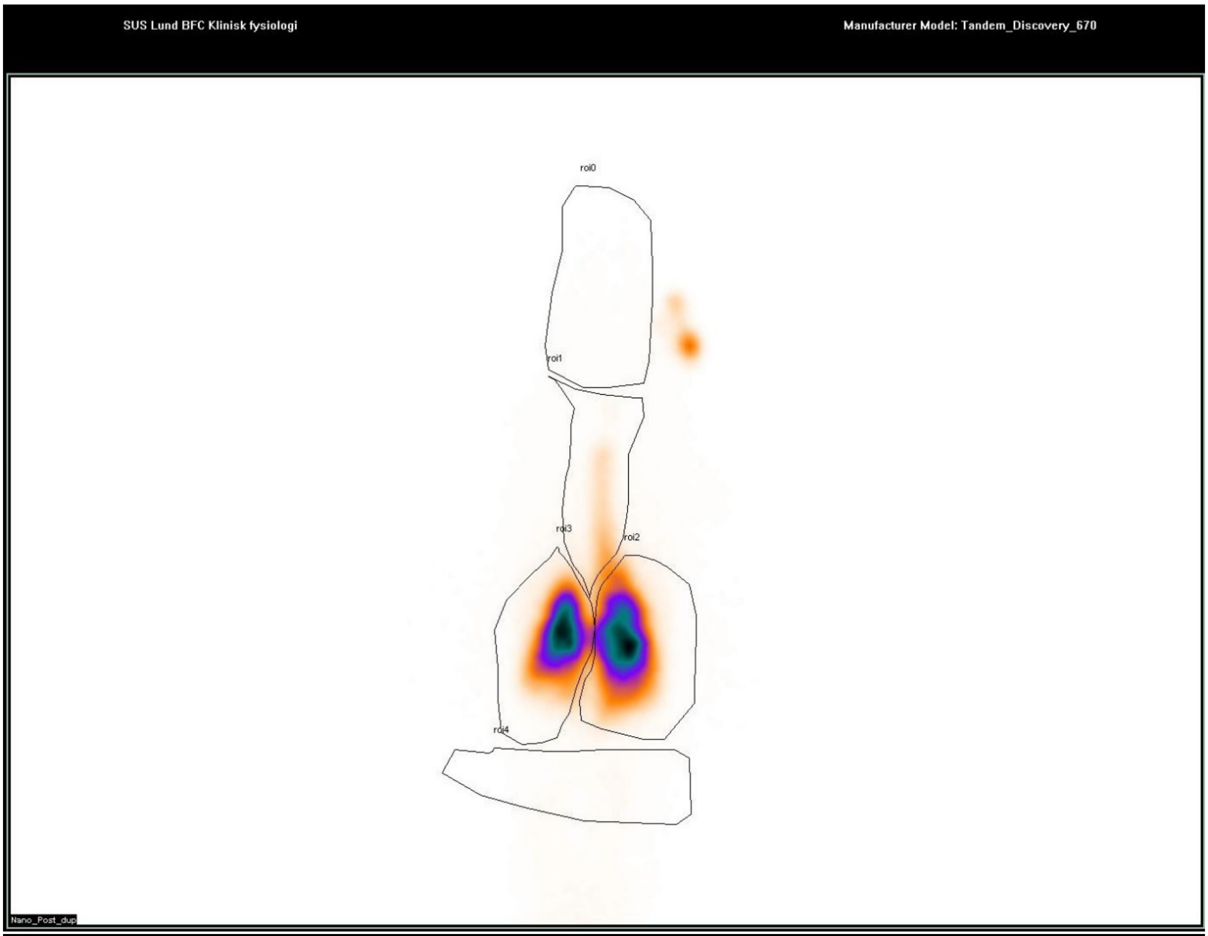
SUS Lund BFC Klinisk fysiologi
Manufacturer Model: Tandem_Discovery_670
roi0
roi1
roi3
roi2
roi4
Nano_Post_dup

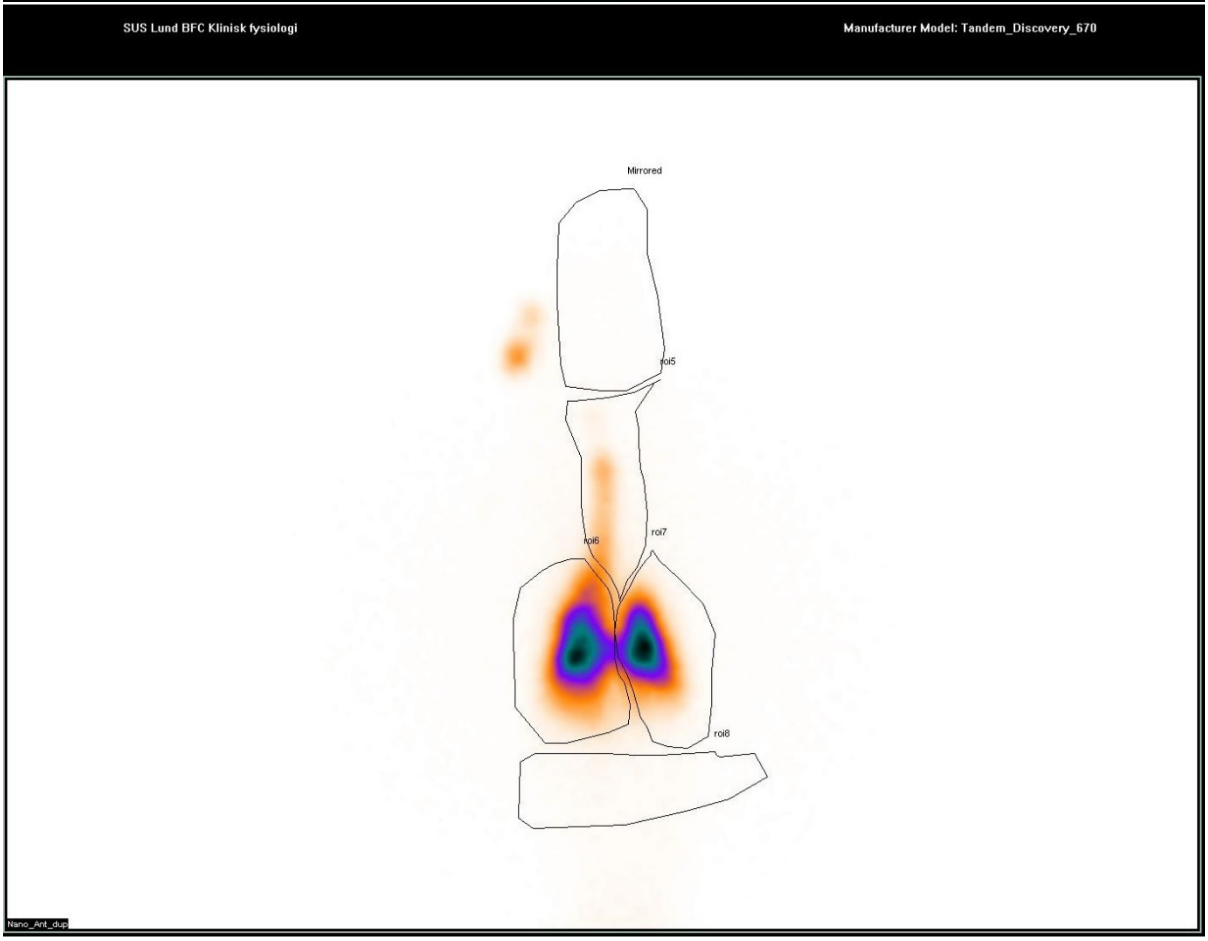
SUS Lund BFC Klinisk fysiologi
Manufacturer Model: Tandem_Discovery_670
Mirrored
roi5
roi6
roi7
roi8
Nano_Ant_dup

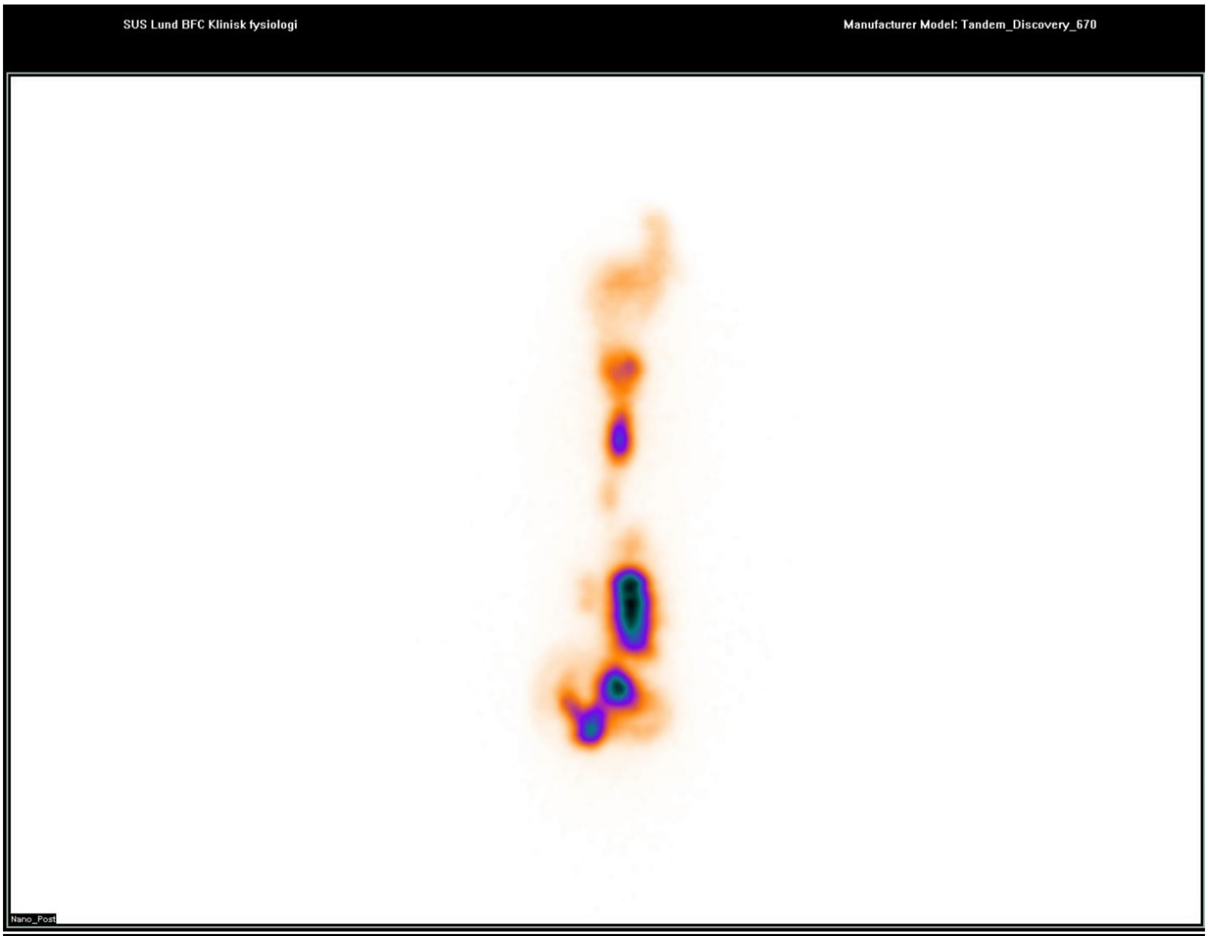
SUS Lund BFC Klinisk fysiologi
Manufacturer Model: Tandem_Discovery_670
Nano_Post

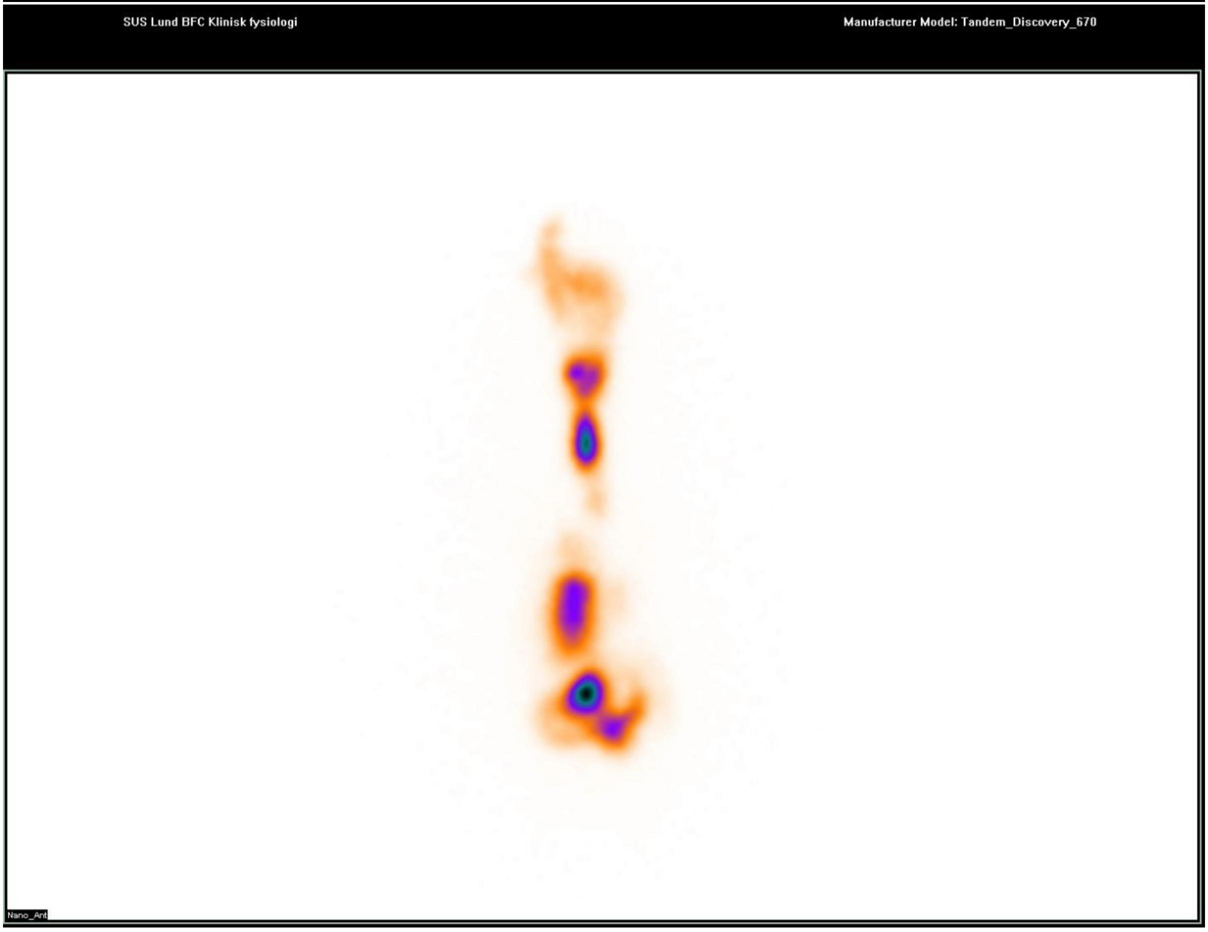
SUS Lund BFC Klinisk fysiologi
Manufacturer Model: Tandem_Discovery_670
Nano_Ant

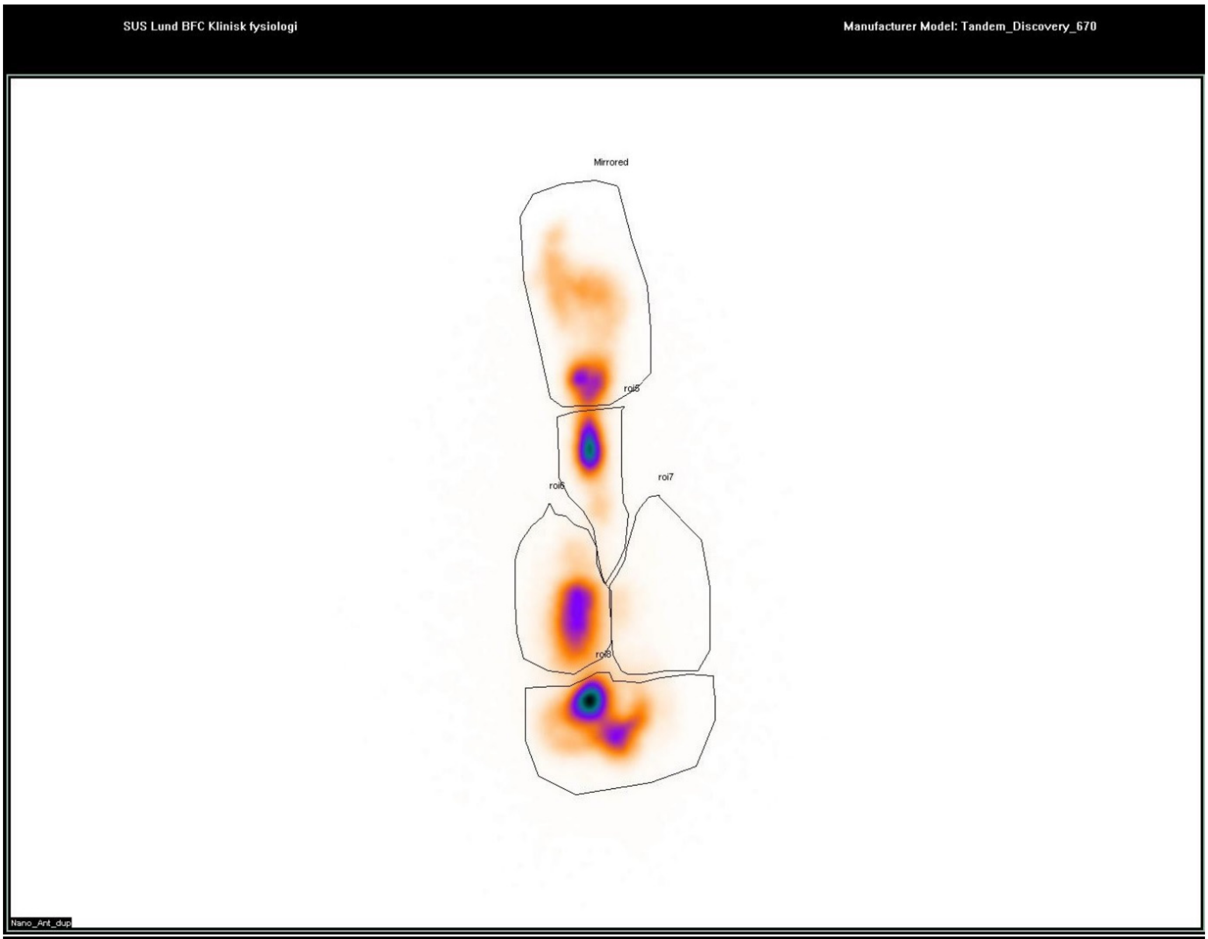
SUS Lund BFC Klinisk fysiologi
Manufacturer Model: Tandem_Discovery_670
Mirrored
roi5
roi6
roi7
roi8
Nano_Ant_dup

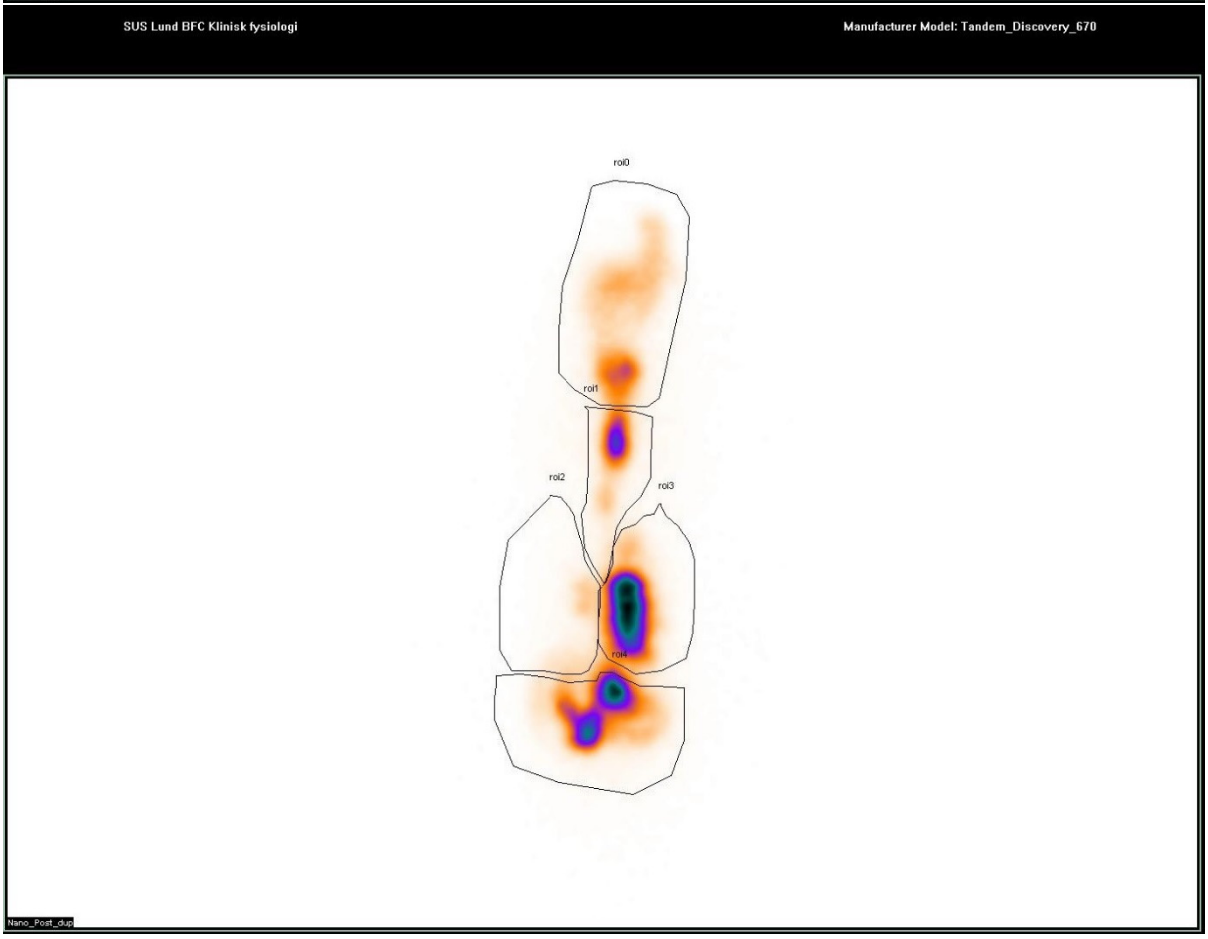
SUS Lund BFC Klinisk fysiologi
Manufacturer Model: Tandem_Discovery_670
roi0
roi1
roi2
roi3
roi4
Nano_Post_dup

SUS Lund BFC Klinisk fysiologi

Manufacturer Model: Tandem_Discovery_670

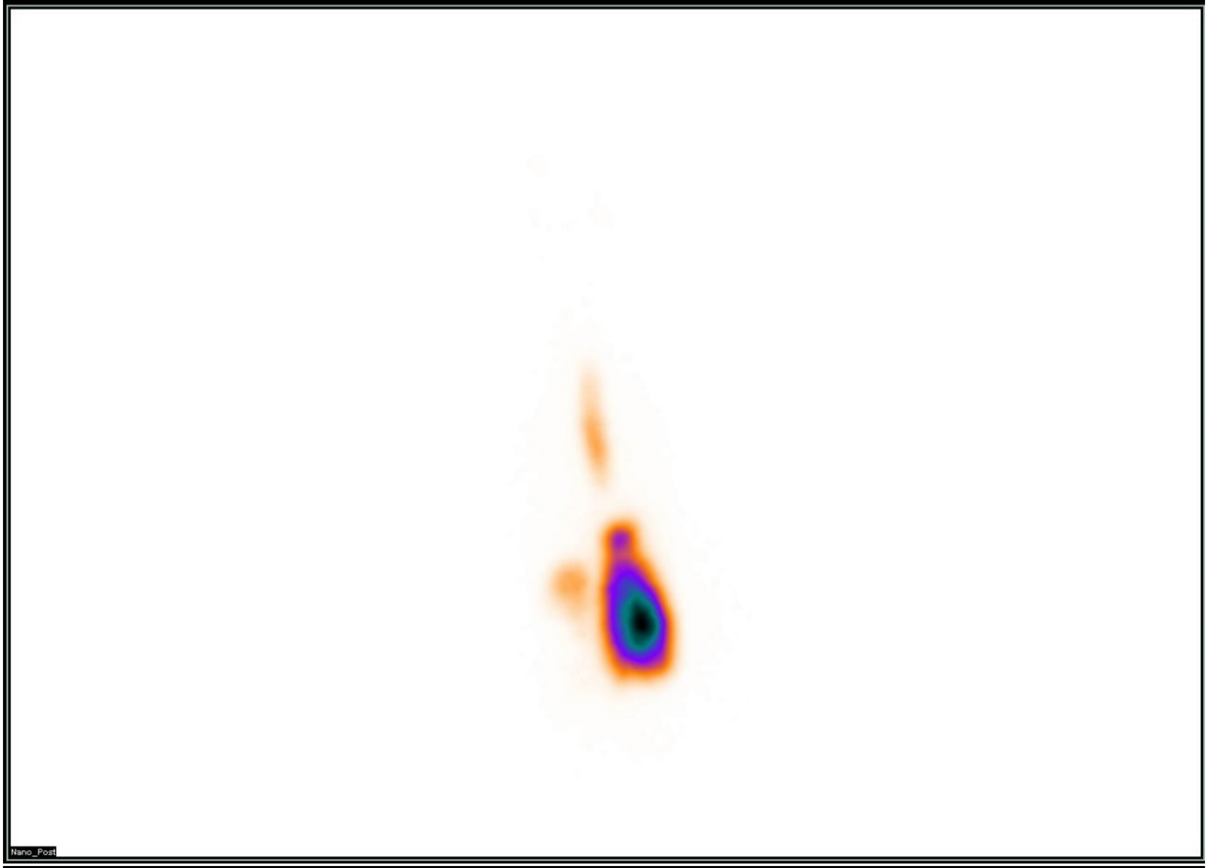

SUS Lund BFC Klinisk fysiologi

Manufacturer Model: Tandem_Discovery_670

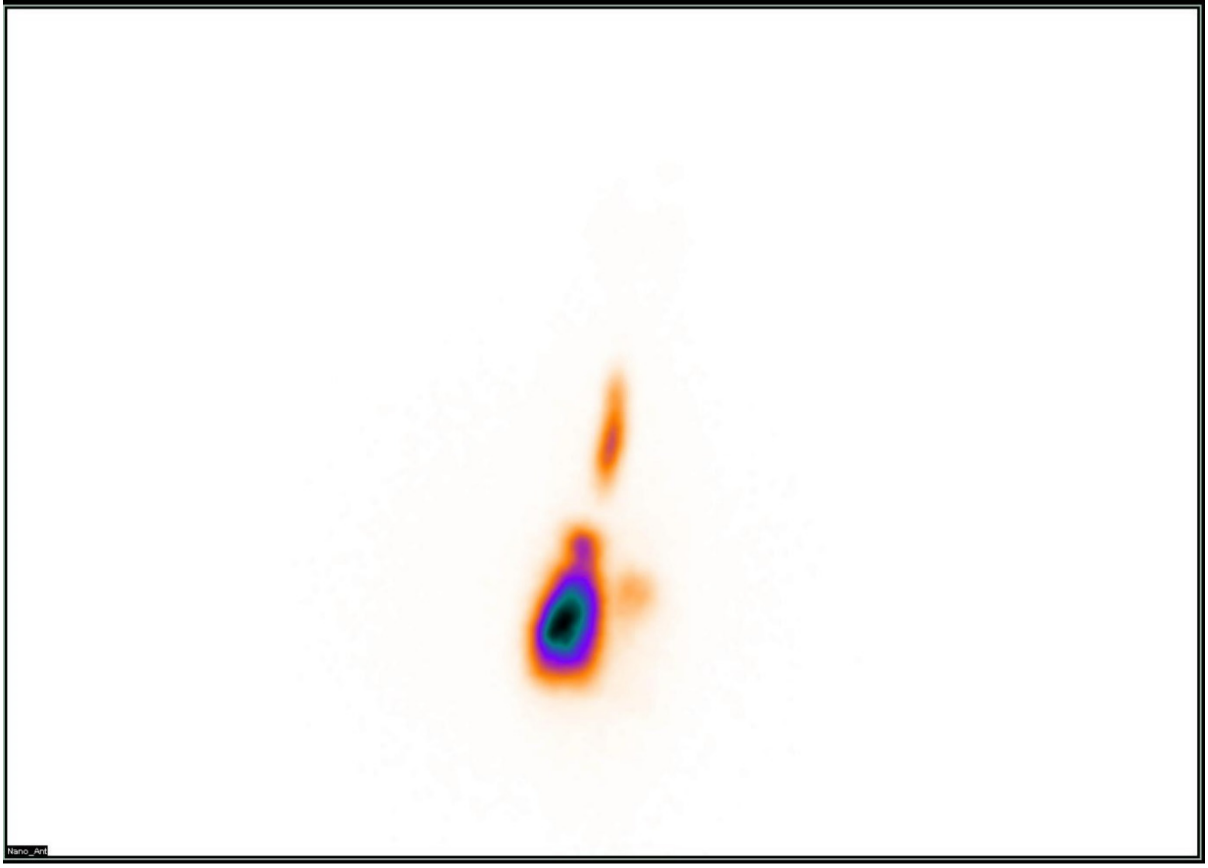

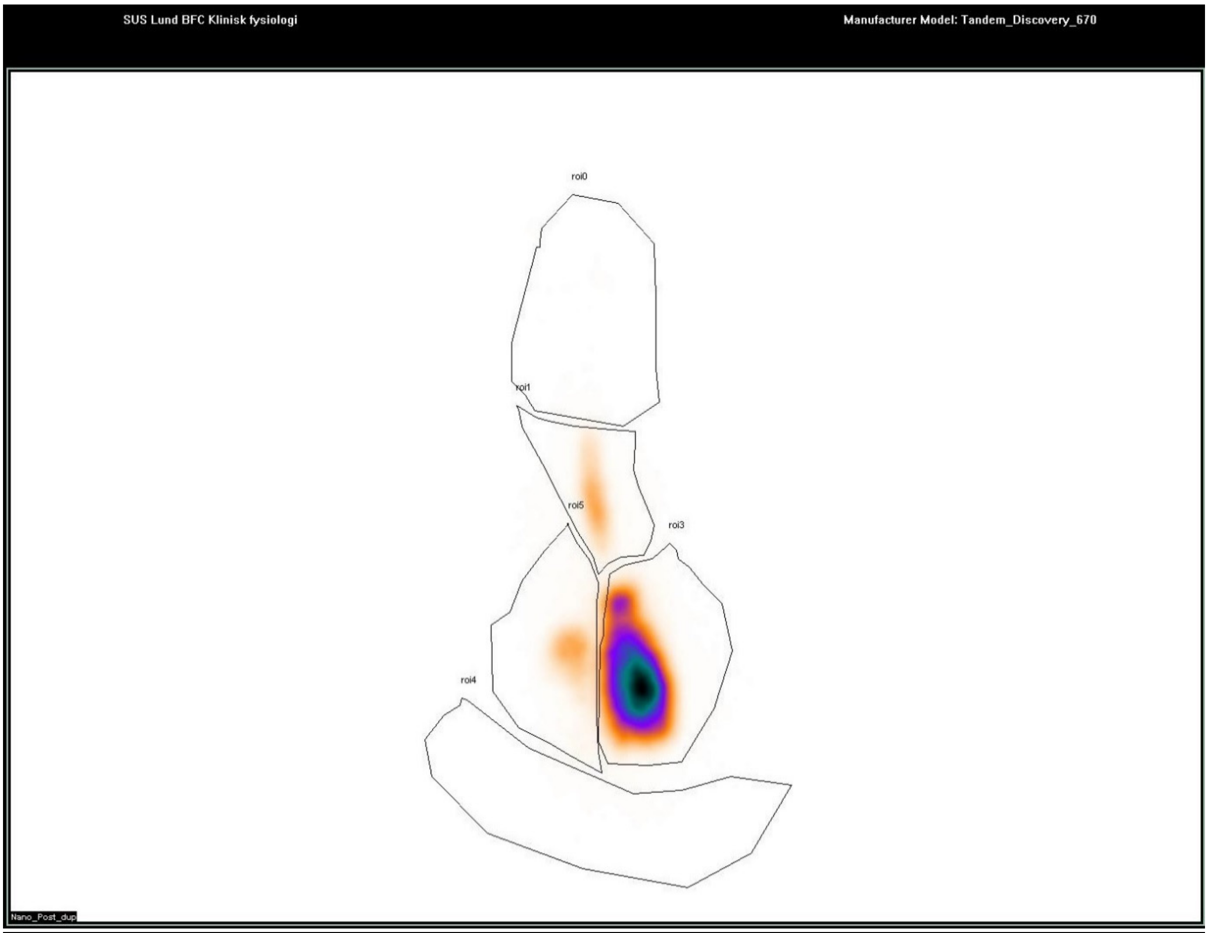
SUS Lund BFC Klinisk fysiologi
Manufacturer Model: Tandem_Discovery_670
roi0
roi1
roi5
roi3
roi4
Nano_Post_dup

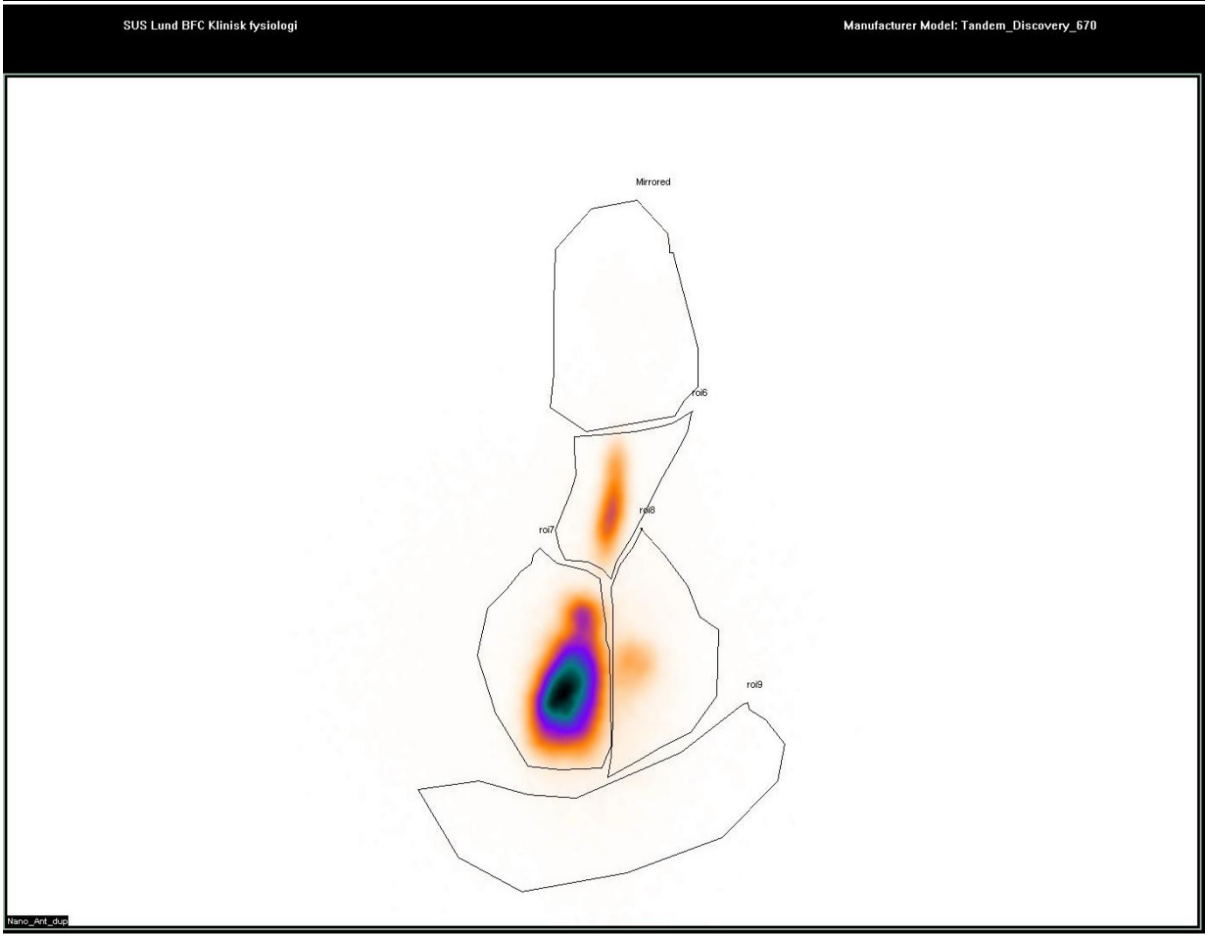
SUS Lund BFC Klinisk fysiologi
Manufacturer Model: Tandem_Discovery_670
Mirrored
roi6
roi8
roi7
roi9
Nano_Ant_dup

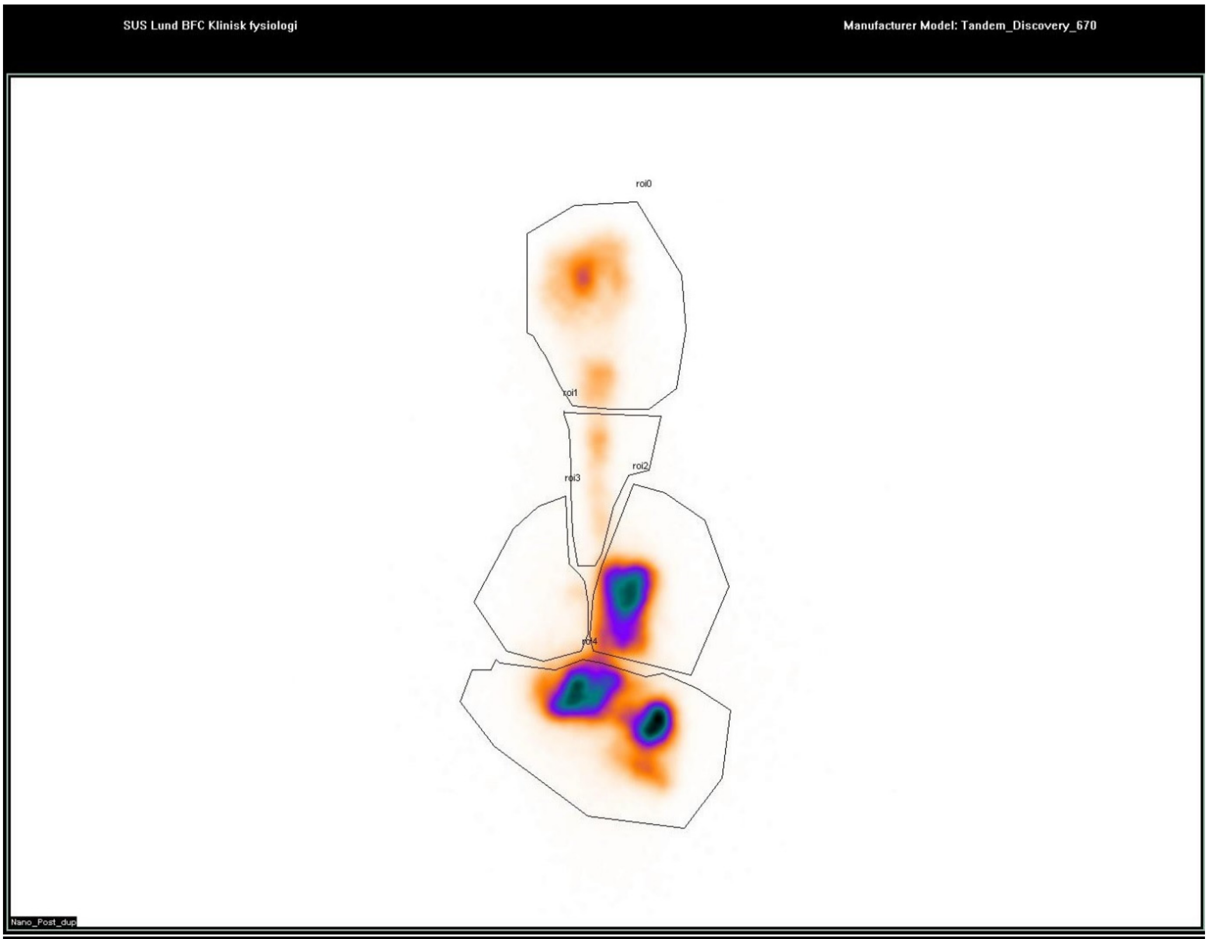
SUS Lund BFC Klinisk fysiologi
Manufacturer Model: Tandem_Discovery_670
roi0
roi1
roi2
roi3
roi4
Nano_Post_dup

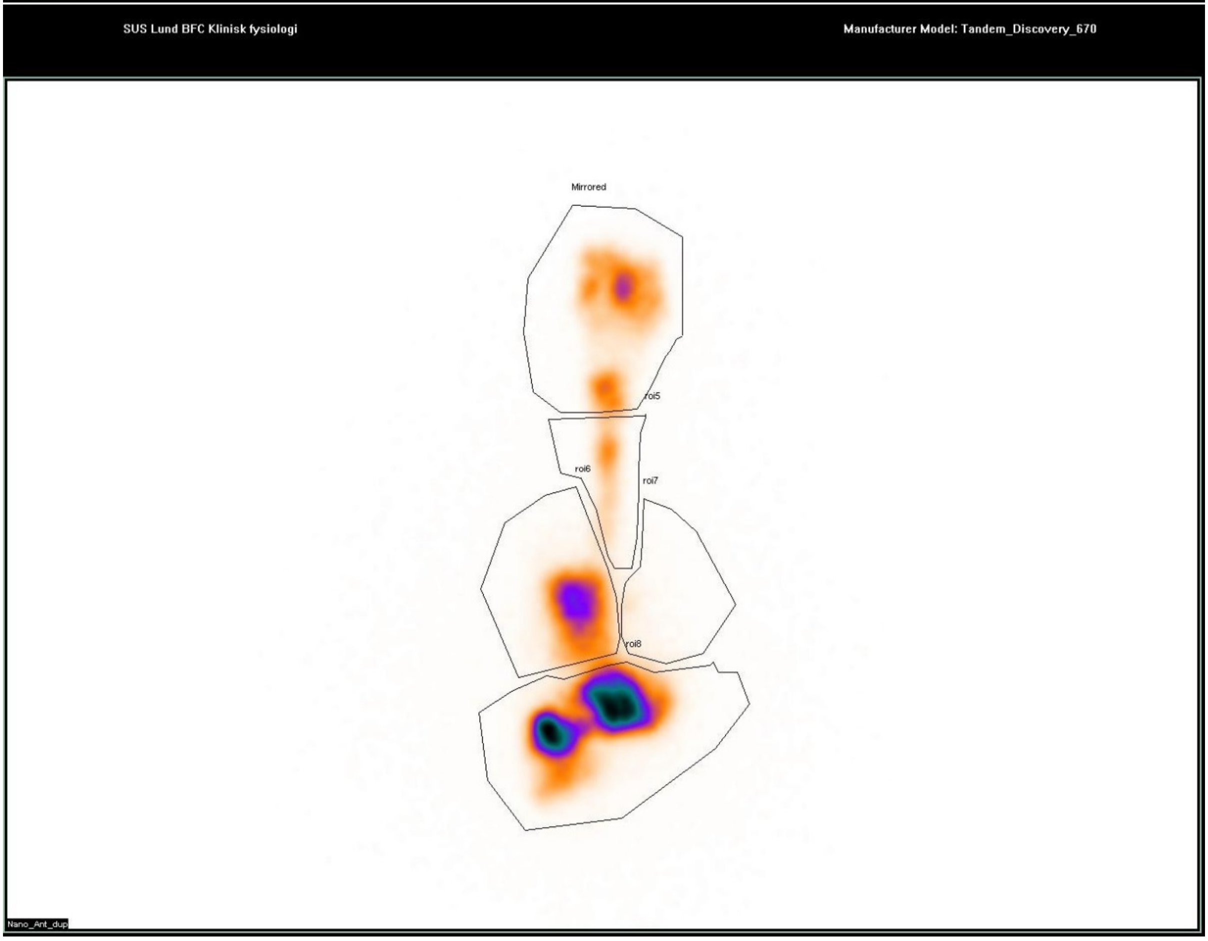
SUS Lund BFC Klinisk fysiologi
Manufacturer Model: Tandem_Discovery_670
Mirrored
roi5
roi6
roi7
roi8
Nano_Ant_dup

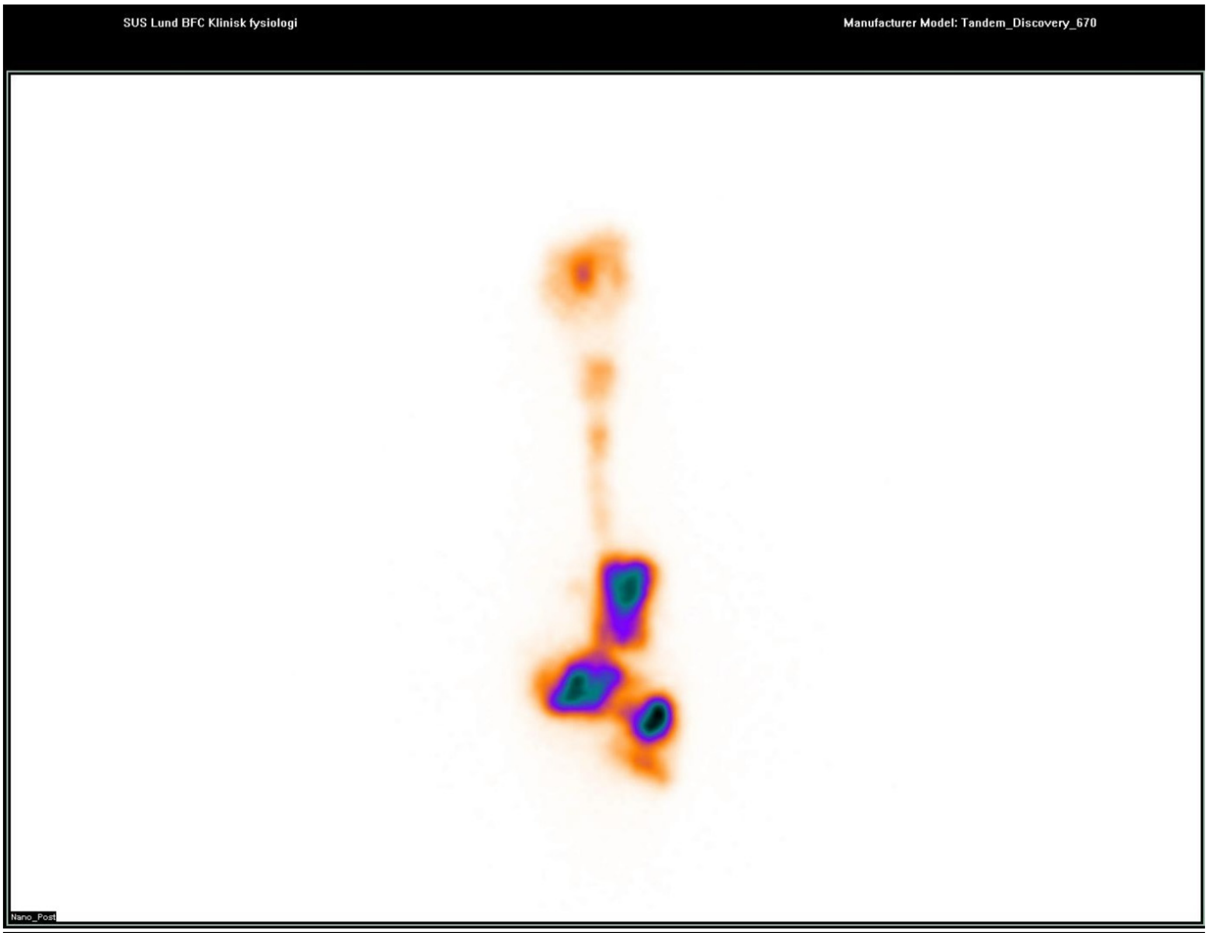
SUS Lund BFC Klinisk fysiologi
Manufacturer Model: Tandem_Discovery_670
Nano_Post

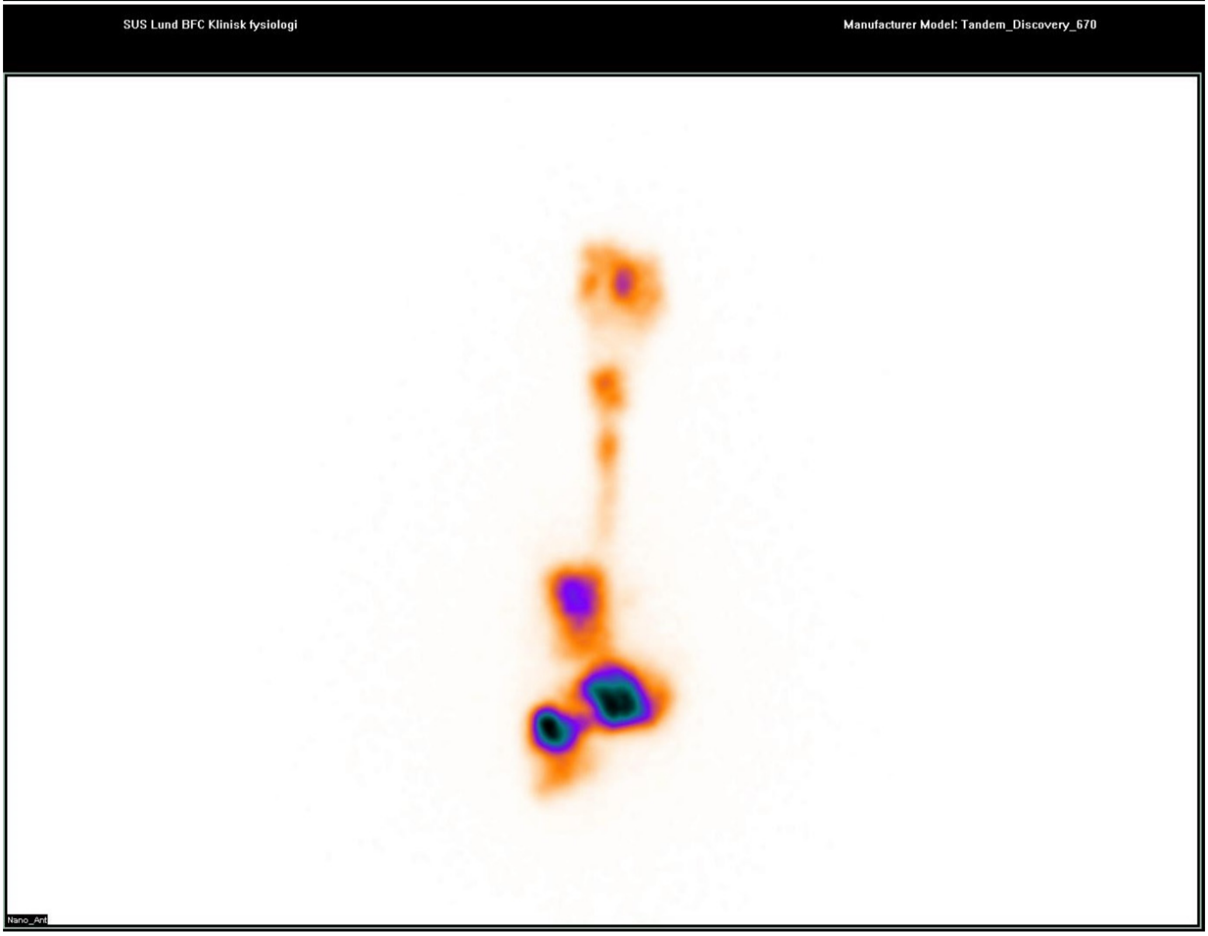
SUS Lund BFC Klinisk fysiologi
Manufacturer Model: Tandem_Discovery_670
Nano_Ant

SUS Lund BFC Klinisk fysiologi

Manufacturer Model: Tandem_Discovery_670

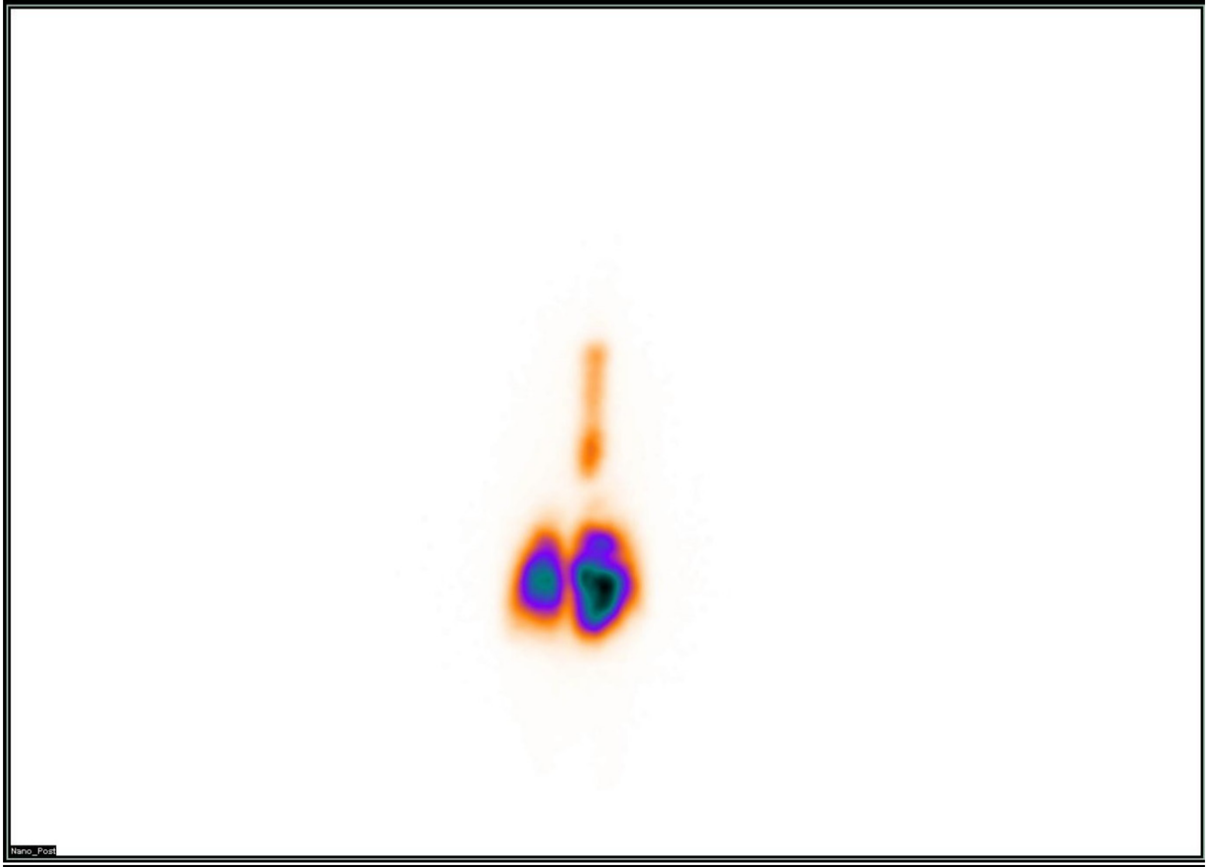

SUS Lund BFC Klinisk fysiologi

Manufacturer Model: Tandem_Discovery_670

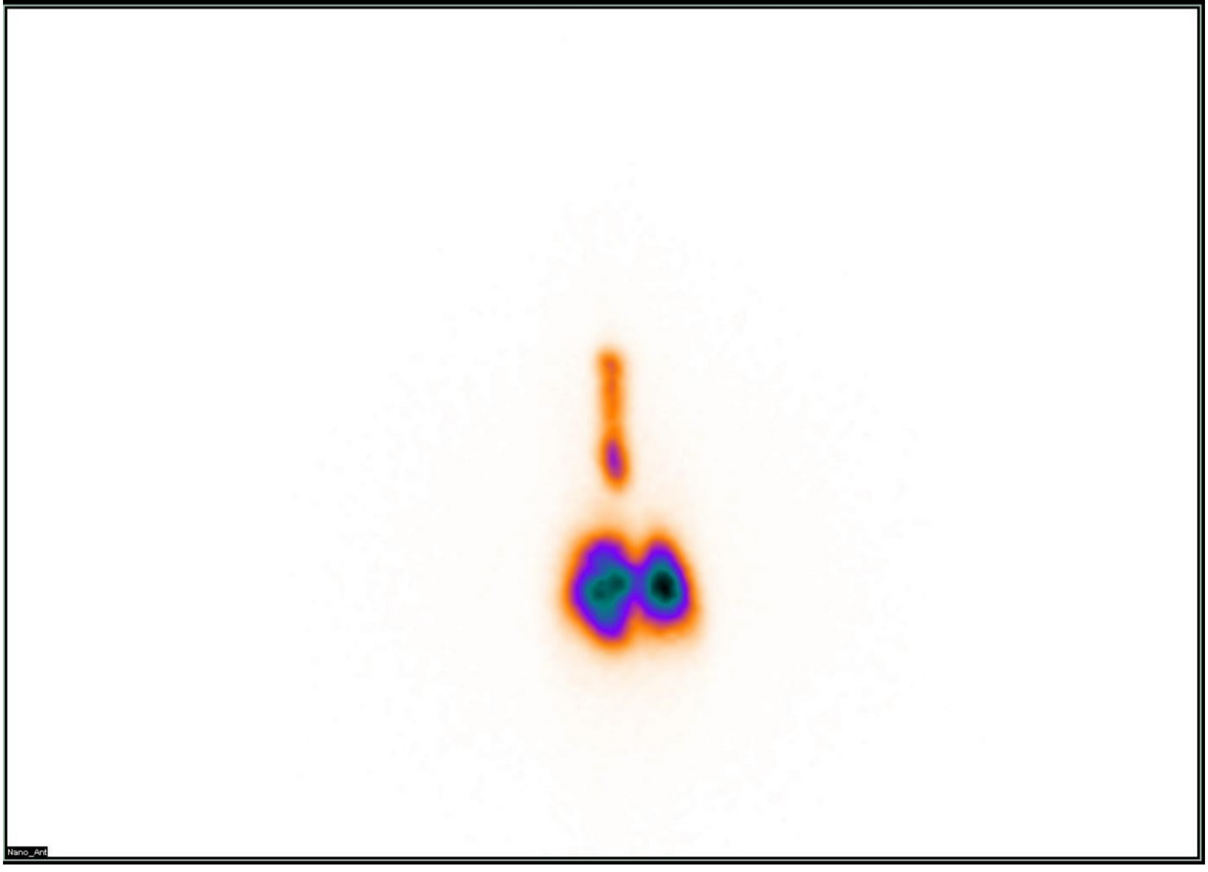

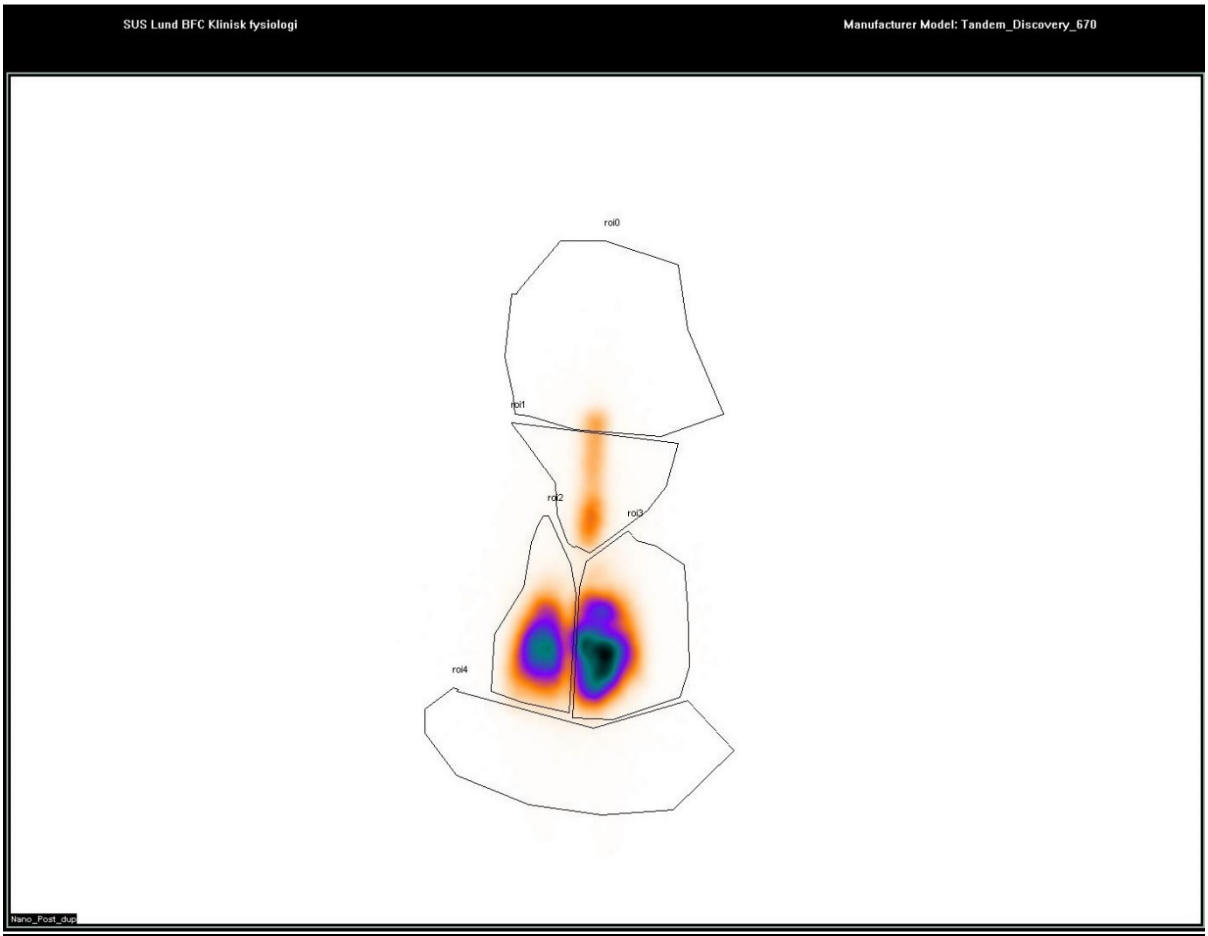
SUS Lund BFC Klinisk fysiologi
Manufacturer Model: Tandem_Discovery_670
roi0
roi1
roi2
roi3
roi4
Nano_Post_dup

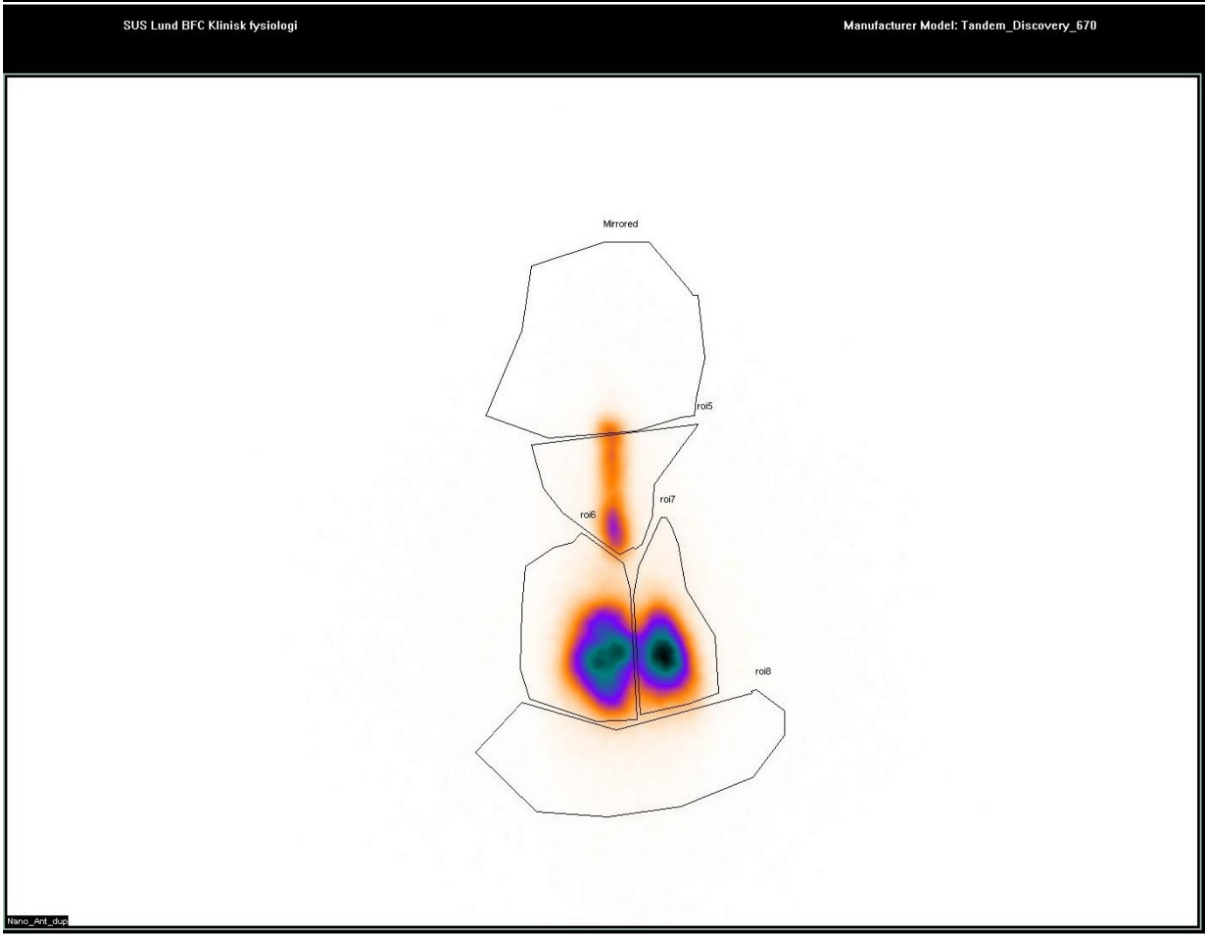
SUS Lund BFC Klinisk fysiologi
Manufacturer Model: Tandem_Discovery_670
Mirrored
roi5
roi6
roi7
roi8
Nano_Ant_dup

SUS Lund BFC Klinisk fysiologi

Manufacturer Model: Tandem_Discovery_670

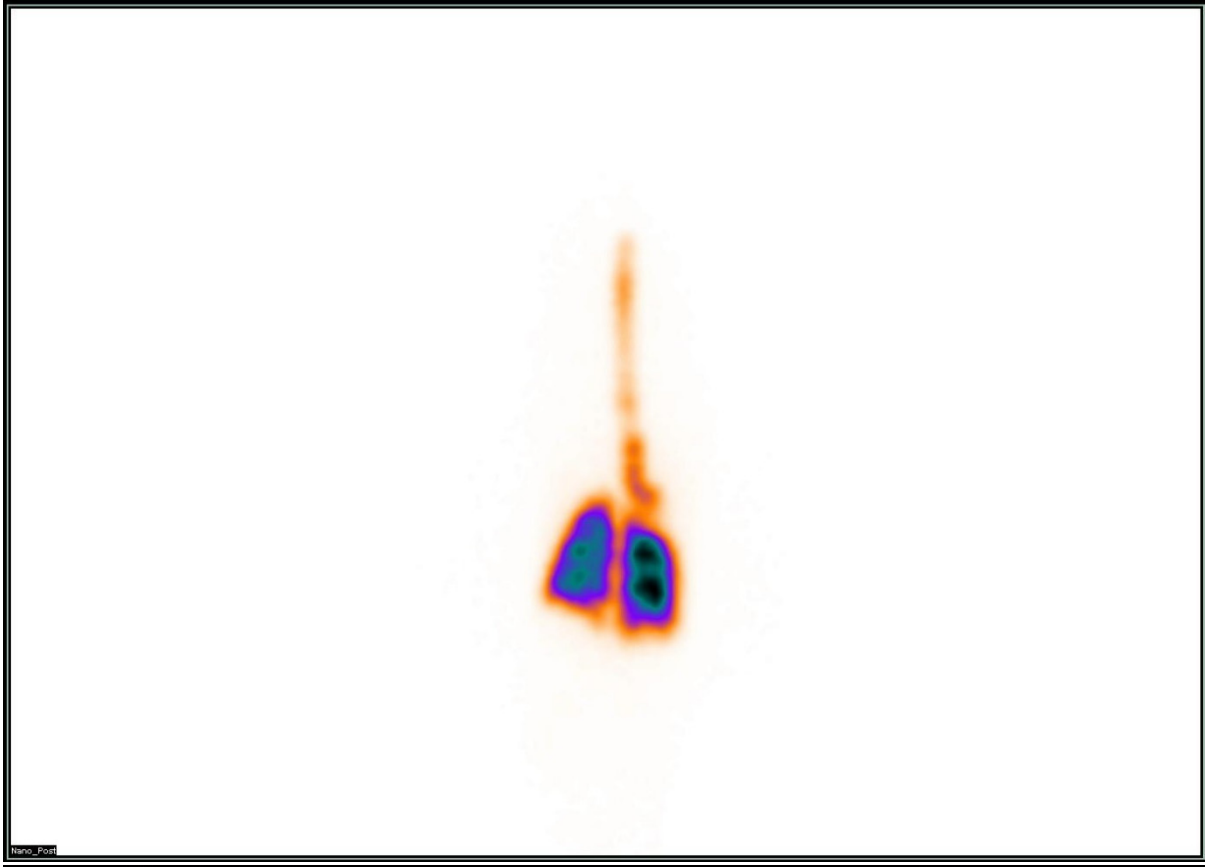

SUS Lund BFC Klinisk fysiologi

Manufacturer Model: Tandem_Discovery_670

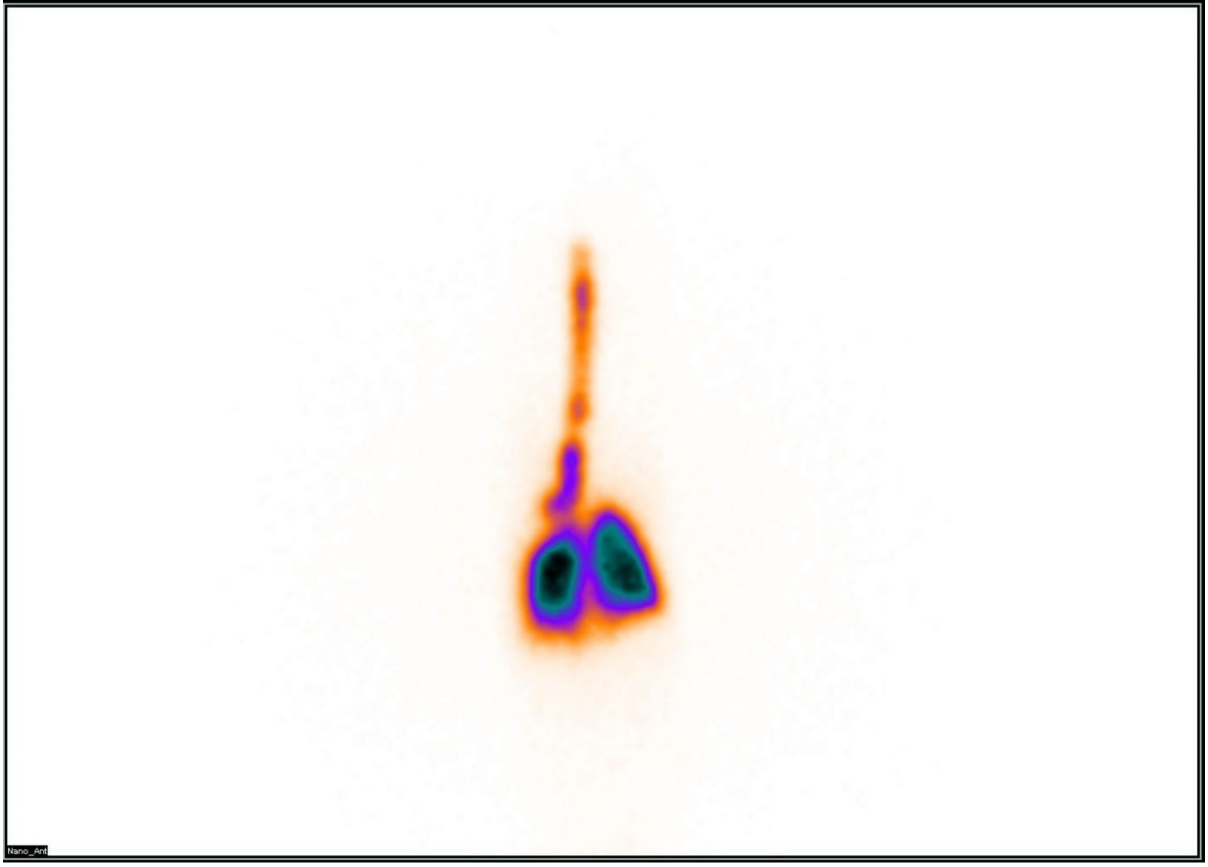

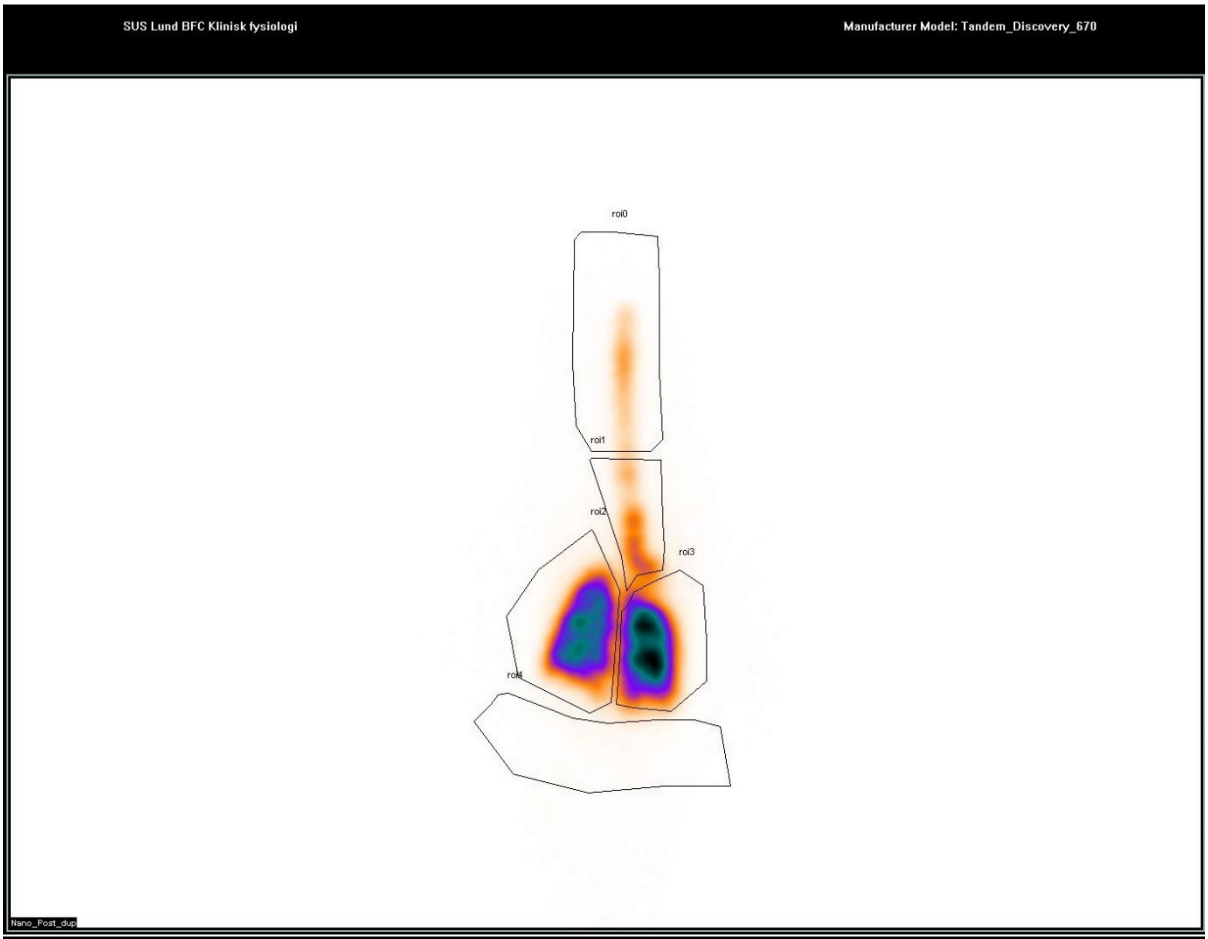
SUS Lund BFC Klinisk fysiologi
Manufacturer Model: Tandem_Discovery_670
roi0
roi1
roi2
roi3
roi4
Nano_Post_dup

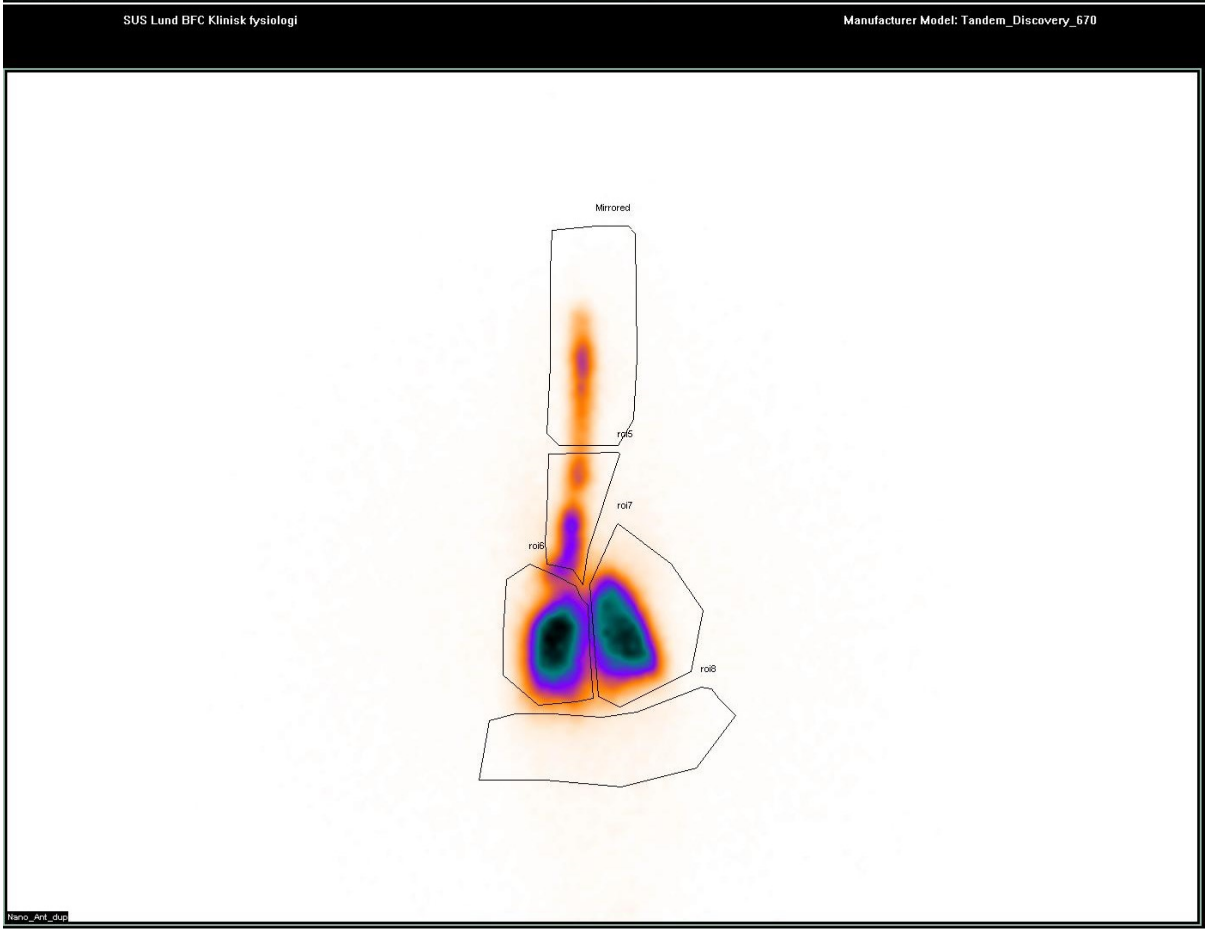
SUS Lund BFC Klinisk fysiologi
Manufacturer Model: Tandem_Discovery_670
Mirrored
roi5
roi7
roi6
roi8
Nano_Ant_dup

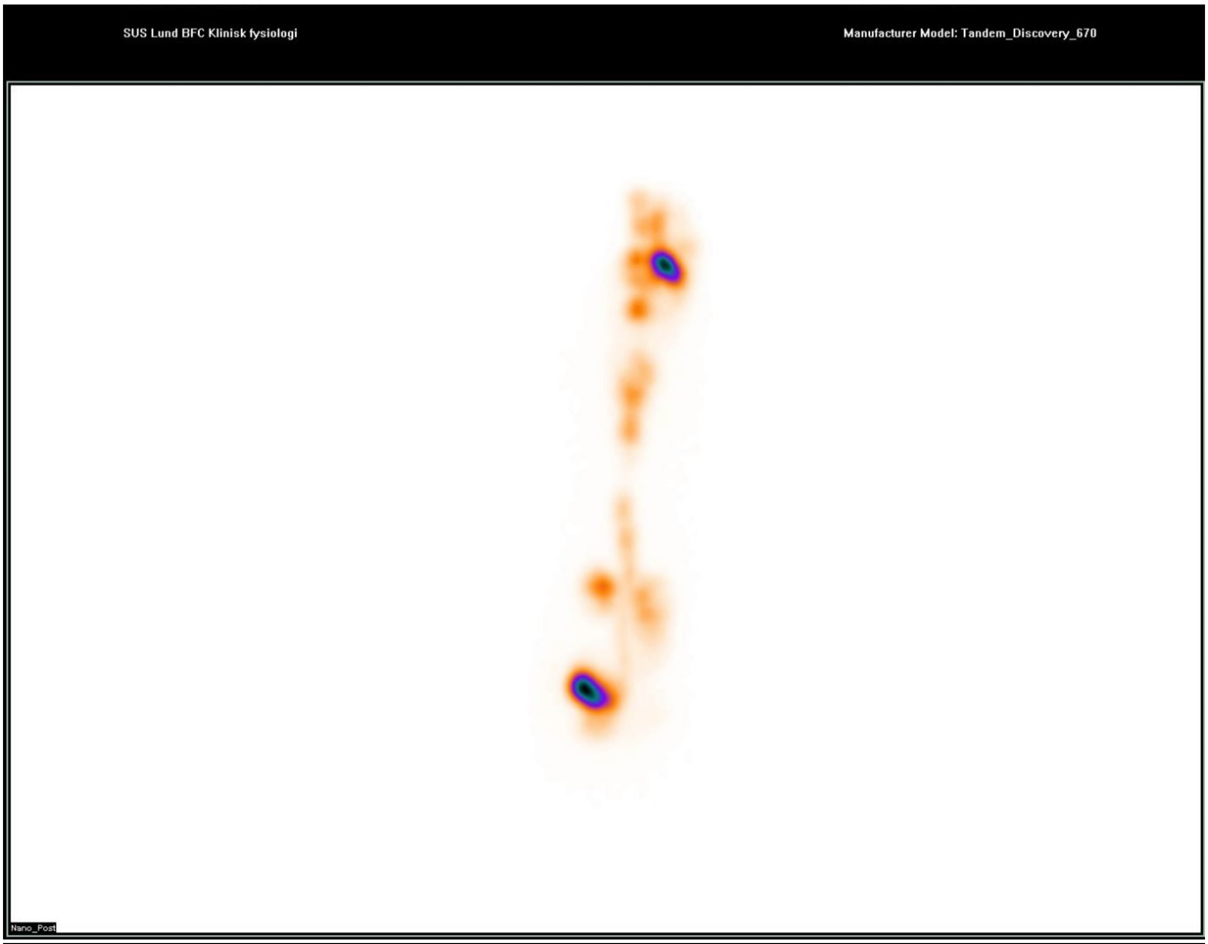
SUS Lund BFC Klinisk fysiologi
Manufacturer Model: Tandem_Discovery_670
Nano_Post

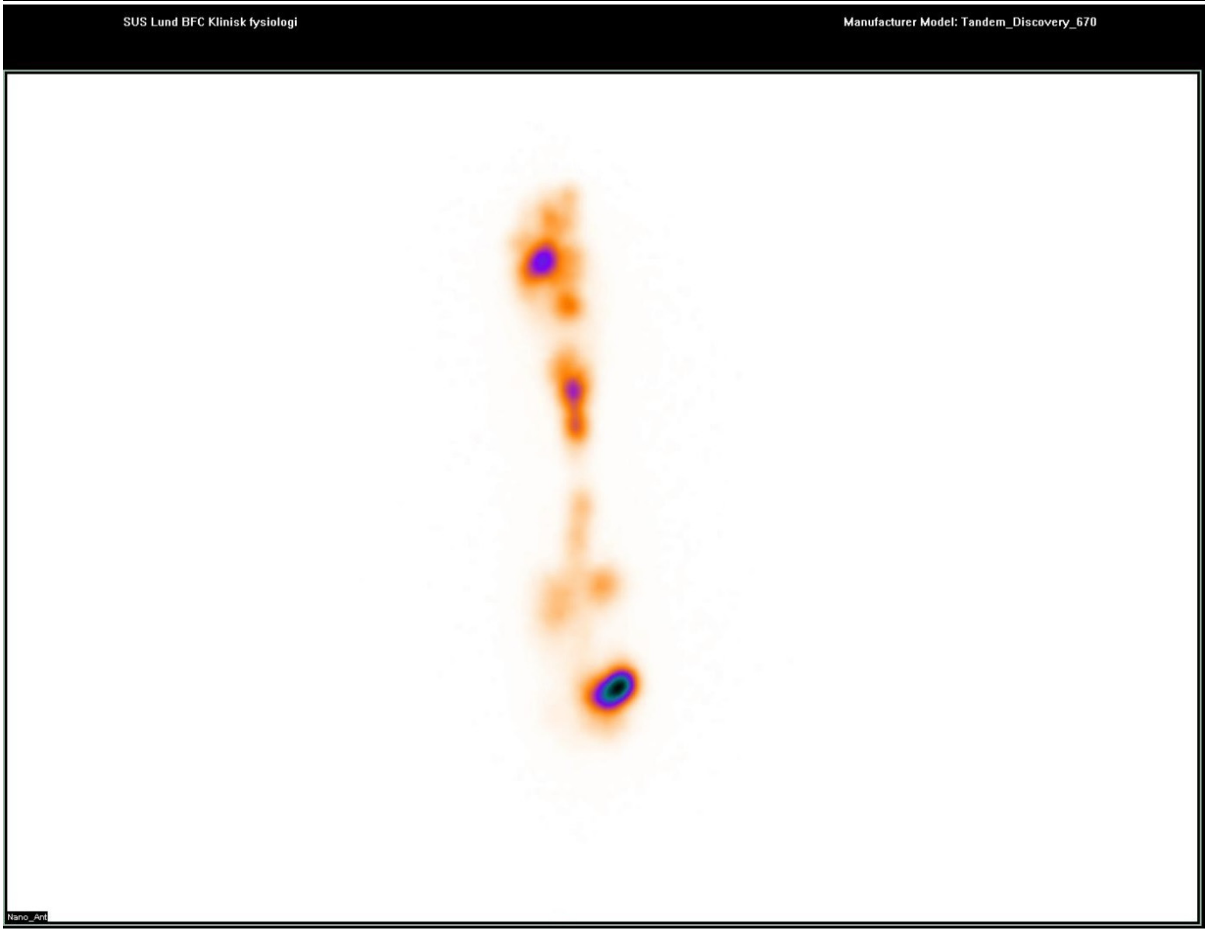
SUS Lund BFC Klinisk fysiologi
Manufacturer Model: Tandem_Discovery_670
Nano_Ant

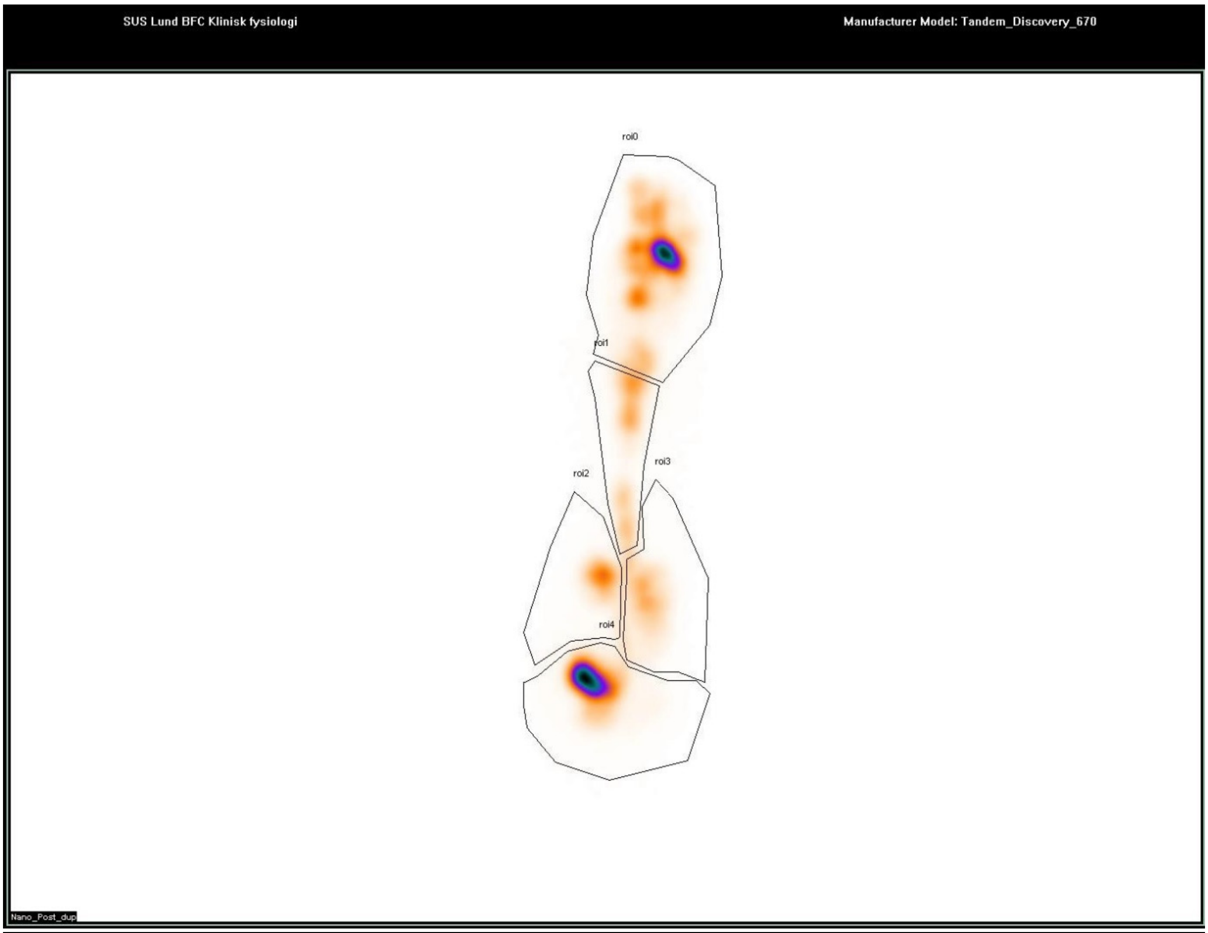
SUS Lund BFC Klinisk fysiologi
Manufacturer Model: Tandem_Discovery_670
roi0
roi1
roi2
roi3
roi4
Nano_Post_dup

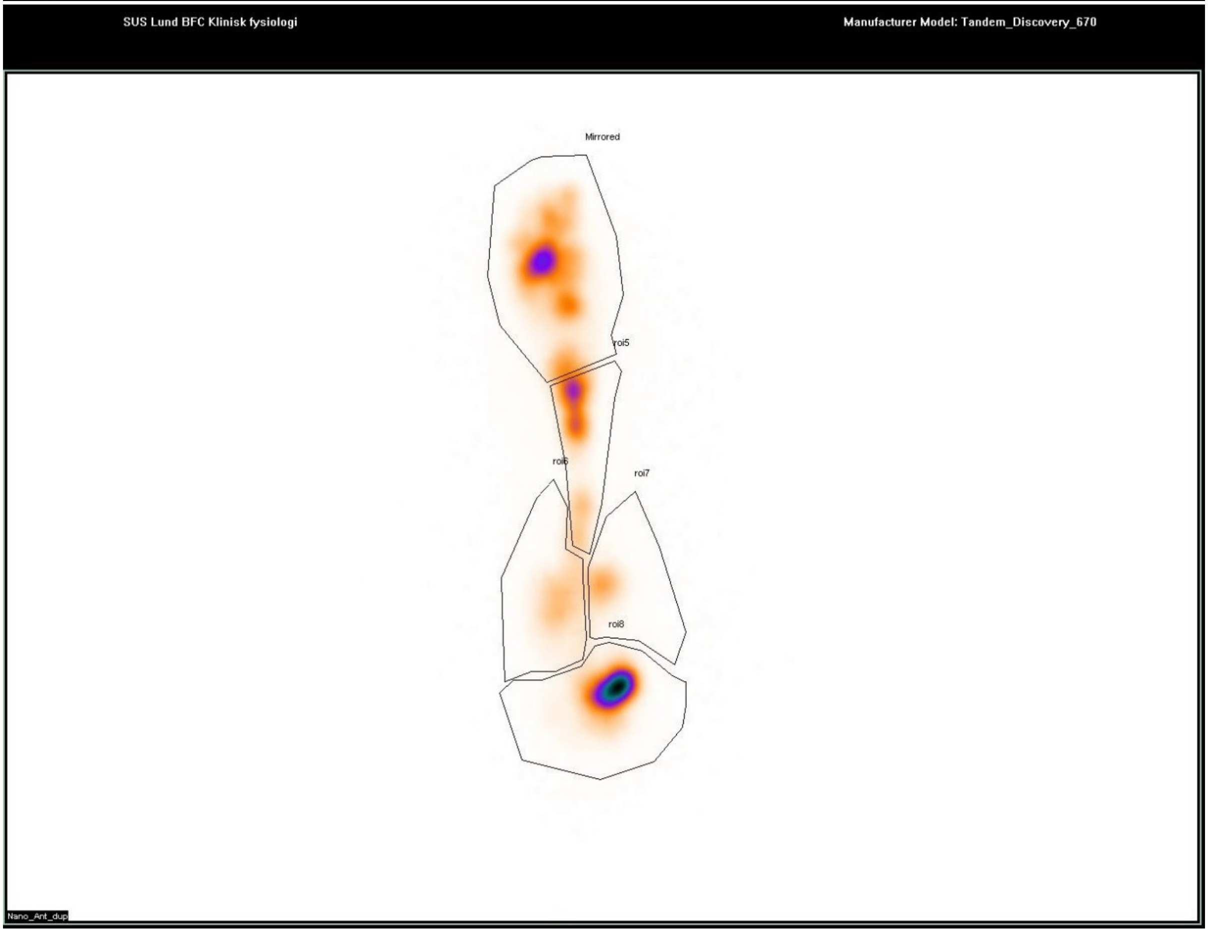
SUS Lund BFC Klinisk fysiologi
Manufacturer Model: Tandem_Discovery_670
Mirrored
roi5
roi6
roi7
roi8
Nano_Ant_dup

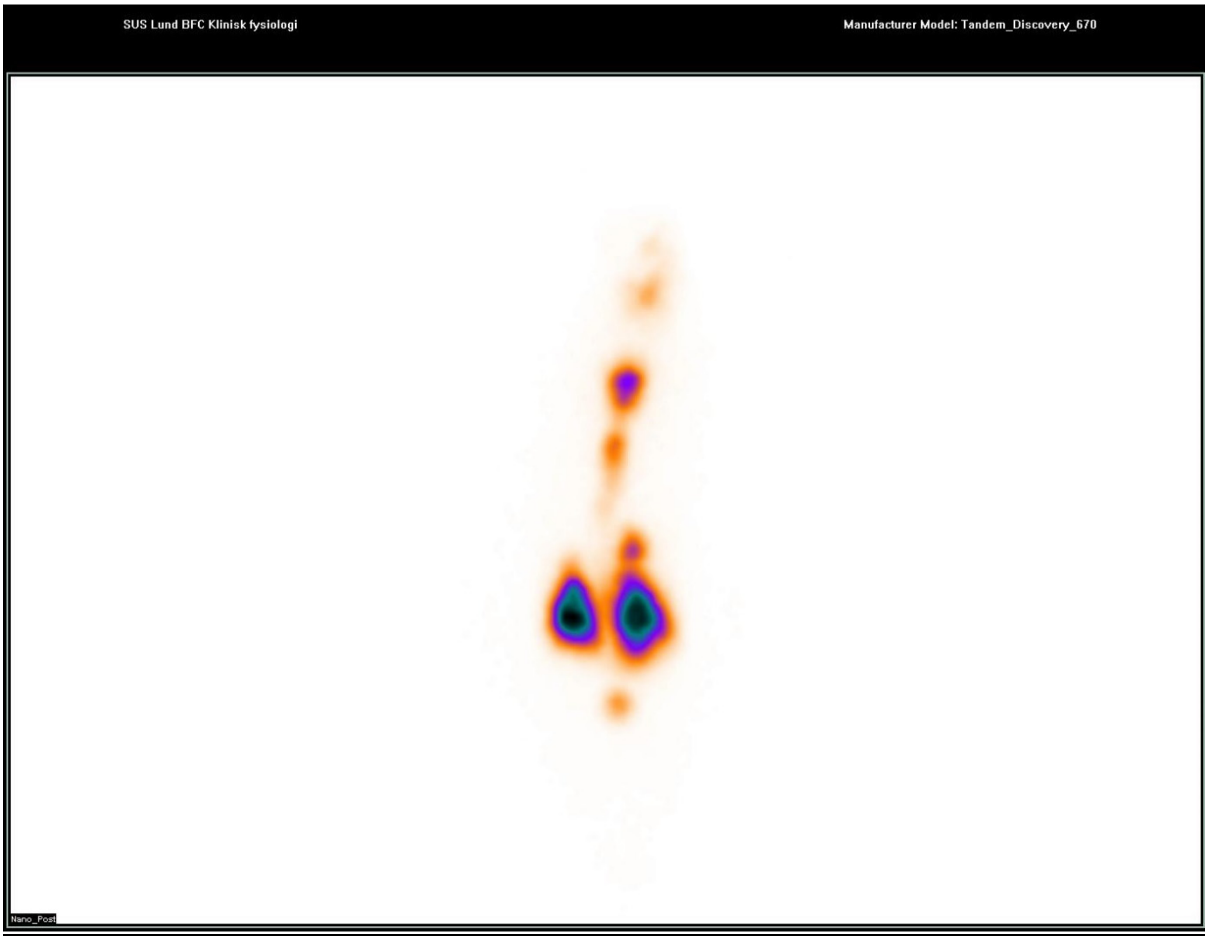
SUS Lund BFC Klinisk fysiologi
Manufacturer Model: Tandem_Discovery_670
Nano_Post

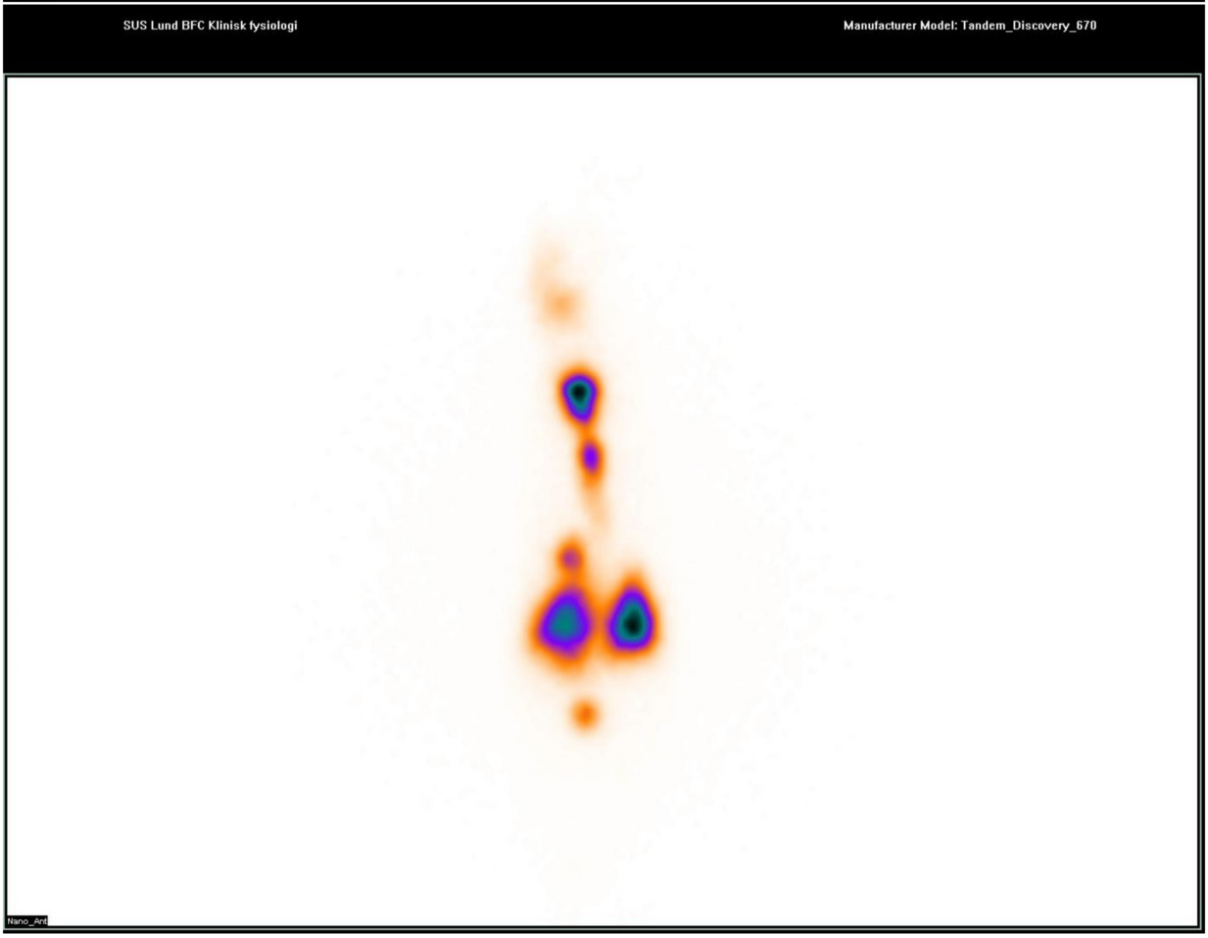
SUS Lund BFC Klinisk fysiologi
Manufacturer Model: Tandem_Discovery_670
Nano_Ant

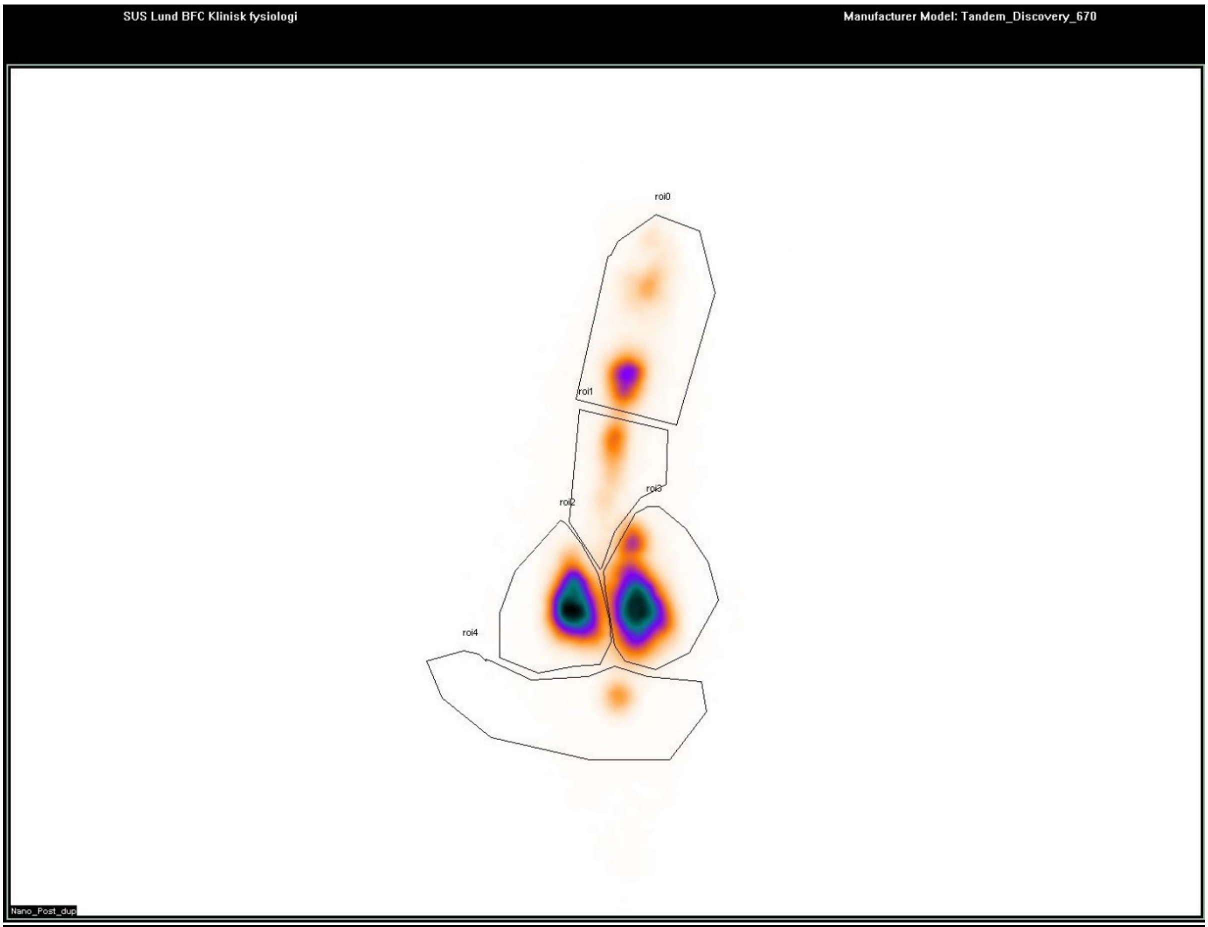
SUS Lund BFC Klinisk fysiologi
Manufacturer Model: Tandem_Discovery_670
roi0
roi1
roi2
roi3
roi4
Nano_Post_dup

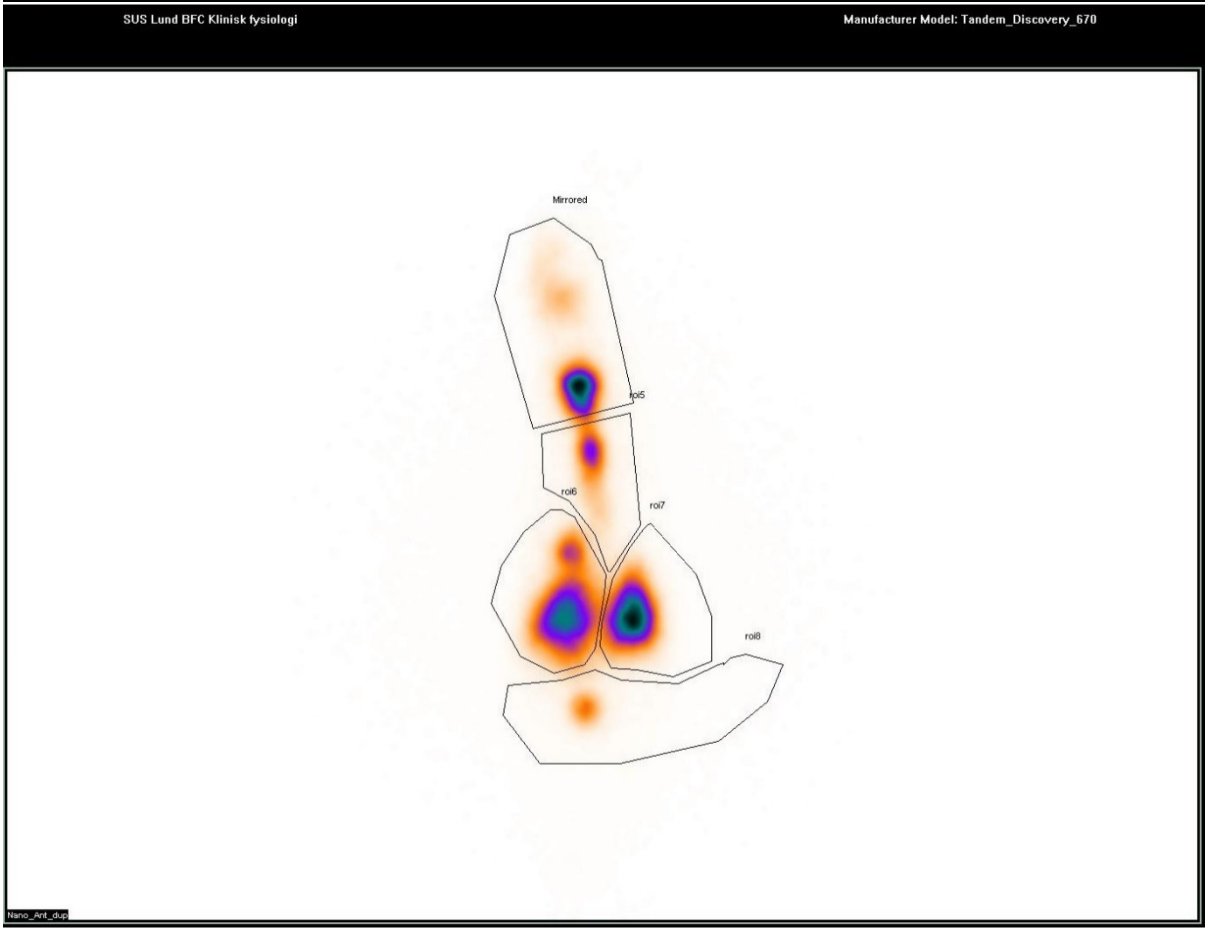
SUS Lund BFC Klinisk fysiologi
Manufacturer Model: Tandem_Discovery_670
Mirrored
roi5
roi6
roi7
roi8
Nano_Ant_dup

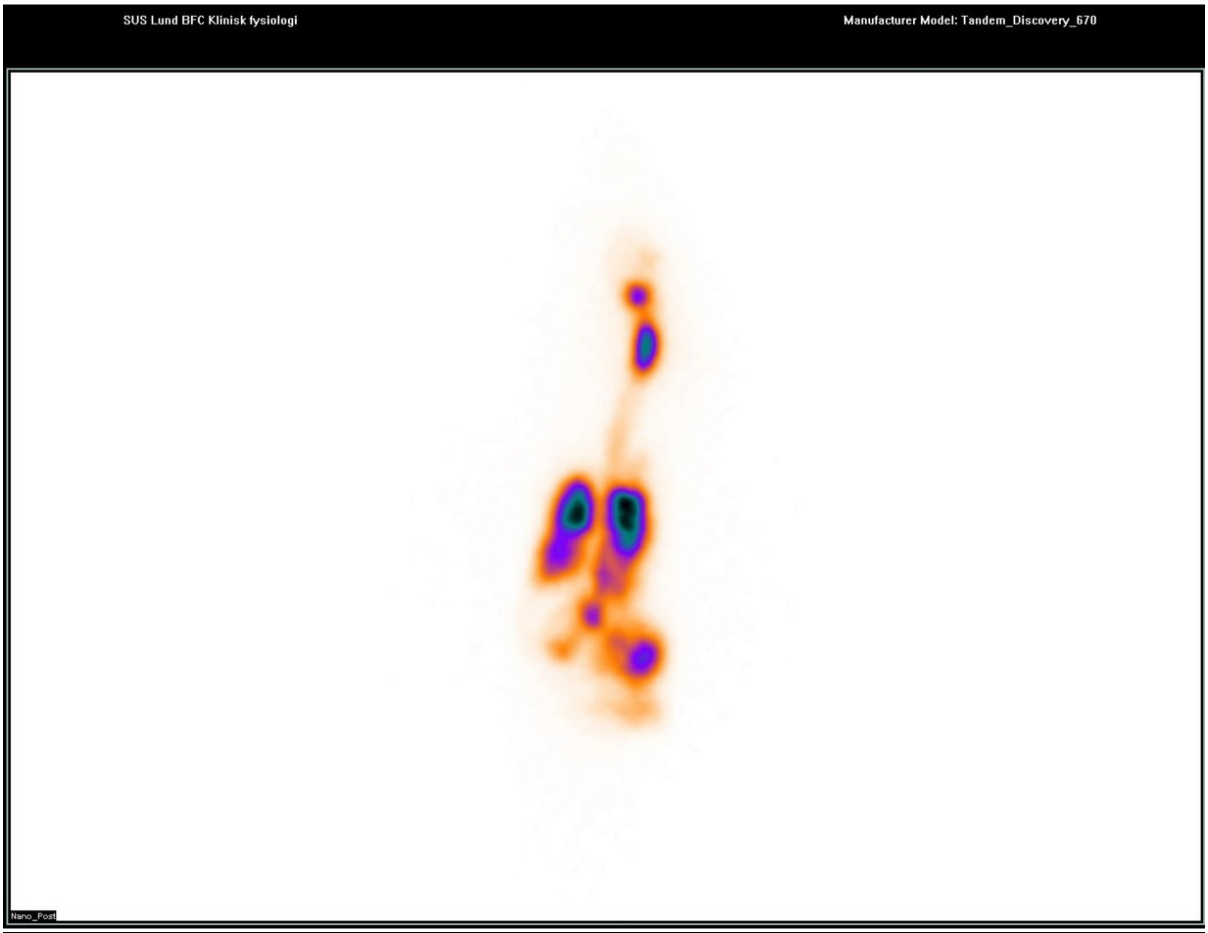
SUS Lund BFC Klinisk fysiologi
Manufacturer Model: Tandem_Discovery_670
Nano_Post

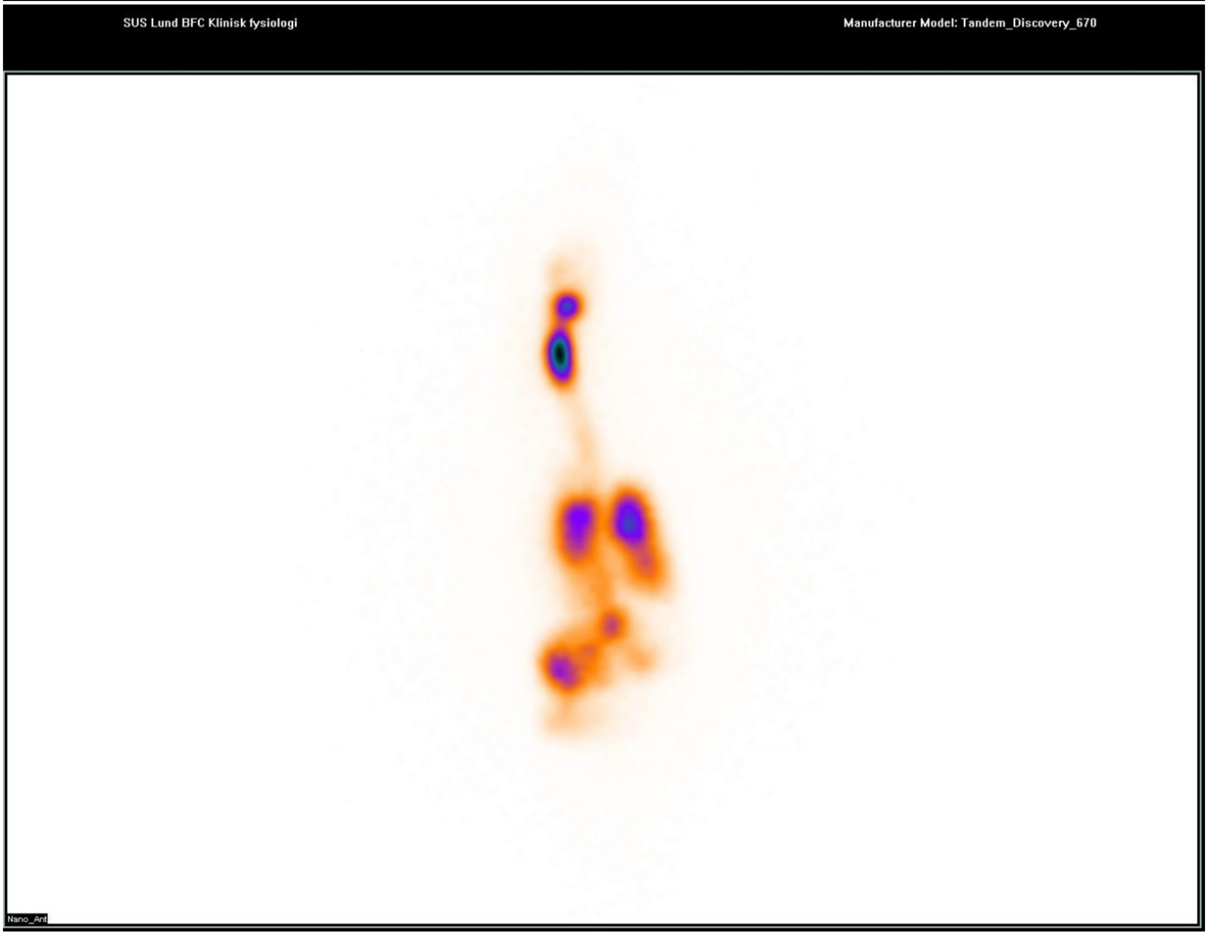
SUS Lund BFC Klinisk fysiologi
Manufacturer Model: Tandem_Discovery_670
Nano_Ant

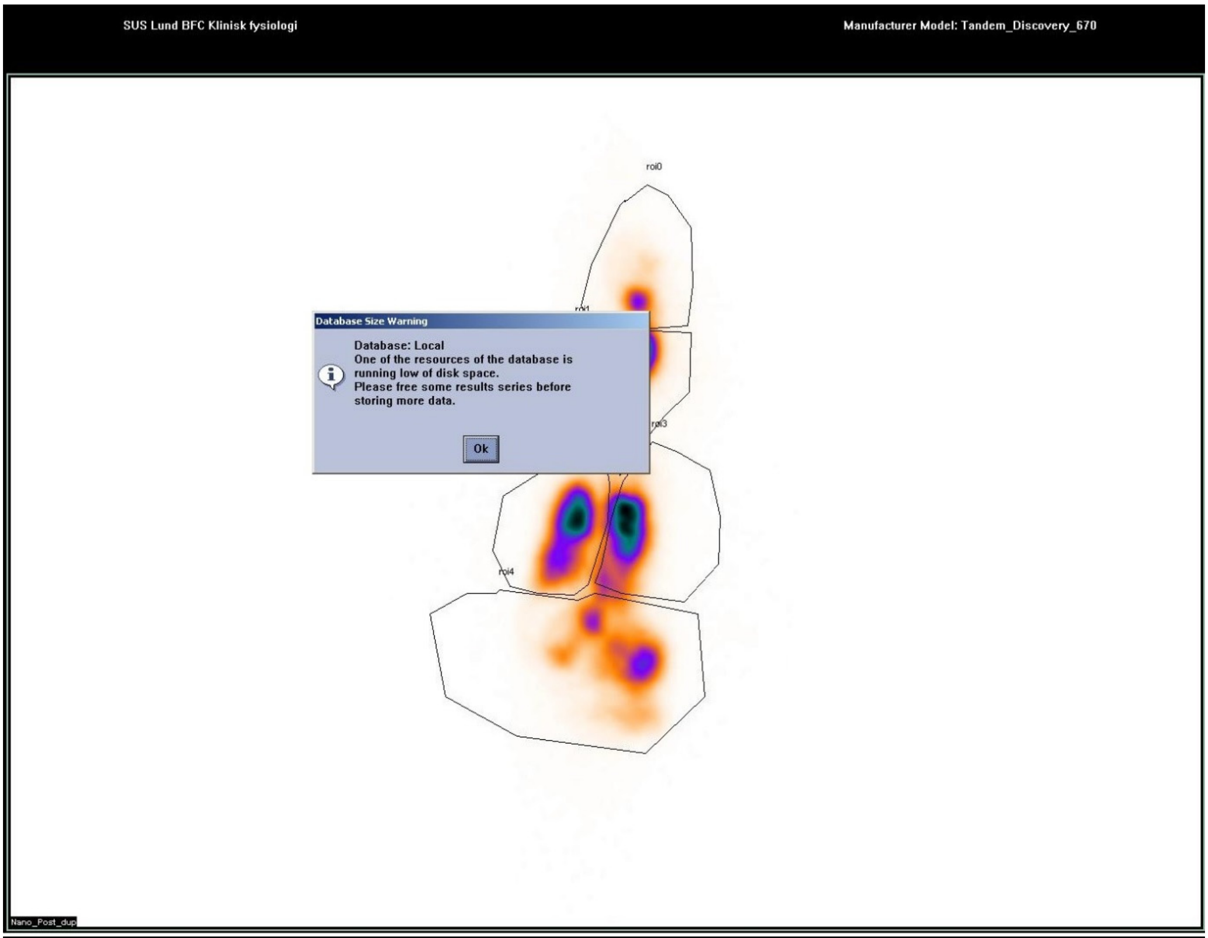
SUS Lund BFC Klinisk fysiologi
Manufacturer Model: Tandem_Discovery_670
roi0
Database Size Warning
Database: Local
One of the resources of the database is
running low of disk space.
Please free some results series before
storing more data.
Ok
roi3
roi4
Nano_Post_dup

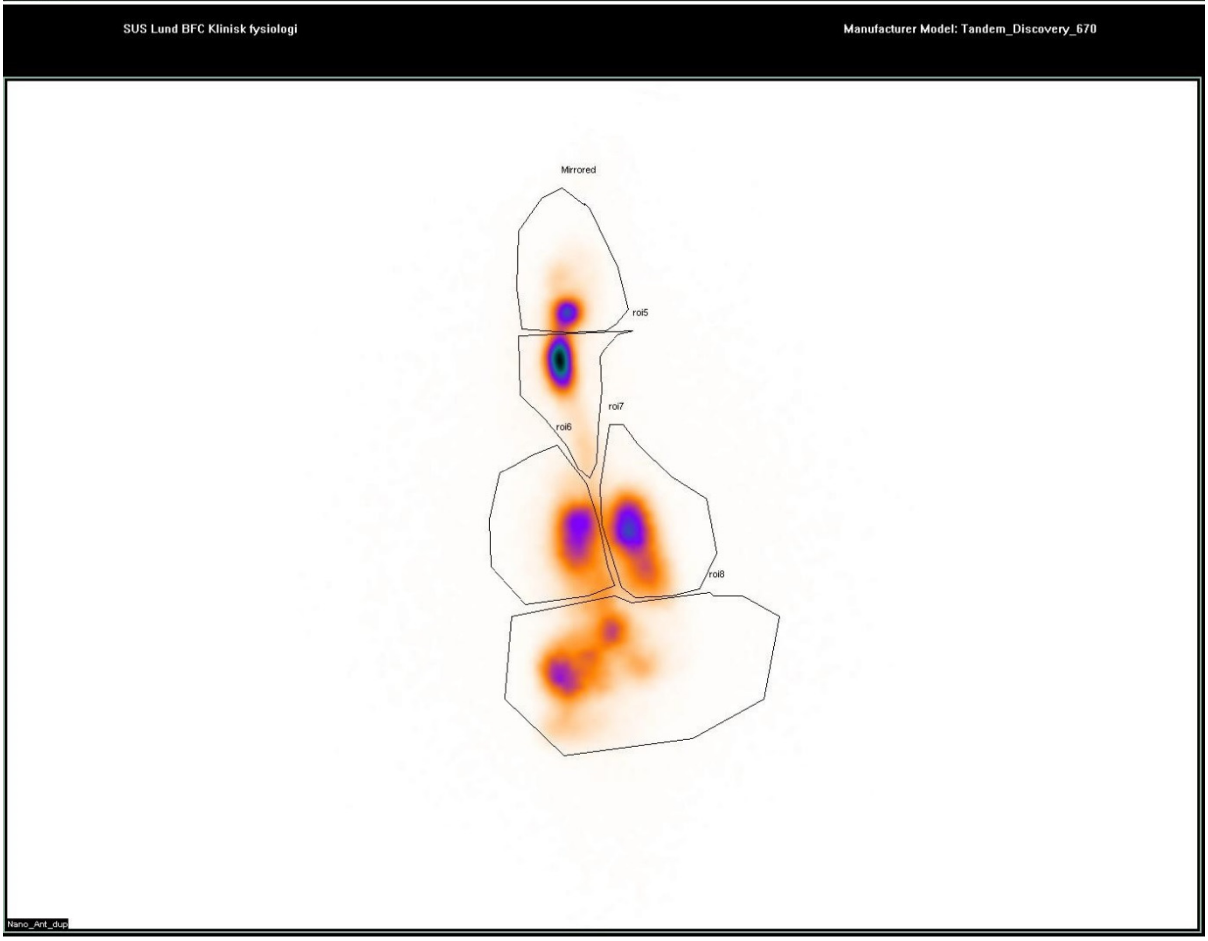
SUS Lund BFC Klinisk fysiologi
Manufacturer Model: Tandem_Discovery_670
Mirrored
roi5
roi7
roi6
roi8
Nano_Ant_dup

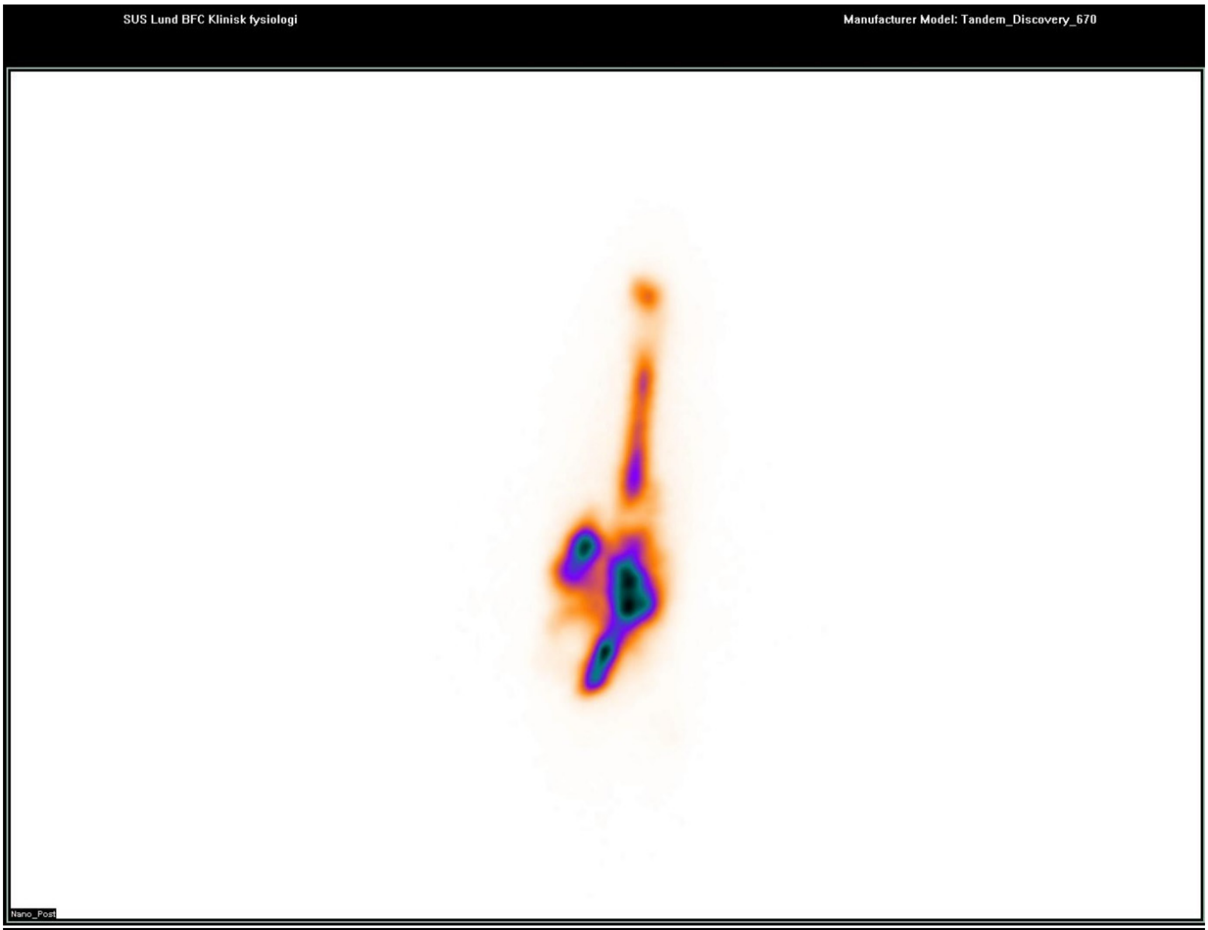
SUS Lund BFC Klinisk fysiologi
Manufacturer Model: Tandem_Discovery_670
Nano_Post

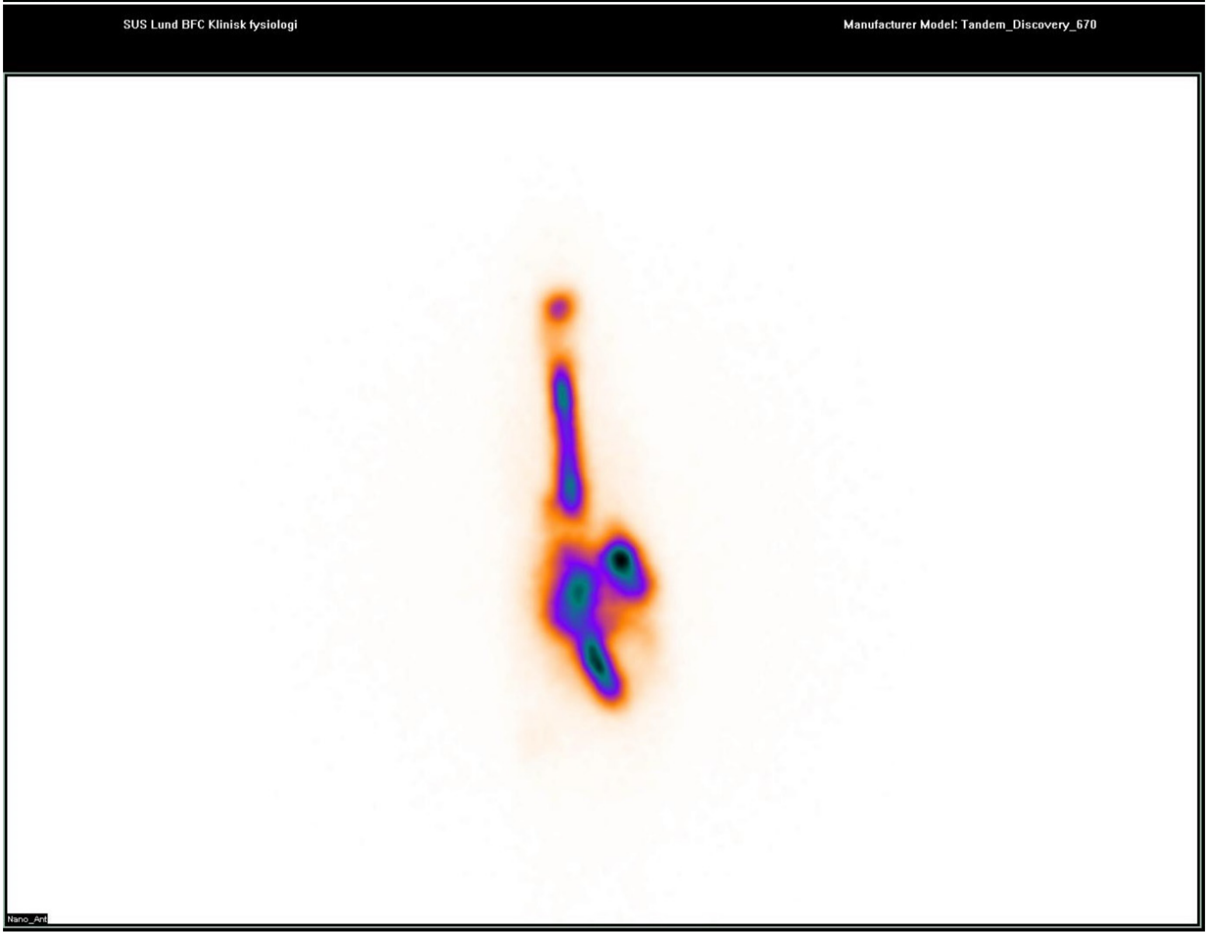
SUS Lund BFC Klinisk fysiologi
Manufacturer Model: Tandem_Discovery_670
Nano_Ant

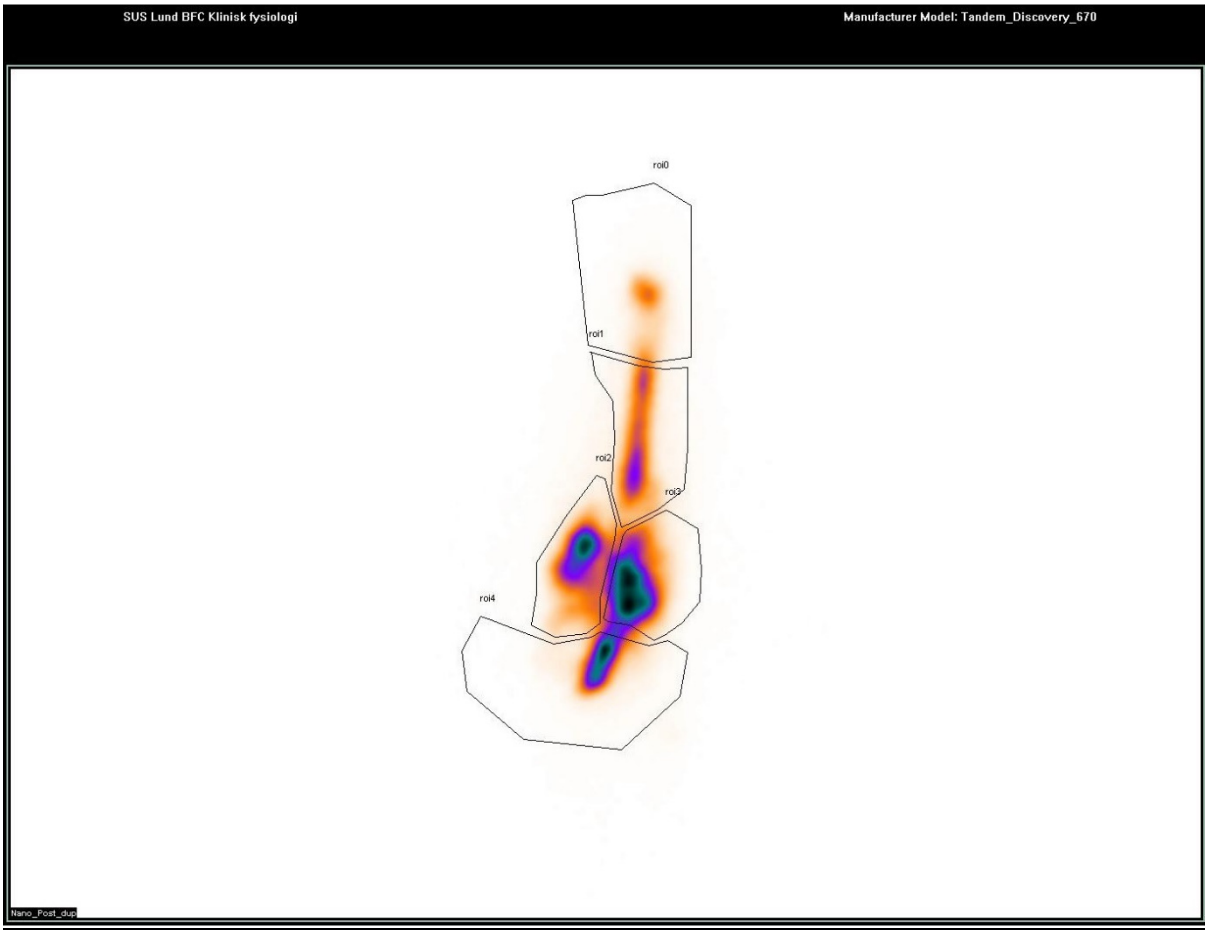

SUS Lund BFC Klinisk fysiologi
Manufacturer Model: Tandem_Discovery_670
roi0
roi1
roi2
roi3
roi4
Nano_Post_dup

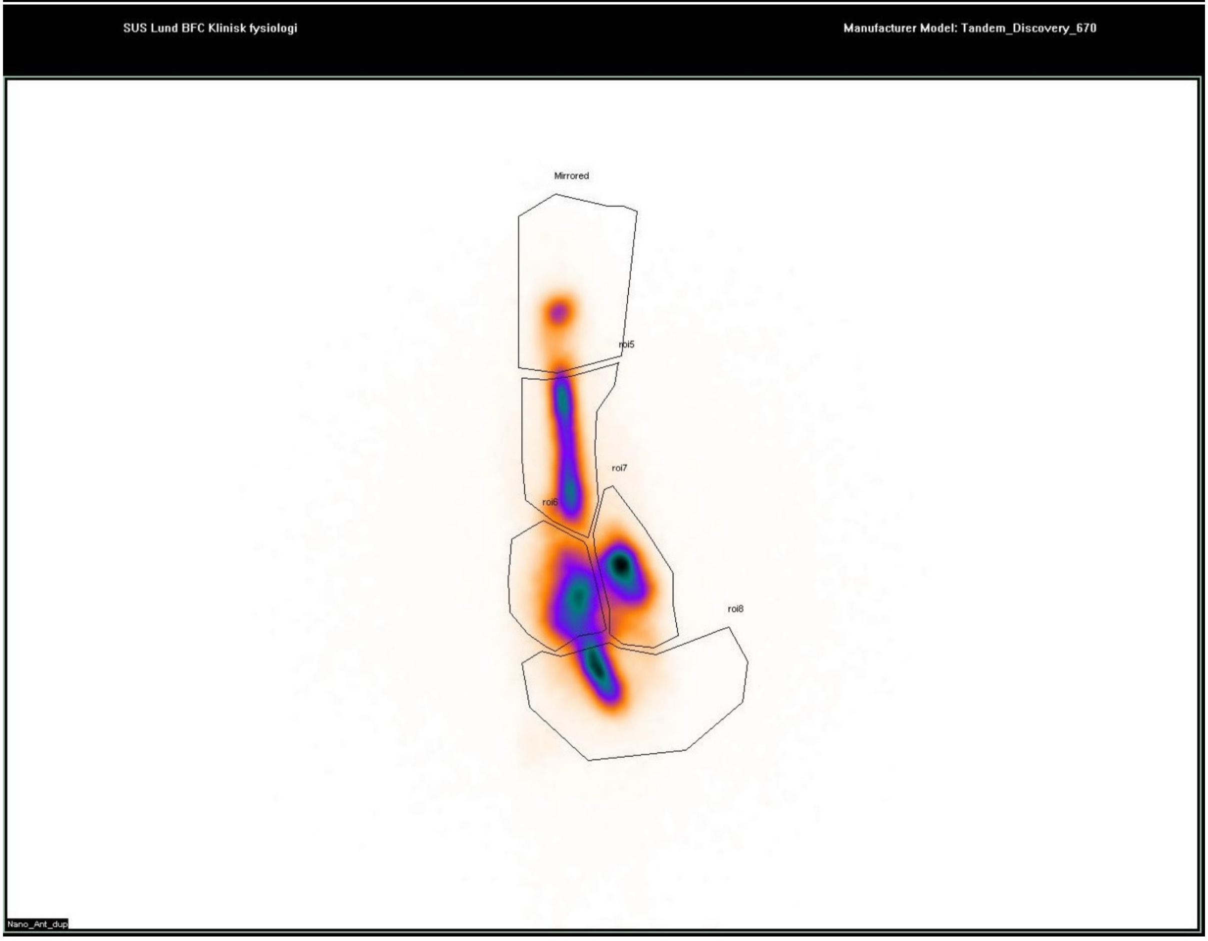

SUS Lund BFC Klinisk fysiologi
Manufacturer Model: Tandem_Discovery_670
Mirrored
roi5
roi7
roi6
roi8
Nano_Ant_dup

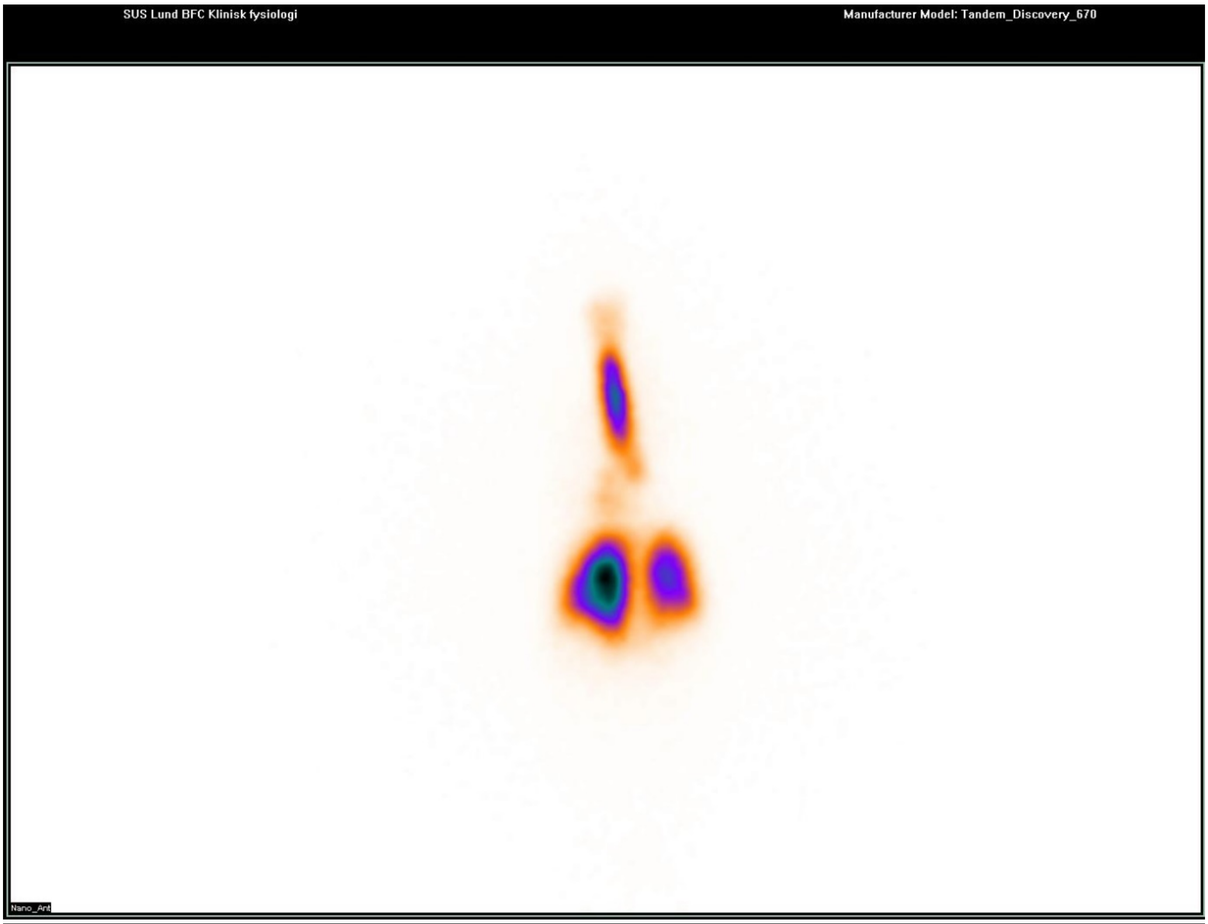
SUS Lund BFC Klinisk fysiologi
Manufacturer Model: Tandem_Discovery_670
Nano_Ant

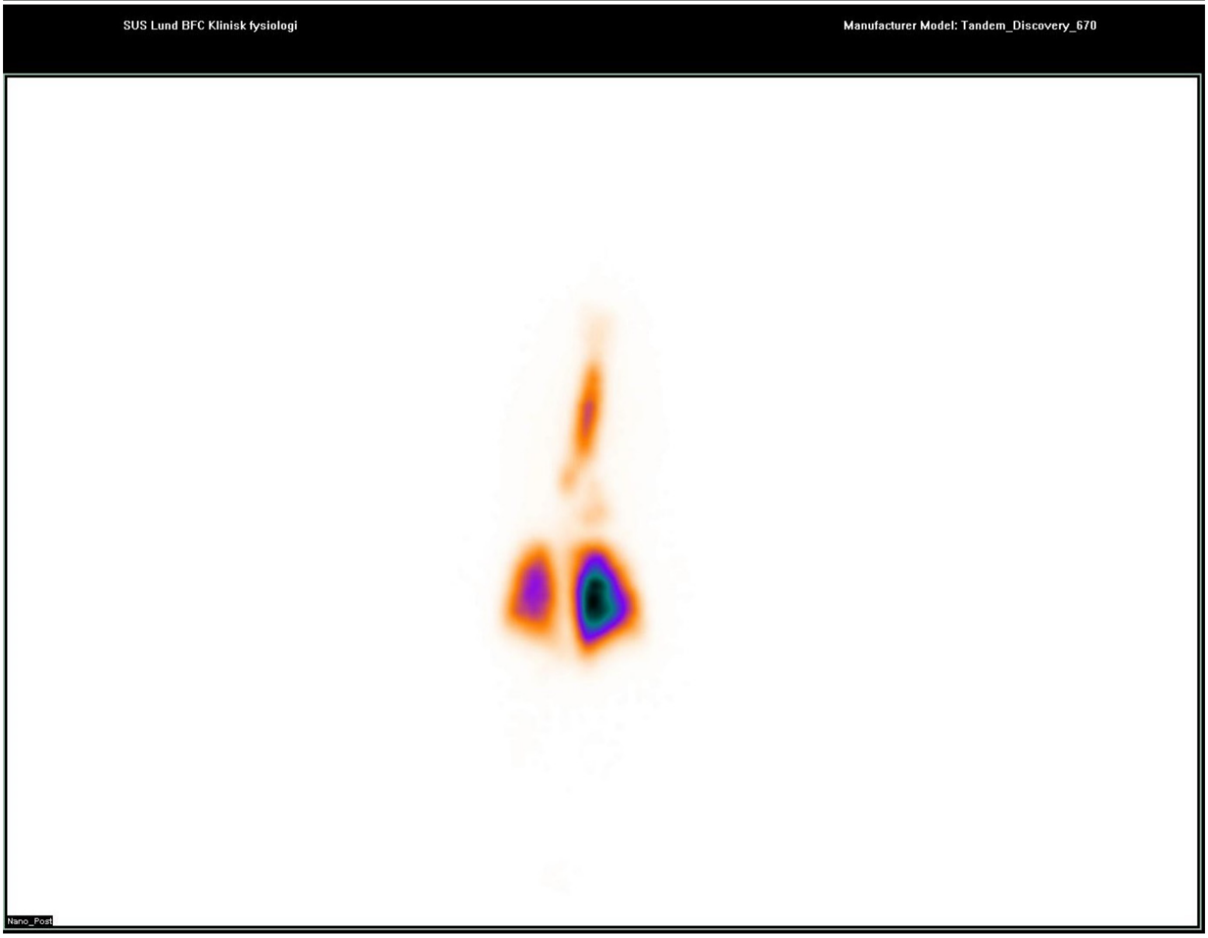
SUS Lund BFC Klinisk fysiologi
Manufacturer Model: Tandem_Discovery_670
Nano_Post

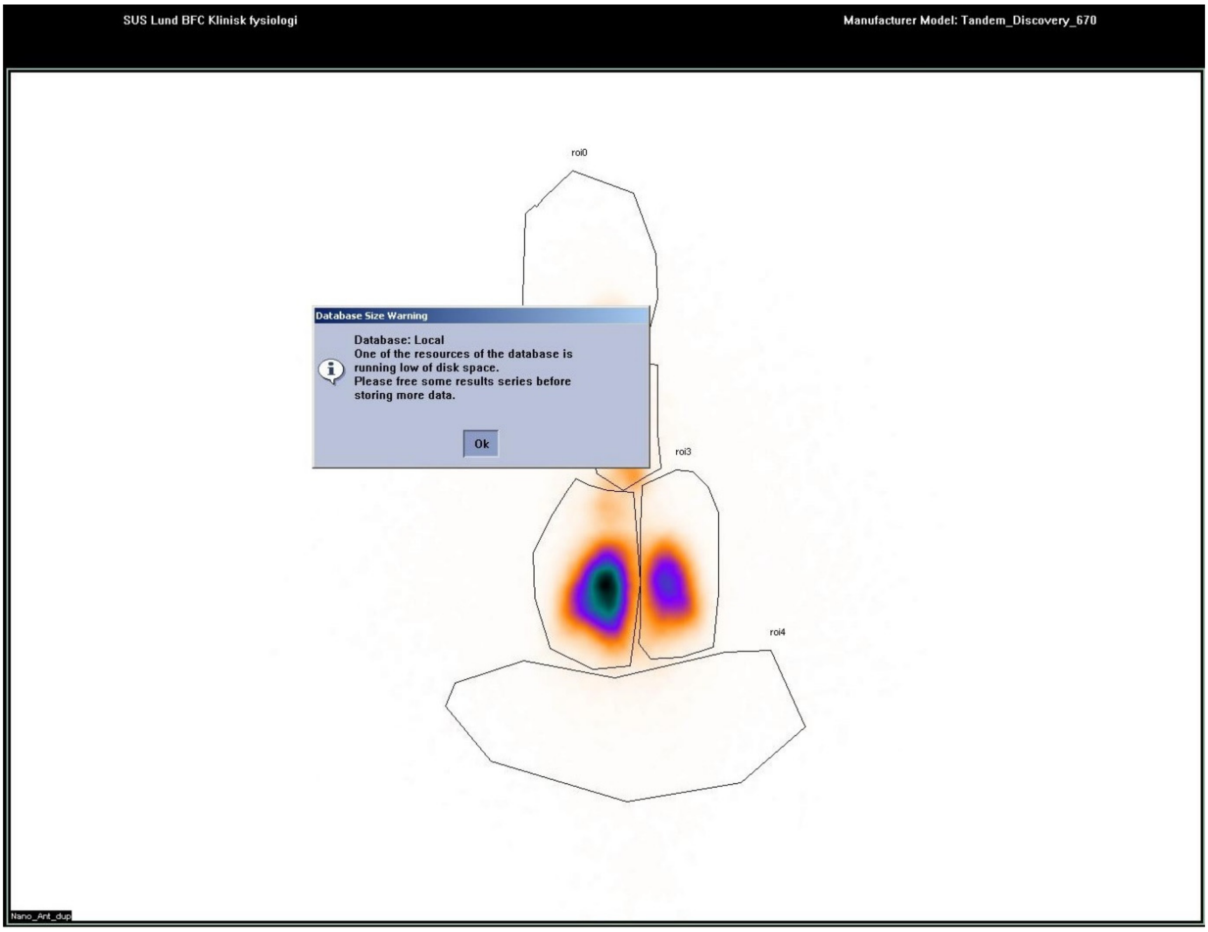
SUS Lund BFC Klinisk fysiologi
Manufacturer Model: Tandem_Discovery_670
roi0
Database Size Warning
Database: Local
One of the resources of the database is running low of disk space.
Please free some results series before storing more data.
Ok
roi3
roi4
Nano_Ant_dup

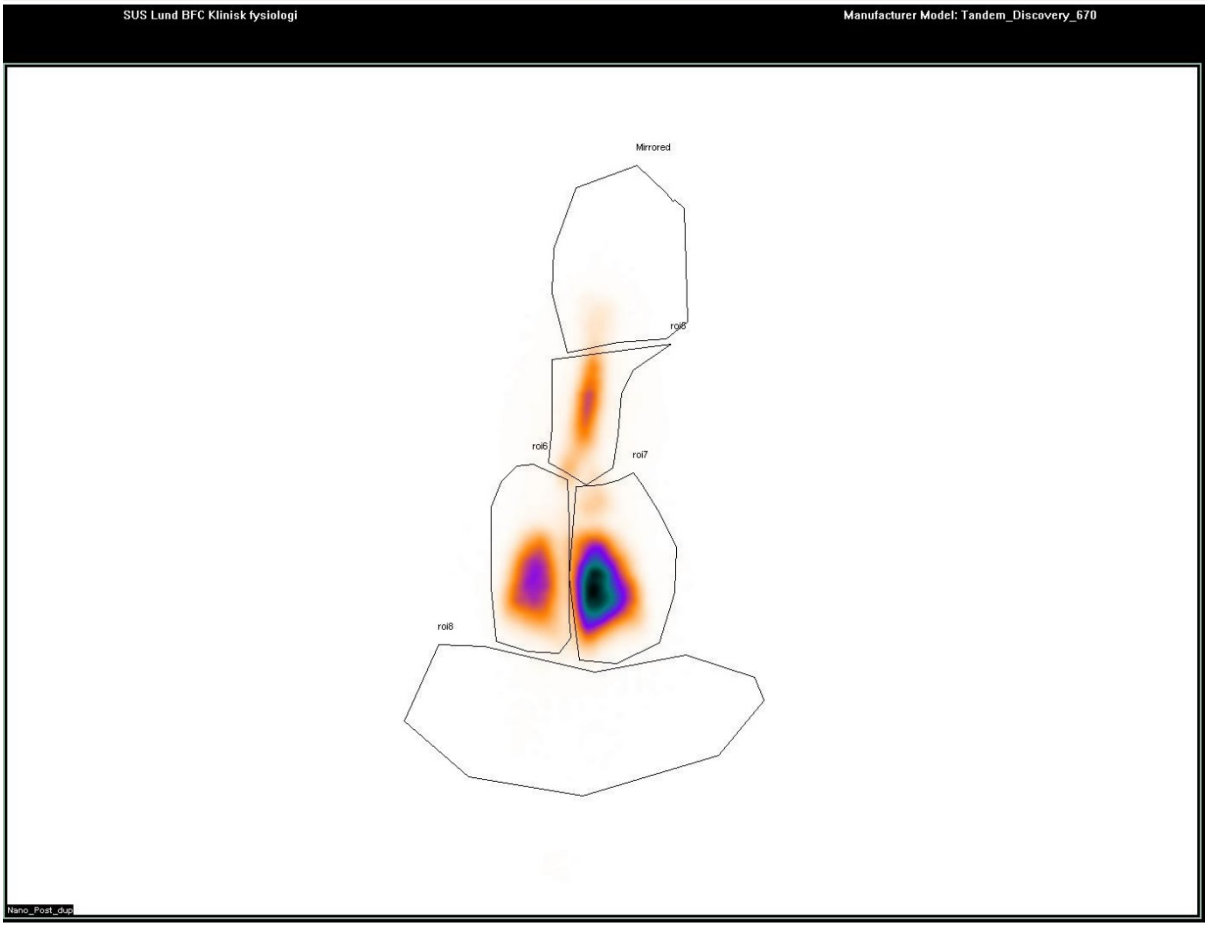
SUS Lund BFC Klinisk fysiologi
Manufacturer Model: Tandem_Discovery_670
Mirrored
roi5
roi6
roi7
roi8
Nano_Post_dup
